# Cross-Talk Between Cancer and Its Cellular Environment—A Role in Cancer Progression

**DOI:** 10.3390/cells14060403

**Published:** 2025-03-10

**Authors:** Eliza Turlej, Aleksandra Domaradzka, Justyna Radzka, Dominika Drulis-Fajdasz, Julita Kulbacka, Agnieszka Gizak

**Affiliations:** 1 Departament of Molecular Physiology and Neurobiology, University of Wrocław, ul. Sienkiewicza 21, 50-335 Wrocław, Poland; eliza.turlej@uwr.edu.pl (E.T.); aleksandra.domaradzka@uwr.edu.pl (A.D.); justyna.radzka@uwr.edu.pl (J.R.); 2 Departament of Molecular and Cellular Biology, Faculty of Pharmacy, Wrocław Medical University, Borowska 211A, 50-556 Wrocław, Poland; julita.kulbacka@umw.edu.pl; 3 Department of Immunology and Bioelectrochemistry, State Research Institute Centre for Innovative Medicine, LT-08406 Vilnius, Lithuania

**Keywords:** cancer, cancer-associated cells, tumor microenvironment

## Abstract

The tumor microenvironment is a dynamic and complex three-dimensional network comprising the extracellular matrix and diverse non-cancerous cells, including fibroblasts, adipocytes, endothelial cells and various immune cells (lymphocytes T and B, NK cells, dendritic cells, monocytes/macrophages, myeloid-derived suppressor cells, and innate lymphoid cells). A constantly and rapidly growing number of studies highlight the critical role of these cells in shaping cancer survival, metastatic potential and therapy resistance. This review provides a synthesis of current knowledge on the modulating role of the cellular microenvironment in cancer progression and response to treatment.

## 1. Introduction

Since the 1950s, the tumor microenvironment (TME) has been recognized as a key regulator of cancer progression, influencing cell proliferation, therapy resistance, invasion, metastasis, inflammation, and immune evasion. The TME comprises fibroblasts, adipocytes, endothelial cells, and diverse immune cells, including T and B lymphocytes, NK cells, dendritic cells, monocytes/macrophages, and myeloid-derived suppressor cells (MDSCs), along with extracellular matrix (ECM) components, chemokines, cytokines, and growth factors [1].

The TME facilitates angiogenesis, nutrient supply, and metabolic waste management, primarily benefiting tumor growth and spread. However, in early tumorigenesis, it can exert anti-cancer effects by promoting inflammation, enabling immune-mediated elimination of cancer cells. A metabolic tug-of-war exists between cancer cells and the TME; the high metabolic activity of cancer cells depletes nutrients, impairing immune surveillance and fostering tumor survival. Hypoxia, reactive oxygen species (ROS), and metabolic byproducts can create a toxic environment for normal cells, further driving immune evasion and tumor progression [2,3].

As cancer advances, it disrupts the number of cancer-suppressing cells within the TME through metabolic competition, reduced antigen presentation, and immunosuppressive cytokines and chemokines release. Given its pivotal role in tumor growth and therapy resistance, targeting the TME represents a promising strategy for improving cancer treatment outcomes [4].

**Figure 1:**
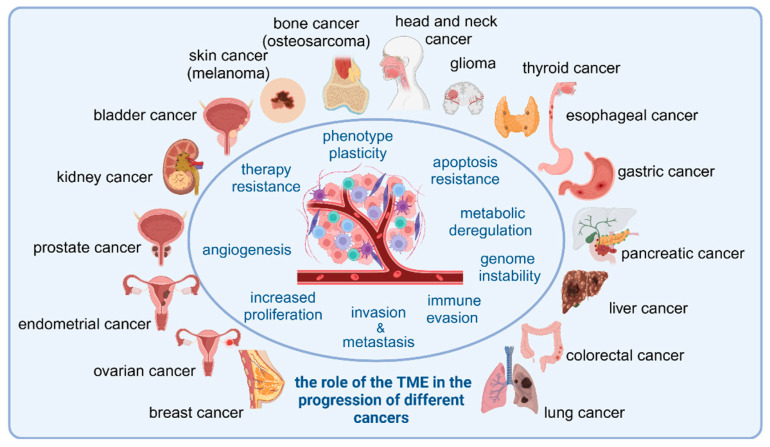
The role of the tumor microenvironment (TME) in cancer progression in tumors of various organs, discussed in this paper.

This review summarizes the latest findings regarding the role of TME components in various cancer types (as presented in Figure 1).

## 2. Cells in Cancer Microenvironment

**Figure 2:**
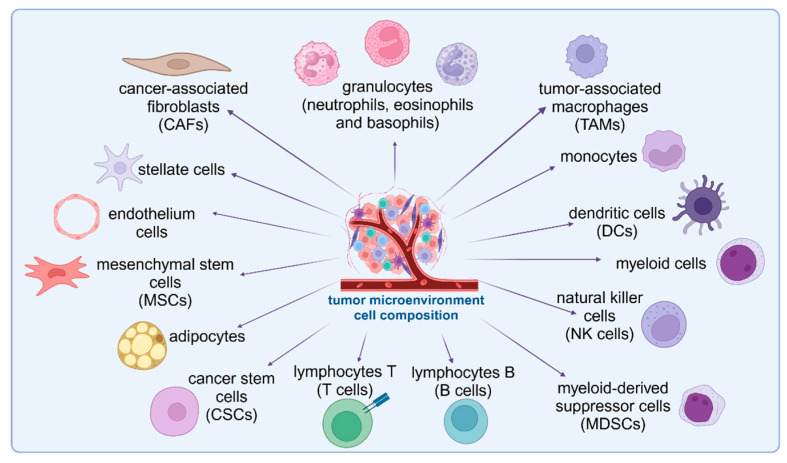
Cellular composition of the tumor microenvironment. The TME is composed of cells of different origins that exert complex influences on growth, progression, and sensitivity to therapy.

The cells of immune and non-immune origin that constitute the TME which are the subject of this publication are summarized in Figure 2.

### 2.1. Non-Immune Components of the TME

#### 2.1.1. Cancer-Associated Fibroblasts

Fibroblasts are functionally diverse cells that exist in both quiescent and active states. Quiescent fibroblasts play a crucial role in maintaining the normal structure of tissues. Their activation occurs in response to tissue damage and also during the process of carcinogenesis [5,6,7]. Fibroblasts express a wide range of specific markers, such as α-smooth muscle actin (α-SMA), fibroblast activation protein (FAP), a type II serine membrane protease expressed on the surface of fibroblasts, periostin, and platelet-derived growth factor receptor β (PDGFR-β), which allow for the distinction of various fibroblast subtypes [8].

Cancer-associated fibroblasts (CAFs) are fibroblasts located within or near the tumor. They are a major component of tumor stroma. CAFs play an important role in shaping the TME. CAFs can interact with ECM components, cancer cells, and also affect other cells in the TME. They interact with endothelial cells to release growth factor promoting angiogenesis, and with immune suppressive cells (e.g., myeloid-derived suppressor cells and M2 macrophages) via cytokines, chemokines, microRNAs (miRNAs), long non-coding RNAs (lncRNAs) [9,10]. This will be discussed in details in subsequent chapters.

The most distinctive function of CAFs is their ability to synthesize and remodel the ECM. CAFs secrete ECM-building components such as type I, III, IV, and V collagen, fibrinolytic proteins, hyaluronic acid, laminins, elastin, proteoglycans and glycosaminoglycans [11,12]. Additionally, they degrade the ECM by secreting proteases, including matrix metalloproteinases (MMPs) and urokinase-type plasminogen activator. This allows for remodeling of the TME by influencing tissue stiffening and stromal fibrosis [13,14,15]. The rigid structure of the TME provides a suitable environment for intercellular interactions but also prevents immune cells from infiltrating and causes resistance to therapies by hindering drug penetration into the tumor. This leads to increased tumor migration, invasion, and malignancy [16,17,18].

CAFs influence tumor cell survival and proliferation. They control metastasis formation through the secretion of transforming growth factor β (TGF-β) required for epithelial–mesenchymal transition (EMT) and angiogenesis and facilitate tumor cell migration through the secretion of MMP3, which promotes tumor cell invasion. They promote immunosuppression by producing various chemokines and cytokines that modulate the immune response [9,19].

CAFs originate from various cell types. Resident tissue fibroblasts are one of the main sources of CAFs. The recruitment and stimulation of quiescent CAFs are modulated by factors such as TGF-β stromal cell-derived factor-1 (SDF-1), hepatic growth factor (HGF), platelet-derived growth factor (PDGF) and reactive oxygen species (ROS). In addition, CAFs can also originate from epithelial cells, endothelial cells, mesenchymal stem cells, stellate cells, pericytes, adipocytes, and bone marrow-derived cells. The diversity of CAFs sources accounts for the high heterogeneity of CAFs in terms of the expression of different markers and performing various functions within the tumor microenvironment [5,20,21,22,23,24,25].

**Figure 3:**
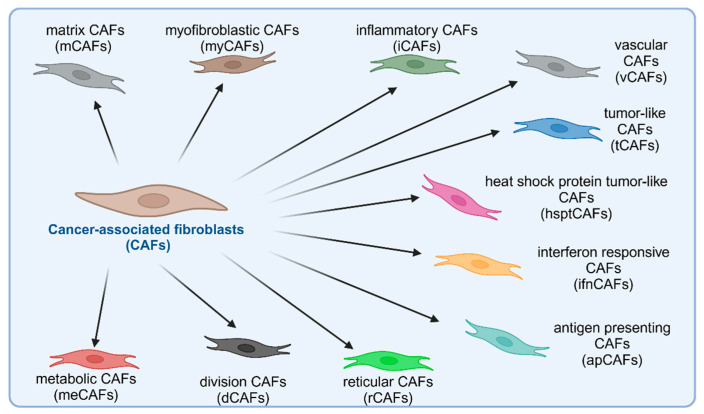
Roles of cancer-associated fibroblasts (CAFs) in the tumor microenvironment. The diversity of CAF sources contributes to their high heterogeneity in terms of the expression of different markers and performing different functions in the TME.

Thus, tumor tissue may contain: matrix CAFs (mCAFs), myofibroblastic CAFs (myCAFs), inflammatory CAFs (iCAFs), vascular CAFs (vCAFs), tumor-like CAFs (tCAFs), heat shock protein tumor-like CAFs (hsptCAFs), interferon-responsive CAFs (ifnCAFs), antigen-presenting CAFs (apCAFs), reticular CAFs (rCAFs), division CAFs (dCAFs) and metabolic CAFs (meCAFs) as shown in Figure 3 [26].

mCAFs are characterized by high expression levels of genes encoding matrix proteins such as MMP11 and COL10A1, COL11A1, COL8A1, COL1A2, COL12A1, COL3A1, non-collagenous matrix proteins COMP and POSTN, adhesion-related proteins such as LRRC15, LRRC17 and ASPN, and migration-related proteins such as SULF1, INHBA and VCAN. TGF-β, KRAS and EMT-relevant signaling pathways are upregulated in mCAFs cells. mCAFs are responsible for ECM modification, collagen synthesis, and are also involved in focal adhesion signaling pathways [26,27].

myCAFs are typically located near cancer cells. Markers specific to myCAFs include TAGLN, MYL9, TPM1, TPM2, MMP11, and POSTN. myCAFs are also characterized by high expression of αSMA, COL1A1 and low levels of IL-6 [27,28,29,30].

iCAFs are usually found at the tumor periphery in desmoplastic regions (fibrous connective tissue surrounding the cancer) and have low levels of αSMA. iCAFs are characterized by high levels of cytokines and chemokines, including IL-6, IL-8, CXCL1, CXCL2, CCL2, and CXCL12, as well as the expression of complement factor D (CFD) and C3, and matrix proteins such as lamin A/C (LMNA) and dermatopontin (DPT). They exhibit specific expression of genes like hyaluronan synthases (*HAS1* and *HAS2*), encoding the angiotensin II type 1 receptor (*AGTR1*), and a phospholipase encoded by *PLA2G2A*. A hallmark of iCAFs is the upregulation of the IL-6-JAK-STAT3 KRAS TNF/NF-κB, IL-2/STAT5 pathways and IFN-γ response and complement signaling pathways [26,27].

vCAFs show high expression of NOTCH3, a receptor important in vascularization and angiogenesis, and collagen COL18A1, involved in the regulation of angiogenesis.

tCAFs show increased expression of genes related to proliferation, migration and metastasis such as *PDPN*, *MME*, *TMEM158* and *NDRG1*, and genes related to stress response such *ENO1* and *GAPDH*. These cells also show high levels of membrane metalloproteinase (MME), TMEM158, an indicator of Ras pathway activation, and VEGFA, which promotes angiogenesis and vascularization. The name of these cells is related to the fact that their gene expression resembles that of tumor cells. CAFs with this phenotype are strongly associated with chemoresistance [26].

hsptCAFs are characterized by high expression of genes encoding heat shock proteins such as *HSPH1* and *HSP90AA1*, as well as genes specific to tCAFs. Therefore, this subtype of CAFs is considered to be tCAFs subjected to higher levels of cellular stress. TGF-β, KRAS, MTORC1, PI3K/Akt/mTOR, P53 signaling, and EMT, glycolysis and hypoxia-related pathways are upregulated in these cells [26].

ifnCAFs show high levels of expression of genes associated with chronic inflammation such as *IL-32* and upregulated genes in response to interferons such as *CXCL9*, *CXCL10*, *CXCL11* and *IDO-1*. These cells show strong upregulation of inflammatory response pathways and responses to interferon-α and interferon-γ, as well as STAT5, TNF-α, IL-6-JAK-STAT3 and KRAS signaling pathways [26].

apCAFs have high expression of genes involved in MHC-II-related antigen presentation, including *HLA-DRA*, *HLADRB1* and *CD74* [26].

rCAFs strongly express CCL21 and CCL19, which are markers of fibroblasts in lymphoid tissues. These proteins facilitate the influx of naïve T cells [26].

dCAFs are characterized by high expression of genes upregulated during cell division such as *TUBA1B* and *MKI67* [26].

meCAFs are characterized by the high expression of secretory calcium-dependent phospholipase A2 (PLA2G2A) Their differentiation is likely dependent on CREB3L1. meCAFs are highly glycolytic cells [31].

For the readers’ convenience, information regarding the specific markers and functions of different CAFs subtypes has been summarized in Table 1.

In different types of cancer, CAFs exist in various subtypes and proportions, which may additionally change depending on the stage of the cancer. What is important, CAFs can transform into other subtypes. For example, treatment with JAK inhibitors can lead to the transformation of iCAFs into myCAFs [7,21,32]. In this chapter, we outlined 11 common types of fibroblasts that are present in various cancers. Additionally, in the following chapters dedicated to different cancers, we also present atypical fibroblasts that are unique to a particular type of cancer.

#### 2.1.2. Stellate Cells

Stellate cells are quiescent stromal cells of mesenchymal origin, located in the liver and pancreas. When tissue is damaged, stellate cells become activated, enter the cell cycle, and transform into myofibroblasts. A characteristic feature of stellate cells is the deposition of vitamin A in lipid droplets. Pancreatic stellate cells (PSCs) and hepatic stellate cells (HSCs) show similar morphological and functional features. Both cell types are capable of expressing protein markers such as desmin and glial fibrillary acidic protein (GFAP). However, the exact expression levels vary greatly. Despite many similarities, there are differences in expression patterns between PSCs and HSCs. PSCs are characterized by higher levels of α7 integrin, hypoxia-inducible factor 1-alpha subunit (HIF1-α), and cytoskeletal components [7,33].

HSCs, which are usually located in the peritubular and portal areas and can account for up to 15% of the liver mass, play an important role in liver physiology and fibrogenesis. During chronic liver injury, quiescent HSCs transform into activated myofibroblasts, which primarily secrete ECM components, and are the main source of liver CAFs. Stimulation of insulin-like growth factor (IGF1) signaling is required for liver stellate cell activation [7,34].

Activated HSCs can evolve, depending on the duration and stage of the disease, into subtypes that include collagen-producing myofibroblasts or proliferative, vascular and inflammatory phenotypes. Some subpopulations of HSCs contribute to fibrosis and tumorigenesis. HSCs produce TGF-β, cytokines, and growth factors that can act on other cells. HSCs express Toll-like receptors and can respond to bacterial infections by detecting lipopolysaccharide, and they also function as antigen-presenting cells (APCs) capable of activating T lymphocytes. They also express programmed death ligand 1 (PD-L1), which can bind to PD-1 present on macrophages, T lymphocytes, and B lymphocytes [7,35,36,37,38].

Pancreatic stellate cells constitute only approximately 5% of the mass of the pancreas. In a healthy pancreas, PSCs cells are typically in a dormant state and reside around acini as lipid-storing cells. However, various factors such as chronic inflammation, oxidative stress, vitamin A deficiency, and increased secretion of IL-1, IL-6, and TGF-β can cause PSCs to transition into an active state. In chronic pancreatitis and pancreatic ductal adenocarcinoma (PDAC), activated PSCs start to exhibit a myofibroblast-like phenotype and deposit collagen fibers and contribute to the development of pancreatic fibrosis. PSCs form dense fibrotic stroma interact with cancer cells and may also be able to travel throughout the body to inhibit distant metastases. Activated PSCs can influence the formation of a pro-tumorigenic microenvironment by affecting immune regulation, cancer cell stemness, drug resistance induction, angiogenesis stimulation, and increasing metastatic potential [39,40,41].

#### 2.1.3. Endothelial Cells

The vascular endothelium is a thin monolayer of endothelial cells (ECs) that separates circulating blood from tissues, supplies water and nutrients, maintains metabolic homeostasis, transports immune cells, and participates in the formation of new blood vessels [42].

Endothelial heterogeneity and plasticity play an important role in response to stress and pathological conditions. The vascular endothelium an important paracrine and endocrine organ that releases many anti-inflammatory and pro-inflammatory vasoactive molecules, such as nitric oxide (NO), prostacyclin (PGI2), ROS, endothelin-1 (ET-1), tumor necrosis factor-alpha (TNF-α), growth factors, cytokines and arachidonic acid metabolites [43].

Tumor-associated endothelial cells (TECs) show a different, genetic profile, phenotype and function than normal ECs. TECs are characterized by increased expression of the transcription factor c-Myc, CD34, CD61, ALDH, ICAM-1 and VCAM-1, CXCL10, PDL-1 and IDO, and decreased expression of CD105, von Willebrand factor (vWF), MHC-II, CD80 and CD86. They have higher RNA content compared to normal ECs and a highly proliferative phenotype. They exhibit genomic and chromosomal instability, aneuploidy, abnormal multiple centrosomes, deletions, translocations, abnormal epigenetic profile, promoter hypermethylation and histone deacetylation of genes that inhibit angiogenesis and DNA hypomethylation of genes involved in EC growth and germination control. TEC has an irregular shape, wrinkled edges and cytoplasmic protrusions. Intercellular gaps are present, the basement membrane is discontinuous, and smooth muscle coverage is inconsistent. These phenotypic changes lead to functional alterations compared to normal ECs. Since endothelium is in close contact with circulating innate and adaptive immune cells, it mediates interactions between immune cells and cancer cells. TECs can thus promote tumor proliferation, invasion, and resistance to chemotherapy acting as a barrier to tumor-infiltrating immune cells and by transdifferentiating into mesenchymal cells but they can also recruit immune cells and activate their functions [44,45,46,47,48,49,50,51,52,53,54,55,56].

During tumorigenesis, the migration and infiltration of cells into tissues are impaired. Cancer cells secrete cytokines such as VEGF, endothelin 1, EGF-like domain-containing protein 7 (EGFL7), and fibroblast growth factor 2 (FGF2), which downregulate the expression of endothelial selectins, adhesion molecules, and chemokines. This leads to the inhibition of leukocyte migration and the generation of a microenvironment that favors tumor progression. TECs form a selective barrier that allows the infiltration of Treg cells but blocks effector T lymphocytes, dendritic cells, NK cells, and neutrophils, thereby promoting immune tolerance. By increasing the expression of adhesion markers such as the common lymphatic endothelial and vascular endothelial receptor 1 (CLEVER-1), TECs can guide immune cells. By interacting with NK cells, TECs can inhibit their antitumor response. TECs can also interact with tumor-associated macrophages to increase the metastatic potential of cancer cells. This interaction increases endothelial permeability, which promotes adhesion of circulating tumor cells and their migration into tissue [57,58,59,60,61].

Tumor-associated macrophages are characterized by high levels of WNT family gene expression, particularly *WNT7B* and *WNT5A*. *WNT7B* influences angiogenesis and vascular remodeling by increasing VEGF production in TECs. WNT5A stimulates the proliferation and migration of TECs, which facilitates the formation of new blood vessels and contributes to cancer cells migration. VEGFA, IL-10, and prostaglandin E2, produced by cancer cells, collectively induce the upregulation of FasL in TECs. This allows TECs to kill CD8+ effector T cells. TECs express inducible costimulator ligand (ICOSL), which sustains the activation and proliferation of Treg cells and enhances their suppressive function. Interactions between Treg cells and ECs through ICOS/ICOSL interaction increase the expression of the anti-apoptotic protein Bcl-2 on the endothelial surface. ICOSL, through binding to osteopontin (OPN), can induce the migration of TECs and cancer cells. On the other hand, TECs are also involved in the formation of tertiary lymphoid structures (TLS), which contribute to enhancing the immune response. TECs can also influence dendritic cells by regulating their differentiation through the expression of serine/threonine kinase (Stk11). The removal of Stk11 in TECs leads to a significant reduction in the number of mature dendritic cells, which promotes tumor progression. TECs can act as semi-professional antigen-presenting cells and can activate memory T cells. TECs can express MHC class I and II molecules, as well as low levels of costimulatory molecules CD40, CD80, and CD86 required for the activation of naive T cells. TECs have a high degree of plasticity and can change cell fate as needed [3,51,52,62,63,64,65,66,67].

#### 2.1.4. Adipocytes

Adipocytes are characterized by high plasticity in terms of number, size and shape. This plasticity allows them to adapt to systemic or local metabolic changes. They belong to the lipid-rich, highly secretory cells enriched in the long-chain free fatty acids (FFAs) (triacyloglycerols (TAGs) and cholesterol esters enclosed in lipid droplets) and phospholipids, that can be hydrolyzed to form signaling molecules. Outside the bone marrow, adipocytes are comprised of three different cell populations: white adipocytes (comprising white adipose tissue (WAT)), brown adipocytes (brown adipose tissue (BAT)) and beige adipocytes (dispersed within WAT). White adipocytes mainly store lipids in the form of intracellular TAGs and use them as a source of energy for other tissues, while brown adipocytes metabolize FFAs to generate heat. In humans, WAT is widely dispersed across the whole organism and includes subcutaneous (SAT) and visceral (VAT) depots, areas highly predisposed to tumor growth and/or metastasis [68].

Adipocytes are derived from mesenchymal stem cells (MSCs) that are stimulated to differentiate into preadipocytes and finally develop into mature adipocytes. The process is regulated by master regulators (peroxisome proliferator-activated receptor gamma (PPAR-γ) and C/EBPα—CAAT/enhancer-binding protein alpha) [69].

Numerous epidemiological studies highlight the association between obesity and cancer initiation, as well as mortality. This connection is partly due to the ability of adipocytes to exchange signals with cancer cells. Obesity is also considered a poor prognostic factor in many cancers, with potential underlying mechanisms including phenotypic transitions, genomic instability, inflammation, angiogenesis, and/or inhibition of apoptosis [70].

Cancer-associated adipocytes (CAAs), a specific population of adipocytes linked to cancers, are characterized by their ability to differentiate despite nutrient scarcity in the surrounding environment. These adipocytes are located near invasive cancer cells and secrete lipid metabolites, such as free fatty acids (FFAs), which can be utilized by cancer cells as an energy source. This interaction enables cancer cells to shift their metabolism towards lipid dependence. Simultaneously, CAAs release various hormones, adipokines, growth factors, and proteases that facilitate cancer invasion, inflammatory and vascular cell recruitment, and tumor angiogenesis. Conversely, cancer cells contribute to adipocyte remodeling, which involves extracellular matrix (ECM) reorganization, adipose tissue separation, or the incorporation of adipocyte clusters into the cancer lesions. This mechanism of adipocyte integration has been observed in various cancer types, including breast cancer, melanoma, ovarian, prostate, and colorectal cancers. Additionally, metastasis-associated adipocytes are formed during cancer metastasis. In breast and prostate cancers, cancer cells frequently metastasize to bone marrow, which is primarily composed of adipocytes. These adipocytes play a regulatory role in the metastatic process. Cancer cells infiltrating local adipose tissues are associated with poor prognosis, as observed in breast prostate, pancreatic, colorectal, and kidney cancers [71,72,73,74].

Moreover, obesity is closely associated with the development of insulin resistance. Studies have demonstrated a link between insulin resistance and the development of breast, prostate, endometrial, and colorectal cancers. Additionally, the association between obesity and cancer has been confirmed in esophageal cancer, multiple myeloma, and thyroid cancer [71,72,73,74].

As will be elaborated below, adipocytes influence other TME-composing cells, e.g., by releasing adipokines which modulate immune cell activity and contribute to tumor-associated inflammation. Moreover, adipocytes interact with fibroblasts and macrophages, inducing their activation and promoting ECM remodeling. This cross-talk can lead to enhanced cancer cell invasion and metastasis by modifying the mechanical properties of the tumor microenvironment.

#### 2.1.5. Cancer Stem Cells

Cancer stem cells (CSCs) are defined as subpopulations of cells that exhibit a robust capacity for proliferation and a high propensity for self-renewal and differentiation; therefore, they are implicated in the recurrence and metastasis of cancer [75].

During the process of self-renewal, CSCs can undergo either symmetric or asymmetric division, thereby maintaining a stable population of CSCs while contributing to the expansion of the tumor mass. CSCs display the capacity for multi-differentiation, whereby they can differentiate into cancer cells and a range of stromal cells. This enables them to maintain the CSCs microenvironment, that promotes tumor development. Furthermore, evidence indicates that CSCs can differentiate into tumor-associated macrophages (TAMs), cancer-associated fibroblasts (CAFs) or myeloid-derived suppressor cells (MDSCs) under specific circumstances. CSCs exhibit considerable resistance to treatment and are implicated in the maintenance and recurrence of tumors. A principal mechanism of CSC resistance is the phenomenon of cellular plasticity, particularly the capacity of CSCs to enter a quiescent state [76,77,78,79].

#### 2.1.6. Mesenchymal Stem Cells

Mesenchymal stem cells (MSCs) have been the subject of considerable interest over the past two decades. This interest is partly due to their involvement in a range of physiological and pathological processes, including development, tissue repair, organ transplantation, autoimmunity and cancer. MSCs interact with tumor cells, thereby promoting tumor development. They communicate indirectly through the secretion of cytokines, chemokines, and growth factors. Additionally, indirect interactions occur via metabolites, such as PGE2 in lymphoblastic leukemia cells. The direct interactions between MSCs and tumor cells encompass a range of mechanisms, including Notch signaling, the formation of nanotubes, cell fusion, trogocytosis, and the formation of gap junctions, which permit the exchange of second messengers such as cyclic adenosine monophosphate (cAMP), microRNAs (miRNAs), and ions. In their seminal work, Rafii et al. first described trogocytosis, the exchange of plasma membrane fragments between ovarian cancer cells and stromal cells and demonstrated that this process contributes to the development of chemoresistance. The cross-talk between MSCs and tumor cells also involves the activation of pro-inflammatory and pro-survival signaling pathways, including the NF-κB, PI3K/Akt, Wnt, MAPK, and JAK/STAT pathways, which play a role in tumor progression, metastasis formation, and angiogenesis. It has been demonstrated that the activation of the NF-κB and PI3K/AKT signaling pathways promotes immune suppression, EMT and metastasis in breast and gastric cancers. Moreover, within the TME, naive MSCs that exhibit antitumor properties can be reprogrammed by cancer cells into cancer-associated fibroblasts (CAFs), which contribute to tumor progression.

In the tumor microenvironment, MSCs interacts with other TME cells to support tumor progression. For example, MSCs can promote the survival and self-renewal of CSCs, induce the polarization of macrophages towards an immunosuppressive, pro-tumorigenic phenotype, trigger T cell anergy, increase endothelial cell permeability, and stimulate fibroblasts to differentiate into CAFs [80,81,82,83,84,85,86,87].

### 2.2. Immune Components of the TME

#### 2.2.1. T Lymphocytes

Among immune cells, T cells play a crucial role in the anti-cancer immune response. However, during cancer progression, their effector functions become impaired, particularly the CD8+ T cell response to tumor antigens, which contributes to tumor growth. Exhausted T cells, commonly detected in cancers, express specific surface proteins associated with functional impairment [88].

Naïve T cells (prior to antigen recognition) can be classified into several subpopulations, including effector memory T cells (Tem), central memory T cells (Tcm), and tissue-resident memory T cells (Trm). Based on CD4 and CD8 surface expression, T cells are further categorized as cytotoxic (CD8+) or helper (CD4+). Among helper T cells, Th17 cells are particularly relevant in cancer development due to their role in activating neutrophils and monocytes, thereby amplifying inflammation. Trm cells are a key component of tumor-infiltrating lymphocytes (TILs) in patients with solid tumors and are considered as a positive prognostic marker for cancer outcomes [89].

One of the most critical T cell subpopulations in cancer development is regulatory T cells (Tregs), which play a key role in maintaining immune homeostasis. Tregs regulate the function of both CD4+ (helper) and CD8+ (cytotoxic) T cells. Their primary surface markers include CD25, the IL-2 receptor-binding chain, and FoxP3, a transcription factor essential for Treg function, stability, and suppressive capacity. While Tregs are crucial for preventing autoimmune and inflammatory diseases, their accumulation in tumors suppresses anti-cancer immune responses, leading to poorer clinical outcomes. Notably, systemic depletion of Tregs has been shown to enhance tumor rejection, highlighting their potential as therapeutic targets. Tregs secrete various cytokines, including TGF-β, IFN-γ, IL-10, and IL-35, which suppress antigen presentation and reduce CD4+ and CD8+ T cell activity [90,91].

Within the TME, Tregs influence effector cell metabolism by depleting IL-2, a cytokine essential for their growth, thereby contributing to cancer progression. High Treg levels are generally associated with poor prognosis in breast, ovarian, lung, and hepatocellular cancers. However, in colorectal cancer, estrogen receptor-negative breast cancer, squamous esophageal cancer, and ovarian cancer metastases, elevated Treg numbers correlate with better prognosis. This paradox may be attributed to the heterogeneity of Tregs within tumors, the dynamics of chronic inflammation, or the effects of anti-cancer therapies targeting tumor-infiltrating lymphocytes (TILs), which can impact both T cells and Tregs [90,92].

#### 2.2.2. NK Cells

NK cells act independently of antigen recognition. They are able to eliminate cancer cells through direct cytotoxicity. Moreover, they have the abilities to limit tumor growth by releasing proinflammatory cytokines, leukemia inhibitory factor (LIF) and granulocyte-macrophage colony-stimulating factor (GM-SCF). NK cells are not a homogenous cell population and in humans; they can be subdivided based on the surface expression of characteristic markers, adhesion molecule-mediating homotypic adhesion (CD56) and FcyRIIIA, low-affinity receptor for Fc portion of immunoglobulin G (CD16), into several populations. The two most important populations are CD56^bright^ and CD56^dim^ (constituting 10% and 90% of circulating NK, respectively) [93].

Typically, the inhibitory receptors killer cell immunoglobulin-like receptors (KIRs) and immunoglobulin-like transcript 2 (ILT2) are present on CD56^dim^ NK cells. KIRs are involved in both the activation and inhibition of NK cells. During neoplastic transformation, NK cells lose their ability to effectively eliminate cancer cells. This is a consequence of the weakened expression of HLA class I molecules on the surface of cancer cells, which are typically recognized by KIRs [93].

Cytotoxic activity, a major function of NK cells, is higher in CD56^dim^, than in CD56^bright^ NK cells, which is associated with the higher amount of perforin, granzymes and other cytolytic granules. Cytotoxic activity correlates with CD16 expression: the higher the CD16 expression, the more effective the antibody-dependent cellular cytotoxicity (ADCC) process. IL-2 and IL-12 also enhance the ADCC. In contrast, CD56^bright^ NK cells secrete higher amounts of IFN-γ, TNF-α, GM-CSF, IL-10 and IL-13 [94,95,96].

NK cells play an important role in controlling tumor progression directly (interactions with cancer cells) and indirectly (cooperations with other immune cells). Immunopathological studies demonstrate that NK cells infiltrate solid tumors in small quantities, and their presence in the TME is considered to be a good prognostic factor. On the other hand, evidence suggests that the TME can negatively impact on NK cell cytotoxicity. Tumor-associated cells within the TME produce factors that reduce the expression of activating receptors, such as NKp30, NKp44, NKG2D, and tumor necrosis factor-related apoptosis-inducing ligand (TRAIL), on the surface of NK cells. The differentiation and maturation of the NK cells can also be influenced by different cytokines, such as TGF-β that downregulates the effector functions and the expression of the activating KIRs, while simultaneously decreasing mitochondrial metabolism of the cell. Furthermore, the TME regulates NK cell function through the accumulation of adenosine and lactate. Exposure of NK cells to lactic acid diminishes their IFN-γ release upon stimulation. In turn, lactate accumulation in the TME affects the phenotype of cancer cells, enhancing their malignancy [97].

Additionally, NK cells in the TME could receive inhibitory signals (through CD94/NKG2A receptors) which limit their function in head and neck, lung, stomach, colon, liver or pancreas cancer [98,99].

#### 2.2.3. B Cells

B cells originate from bone marrow and express on their surface B cell receptor (BCR) and a costimulatory molecule B220 [100].

Circulating B cells can, after contact with an antigen, transform into plasma cells and release antibodies (most often belonging to the IgG, IgM or IgA class) to the environment. In general, B cells are a part of humoral immune response and in many cases, help T cells and other immune competent cells in the elimination of cancer cells. B cells, similarly, to T cells, can express FoxP3 transcription factor. Bregs are B cells considered to play an important role during the inflammation process that frequently accompanies cancers. They can be generated or activated in response to cytokines (TNF-α, IL-1β, IL-6 or IL-21, GM-CSF) secreted by the immune cells (Th17) as well as by cancer cells and are involved in the generation of immunosuppressive proteins [101].

In the spleen of mice affected by process of inflammation, there is a large population of Bregs releasing IL-10 (so-called B10 cells). Bregs may block NK cells and CD8+ T cells as a result of BCR signaling [102]. At the same time, another Breg population, B35, released IL-35. IL-35 may be generated by both immune cells and cancer cells and is involved in the myeloid-derived suppressor cells (MDSCs) recruitment and cancer progression [101].

In general, Bregs exhibit immunosuppressive properties, with their high number correlating with poor clinical outcomes in cancer patients. This correlation extends to an increased presence of Tregs and MDSCs, which together suppress the anti-cancer immune response [101].

The number of Bregs is regulated by IL-21, a cytokine critical for Igs class switching. It is important, because Bregs modulate the function of the resting immune cells, and their number strictly correlates with a better prognosis in cancer patients. This effect is mainly observed in patients suffering from melanoma, sarcoma, breast cancer, esophageal cancer, non-small-cell lung cancer, colon and biliary tract cancer. In addition, the expression of CD20 on B cells appears to be associated with improved overall survival (OS) in cancer patients. B cells usually interact with dendritic cells and modulate antigen presentation and cytokine release. Human B cells are activated through CD40. On one hand, signaling through CD40 induces a pro-inflammatory effect on the B cells, on the other hand, B cells activation through CD40 induces the development of Bregs. In the TME, they usually display high heterogeneity (occurring as naive, memory, memory activated B cells and even plasmablasts), although the total number of switched memory B cells and plasma cells in the TME is lower than in the peripheral blood [103,104].

B cells prefer to localize in the aggregates and form tertiary lymphoid structures (TLS) that are typical in peritumoral and intratumoral areas. Their presence in the intratumoral zone correlates with the improved overall survival rates in cancer patients. High number of TLS corresponds to better clinical outcomes. At the same time, the exhaustion of B cells (characterized by high expression of CD21 and CD27) correlates with the cancer progression [104,105].

#### 2.2.4. Monocytes

Monocytes belong to the myeloid cell population. They are known as mononuclear phagocytes and are released into the bloodstream. Monocytes are cells that could differentiate into macrophages or dendritic cells (DCs). Their main role is regulation of inflammatory processes; tissue homeostasis supporting, initiating and propagating of host response to the pathogens tissue damaging. In detail, they phagocytize antigen and present it to other immune competent cells [106].

Circulating monocytes are derived from the common myeloid progenitors (CMPs) and in human, they could be classified according to their surface expression of CD14 (a cell co-receptor for lipopolysaccharide (LPS)) and CD16 (the low-affinity IgG receptor) as a classical (CD14highCD16-; 90% of monocytes), intermediate (CD14+CD16+) and non-classical (CD14-CD16high) subpopulations [107,108].

Monocytes can differentiate into tissue macrophages under certain conditions, but this does not happen when their niche is complete. Mature monocytes translocate into the bloodstream where they can circulate for a few days, before migrating into tissues. Eventually, they extravasate or undergo apoptosis. In contrast, in tissues, monocytes can differentiate into DCs or inflammatory macrophages playing an important role in antigen presentation, phagocytosis or immune response modulating [106].

In the context of cancer development, they are described as a factor contributing to cancer growth maintenance. However, not only monocytes but also macrophages and DCs are recruited to primary cancer lesions via chemokines, cytokines, and other molecules released by inflammatory cells and the bone marrow niche. Since the number of monocytes can vary between different cancer types, the proportion of monocytes in the bloodstream, which closely correlates with inflammation status, is considered a prognostic marker for cancer [109].

Monocytes have a major impact on shaping the landscape of the TME. Their plasticity in differentiation (into proinflammatory or anti-inflammatory phenotypes) is crucial to determining whether they support or hinder tumor progression. Pro-cancer activity of classical monocytes involves their differentiation into tumor-associated macrophages, which increases metastatic cell seeding, suppresses cytotoxic functions of T cells and recruitment of Tregs. By stimulation of endothelial cells and fibroblasts, monocytes enhance angiogenesis and ECM remodeling. The anti-cancer function of classical monocytes includes cancer antigen presentation and induction of the T cells cytotoxicity. On the other hand, non-classical monocytes can influence angiogenesis and suppress T cells, simultaneously preventing metastasis and recruiting NK cells to the tumor area [110].

#### 2.2.5. Dendritic Cells

Dendritic cells (DCs) are professional antigen-presenting cells (APCs) known for their ability to present antigens and modulate immune responses. This heterogeneous cell population shares morphological and functional characteristics with monocytes, macrophages, and monocytic myeloid-derived suppressor cells (Mo-MDSCs) due to their common origin. DCs originate from bone marrow stem cells and can be classified into immature (iDCs) and mature (mDCs) subsets. iDCs are primarily found in peripheral tissues, where they specialize in antigen uptake and processing. In contrast, mDCs reside in lymphoid organs, where they interact with antigen-specific T cells to initiate adaptive immune responses [111].

In human and mouse tissues, two major populations of DCs can be distinguished: myeloid DCs (mo-DCs) and plasmacytoid DCs (pDCs). pDCs can be further subdivided according to the strong/weak expression of CD2 into CD2high and CD2low expression, with the former subpopulation being more efficient in inducing allogeneic T cell proliferation [111].

Regulatory DCs (DCregs) play a role in activation of naïve T cells and participate in the antigen presentation. DCregs are resistant to the maturation-inducing signals and release high levels of PD-1, CD95, indoleamine 2,3-dioxygenase (IDO) and several anti-inflammatory cytokines (e.g., TGF-β and IL-10) [112].

In humans, a subset of dendritic cells, known as inflammatory DCs (infDCs), arise from monocytes and play a key role in responses to inflammation and infection. This subpopulation shares a molecular profile with inflammatory macrophages, reflecting their overlapping functions in immune regulation [113].

DCs contribute to both the promotion and suppression of cancer. Key factors involved in DC activation in various carcinomas include STAT3 and NF-κB, which also regulate antigen presentation to T and B cells, highlighting the dual role of DCs in cancer progression and immune defense. Functionally competent mDCs are observed in many types of human cancer (breast, ovarian, melanoma, hepatocellular carcinoma, head and neck squamous carcinoma, non-small-cell lung cancer, cutaneous squamous cell carcinoma, prostate and renal cancer). Immunogenic DCs secrete a set of proinflammatory cytokines (TNF-α, IL-6, 8 and 12), which improve the clinical outcomes in cancer patients [114].

Conversely, the TME suppresses DC function by promoting their functional elimination, inducing tolerogenic and immunosuppressive phenotypes, and preventing direct interactions between DCs and cancer cells. Evidence suggests that TME-derived factors such as VEGF, IL-12, and IFN-γ inhibit the differentiation and maturation of myeloid DCs, further impairing antitumor immunity [115,116].

#### 2.2.6. Myeloid-Derived Suppressor Cells

Myeloid-derived suppressor cells (MDSCs) are a heterogeneous group of cells that negatively affect the antitumor immune response. During tumor progression, the population of MDSCs expands, playing a critical role in promoting tumor immune escape. MDSCs are central to metastasis, tumor progression, and the development of resistance to antitumor therapies. They represent a large group of pathologically activated neutrophils and monocytes with immunosuppressive properties, that could be characterized as granulocytic (PMN-MDSCs) or monocytic MDSCs (mo-MDSCs). Moreover, humans have developed the additional myeloid precursor cells called “early MDSCs”, which are characterized by immunosuppressive activity and represent <5% of the total MDSCs population. In humans, PMN-MDSCs and neutrophils share surface phenotype. Their characteristic marker is lectin-type oxidized LDL receptor 1 (LOX1). In murine models of cancers, PMN-MDSCs show higher expression of genes related to autophagy, cell cycle, G-protein signaling and CREB pathways than normal neutrophils. Moreover, expression of several genes associated with the immune response (TNF-α, IL-1-α, IL-1-β) is increased in PMN-MDSCs cells, in comparison to neutrophils [117,118,119].

Mo-MDSCs can affect the effector function of CD4+ and CD8+ T cells by releasing peroxynitrite, which leads to the loss of T cell responsiveness to specific antigens. In addition, MDSCs participate in the downregulation of CD247, known as the T cell receptor ζ subunit, which is a key subunit in recognizing tumor antigens. Mo-MDSCs secrete nitric oxide (NO) and reactive oxygen species (ROS), which contribute to T cell suppression by downregulating CD247 and interfering with IL-2 receptor signaling. In many cases, MDSCs inhibit the migration of CD8+ T cells into the tumor mass. Additionally, they promote the recruitment of Tregs, further driving cancer progression. In summary, MDSCs accelerate cancer growth by enabling tumor immune escape and counteracting anti-cancer therapies that rely on functional immune responses. Moreover, MDSCs facilitate cancer migration through the secretion of large amounts of metalloproteinases and play a critical role in tumor angiogenesis.

In cancer patients, MDSCs accumulate inside primary and secondary lesions. In acute myeloid leukemias (AMLs), their high numbers strictly correlate with poor prognosis. The number of circulating MDSCs is also related to clinical outcomes in patients undergoing immunotherapy and is a predictive factor of short-term overall survival [39,120,121].

In mouse cancers, MDSCs overexpress a surface marker—CD84 (pan-leukocyte cell surface molecule). They exhibit a T cell-suppressive capacity and increased ROS generation. In humans, CD84 is expressed on the surface of mo-MDSCs and co-expressed with LOX1 on PMN-MDSCs [121].

In the TME, MDSCs increase IL-10 and decrease IL-6, IL-12 and MHC II levels in macrophages, and induce their differentiation into the M2 subtype. Furthermore, MDSCs block anti-cancer function of mature DCs. They interfere with the antigen presentation by DCs and inhibit DCs maturation which impairs anti-cancer immune response. At the same time, PMN-MDSCs and neutrophils are recruited to the premetastatic niches mostly through the CXC2 and CXCR4 cytokines. PMN-MDSCs facilitate cancer cells migration by suppressing immune cells, promoting angiogenesis and inducing ECM remodeling into the new premetastatic niche [121,122].

#### 2.2.7. Neutrophils

Neutrophils play a pivotal role in the body’s defense against tissue damage and also have a significant impact on the progression of cancer. Tumor-associated neutrophils (TANs) contribute to immune suppression, which facilitates tumor growth. The presence of TANs in tumor tissues is associated with worse prognosis in various types of malignant diseases. The quantity and phenotypic and functional characteristics of tumor-infiltrating neutrophils may serve as biomarkers for predicting the response to chemotherapy or immunotherapy and overall survival in cancer patients. Despite the incomplete understanding of the interactions between neutrophils and either cancerous or non-malignant cells within the TME, it has been reported that neutrophils are involved in complex bidirectional interactions with these cells. Prognostic relevance of neutrophils is usually based on calculations of the neutrophil-to-lymphocyte ratio (NLR). High NLR values are associated with worse prognosis for cancer patients. It is of particular importance to note that the number and localization of neutrophils within the TME appear to influence their activation and functions. The number and localization of neutrophils within the TME significantly influence their activation and functions, with intratumoral, peritumoral, and stromal neutrophils showing different prognostic values for overall survival. Neutrophils exhibit phenotypic plasticity, modulated by environmental conditions, and their activation is shaped by the surrounding microenvironment. TANs can promote or suppress tumors depending on the evolving tumor microenvironment, reflecting the dynamic complexity of TAN interactions within the TME [123,124,125,126,127,128,129,130,131].

#### 2.2.8. Eosinophils

In humans, eosinophils originate from CD34+CD117+ pluripotent hematopoietic stem cells from the bone marrow, where they undergo maturation before entering the circulation. They constitute approximately 1% of leukocytes in the bloodstream. Most human and murine experimental studies indicate that eosinophils exert antitumor effects. In malignancies, the presence of eosinophils at the tumor site or in peripheral blood is associated with a favorable prognosis for most cancers. Moreover, blood eosinophilia is linked to a favorable response to immunotherapy and is generally associated with longer survival in patients with advanced melanoma. However, it is an unfavorable prognostic factor in T cell leukemia/lymphoma. Eosinophils produce several chemokines (CCL5, CCL9, CXCL10) that are essential for the attraction of CD8+ T cells in the TME. In addition, eosinophils facilitate M1 macrophages to secrete IFN-γ and TNF-α. Consequently, M1 macrophages promote Th1 response, establishing a positive feedback loop in the antitumor response. Furthermore, eosinophils contribute to the normalization of tumor vasculature, thus promoting tumor rejection. IL-33 administration in melanoma-bearing mice delays tumor growth by promoting intratumoral accumulation and activation of eosinophils. Activated eosinophils support antitumor immunity by directly killing melanoma cells and recruiting CD8+ T cells to the tumor site. IL-33 also recruits eosinophils to the lung via ST2, preventing pulmonary metastasis after intravenous melanoma cell injection [132,133,134,135,136,137].

#### 2.2.9. Basophils

Basophils constitute less than one percent of human peripheral leukocytes. They exhibit certain similarities with mast cells, notably the presence of basophilic granules within the cytoplasm. A study on the role of basophils in ovarian cancer patients revealed a positive correlation between the proportion of circulating basophils and overall survival in these patients. Moreover, protein and gene expression analysis revealed the presence of both resting basophils characterized by CCR3, CD123, FcεRI markers and activated basophils (CD63, CD203c markers) within ovarian tumors.

In a mouse model of melanoma, depletion of regulatory T cells (Tregs) was associated with tumor infiltration by basophils and CD8+ T cells, which resulted in melanoma rejection. Basophils facilitated the infiltration of CD8+ lymphocytes into the tumor through the production of chemokines CCL3 and CCL4. In a large cohort of patients with pancreatic ductal adenocarcinoma (PDAC), basophils expressing interleukin-4 (IL-4) were identified in the tumor-draining lymph nodes (TDLN; the main sites of antitumor immunity development). The presence of basophils in the TDLN was identified as an independent, negative prognostic biomarker for patient survival following surgical intervention. Furthermore, potential involvement of basophils in an orthotopic model of pancreatic cancer was studied using basophil-deficient Mcpt8-Cre mice. After implantation, tumors were identified in 80% of mice, whereas no such tumors were observed in basophil-deficient mice [138,139,140,141].

#### 2.2.10. Macrophages

Macrophages are innate immune cells that play a pivotal role in maintaining tissue homeostasis, the clearance of superfluous cells, and the inflammatory response to infection. In cancer, macrophages exhibit a range of functions, from antitumor activity in the early stages of cancer progression to a tumor-promoting role in advanced cancer. The heterogeneity of macrophages has historically been simplified into a dichotomous classification, comprising the classically activated M1 subtype and the alternatively activated M2 subtype. M1 macrophages exhibit a pro-inflammatory phenotype with pathogen-killing abilities, and produce pro-inflammatory cytokines, including TNF-α, IL-1β, IL-12 and IL-23. Additionally, they secrete ROS and exhibit augmented antigen-presenting capabilities. M2 macrophages display augmented phagocytic activity, elevated expression of scavenger receptors, augmented activity of the arginase pathway, and the secretion of IL-10, transforming growth factor-beta (TGF-β), and vascular endothelial growth factor (VEGF). Consequently, M2 macrophages have anti-inflammatory effects and play a crucial role in the promotion of tissue remodeling, neoangiogenesis, and tumor progression. They can inhibit cytotoxic T cells and NK cells. They also interact with fibroblasts, supporting their activation and contributing to ECM remodeling [67,132,142,143].

A recent review consolidating the results of 300 studies showed a clear prognostic correlation between various solid tumors and their infiltration by M1 or M2 macrophage subtypes. The presence of the M2 subtype was found to be associated with a poor prognosis for patients, whereas the presence of M1 macrophages was associated with a favorable prognosis [144].

As evidenced by the above description, the various cells comprising the tumor microenvironment form a complex network of interactions with each other and with cancer cells, dynamically influencing tumor progression. Through these interactions, they modulate key processes such as proliferation, immune evasion, and metastasis.

In the following chapters, the contributions of individual cells within the tumor microenvironment to the development of various types of cancer will be described. Due to the limited literature available on certain cell types, and in order to avoid excessive fragmentation of this paper into short subsections, information on different cell types has sometimes been combined into a single chapter where possible, such as “Non-Immune Cells” or “Monocytes and Related Cells”.

## 3. The Role of TME Cells in Tumor Development Across Cancer Types

### 3.1. Head and Neck Cancer

Head and neck cancer (HNC) is the seventh most common cancer in the world. Nearly 90% of HNCs consist of squamous cell carcinoma, which arises from the epithelium of the oral cavity, larynx, and pharynx. Tobacco use, alcohol consumption, and infections with HPV-16, and to a lesser extent HPV-18, are considered the most important factors influencing HNC development, especially in the case of laryngeal cancer. The gene encoding tumor protein p53 (*TP53*) is mutated in 60–80% of HNC patients, and they have relatively poor clinical outcomes. In HPV-positive tumors, wild-type TP53 is usually present. The TME in the HNC is shaped by several clinical features: tumor stage, mutational burden, tobacco alcohol abuse and HPV status [145,146].

#### 3.1.1. CAFs in Head and Neck Cancer

In head and neck squamous cell carcinoma (HNSCC), three main CAFs subtypes are myCAFs, iCAFs, and apCAFs. The sources of CAFs in HNSCC include normal fibroblasts, endothelial cells, epithelial cells, adipocytes, pericytes, and MSCs [147,148].

CAFs promote the development of HNSCC by influencing cancer cell proliferation, angiogenesis, migration, invasion, and the development of chemoresistance. CAFs-derived IL-6 may promote tumor proliferation by regulating osteopontin expression in a STAT3-dependent manner. Aging CAFs promote a more aggressive phenotype in oral cancer by producing active MMP2, disrupting epithelial adhesion, and inducing keratinocyte invasion. CAFs induce angiogenesis in HNSCC through the production of prostaglandin E2 (PGE2) via COX-2. CAFs can influence the formation of new lymphatic vessels by altering their permeability and causing changes in gene expression within lymphatic vessels. CAFs regulate the immunosuppressive tumor microenvironment in HNSCC by supporting the tumor-promoting phenotype of TAMs. AKT3 expression in CAFs influences immunosuppression in HNSCC by reducing T cell proliferation, promotes cancer cell proliferation and migration, and is associated with poor prognosis. CAFs-derived WNT2 inhibits the antitumor response of T cells dependent on dendritic cells via the SOCS3/p-JAK2/p-STAT3 signaling pathway and promote the survival of HPV-negative HNSCC after cisplatin treatment, as well as the survival of both HPV-positive and negative cells during cetuximab and mTOR inhibitor treatments. MMP1 produced by CAFs also contributes to chemoresistance to cetuximab. Higher TGF-β pathway activity in CAFs is also associated with HNSCC resistance to cetuximab. CAFs in HNSCC may deliver miR-196a into tumor cells via exosomes, thereby causing cisplatin resistance. CAFs induce resistance to immunotherapy by selectively depleting CD8+ T cells from the tumor microenvironment [79,149,150,151,152,153,154,155,156,157,158,159].

CAFs promote proliferation by increasing phosphoinositide-dependent protein kinase-1 (PDK1) and Akt phosphorylation and decreasing cRAF and PTEN in oral squamous cell carcinoma (OSCC). CAFs derived from OSCC show significantly higher levels of integrin beta 2 (ITGB2), which controls the PI3K/AKT/mTOR pathway, increasing CAF glycolytic activity. Lactate secreted by CAFs with ITGB2 overexpression is then taken up by cancer cells, promoting OSCC growth. Increased ITGB2 expression is associated with poor prognosis [160,161].

#### 3.1.2. Adipocytes in Head and Neck Cancer

Adipocytes and cancer cells in HNC patients constantly interact. Adipocytes facilitate cancer cell uptake of FFAs and glycerol, fueling oxidative phosphorylation and promoting invasion and metastasis. Beyond metabolic effects, adipocytes secrete adipokines (leptins, adiponectin, IL-6), which enhance cancer invasion in melanoma patients. In HNC patients, low neck adipose tissue (NAT) density before or after treatment correlates with worse prognosis and shorter survival, while higher NAT levels associate with better outcomes. This protective effect likely stems from adipose tissue mitigating radiotherapy side effects and nutritional disturbances [162,163,164].

#### 3.1.3. CSCs in Head and Neck Cancer

A body of evidence supports the presence of CSCs in HNC, including both sits squamous and non-squamous subtypes. CD44 (a key CSC marker)-positive cells purified from HNSCC retain the tumor heterogeneity observed in the original tumor. Additionally, in murine models, CD44+ cells exhibit enhanced migratory and invasive capabilities, contributing to a higher propensity for lung metastases [165,166].

#### 3.1.4. T Cells in Head and Neck Cancer

In HNC, T cells comprise four subpopulations: Tregs, CD4+ Tconv (conventional), CD8+, and CD8+ Texhausted. The presence of tertiary lymphoid structures (TLS) in HNC patients plays a role in predicting immunotherapy response, similar to their function in adult sarcomas. In HNC, infiltration by lymphocytes (CD4+, CD8+, B cells) and myeloid dendritic cells (neutrophils, macrophages, monocytes, eosinophils, dendritic cells) plays a critical role. Cancer development occurs through immune evasion, regardless of phenotype or molecular profile. For instance, HNSCC cells evade T cell elimination by downregulating HLA molecule expression [167].

In Oropharyngeal Squamous Cell Carcinoma (OPSCC) patients, the majority of TILs within the tumor are T CD8+ cells, although T CD4+ cells predominate in some patients. Interestingly, a higher percentage of CD8+T cells corresponds to better clinical outcomes in HNC patients, and the ratio of CD4+:CD8+ is higher in advanced stages of tumors. Furthermore, higher levels of the CD4+ and CD8+ TILs correlate with improved OS and relapse-free survival [168].

T cells in HNC patients produce less IFN-γ after stimulation with antigens in comparison to unstimulated controls. Moreover, dysfunctional TILs reveal diminished cytokine secretion and proliferation ability and lack of cytotoxic activity. Dysfunctional TILs in HNCs are characterized by increased expression of PD-1, LAG-3, TIM-3, CTLA-4 and 2B4. PD-1 is a marker of T cell exhaustion upregulated in CD4+ and CD8+ TILs. In HNCs, almost 70–80% of patients have PD-L1-positive tumor cells. Interaction of T cells’ PD-1 with PD-L1 leads to inhibition of T cells and their apoptosis [169].

On the other hand, dysfunction of CD8+ T cells is correlated with high levels of Tregs and Th17, whose proliferation is driven by increased levels of IL-23 and IL-6 released by HNSCC cells. In general, Tregs consist of <5% of CD4+T cells in peripheral blood and are characterized by CD4+CD25+Foxp3+ phenotype that regulates immunotolerance towards neoplastic cells. Peripheral Tregs are recruited to the TME, where TGF-β (secreted, e.g., by DCs) induces their differentiation and immunosuppression. Increased levels of the TGF-β are typical for latter phases of HNCs. However, it was demonstrated that high levels of the Tregs are associated with the longer DFS and higher OS rates. On the other hand, decreased expression of the HLA molecules influences tumor antigen presentation and leads to immune response evasion in almost 50% of patients with HNC [170].

#### 3.1.5. NK Cells in Head and Neck Cancer

In HNSCC patients, NK cell infiltrations are predominantly composed of the CD56^dim^ subset. These infiltrations are larger in HPV+ (human papillomavirus-positive) patients than in those without HPV-related cancer. Increased CD56^dim^ NK cell infiltration correlates with better prognosis and longer survival in both groups. Compared to circulating NK cells, infiltrating NK cells in HNSCC more frequently express the inhibitory receptor NKG2A on their surface, while KIR expression is less common.

In addition to mature NK cells, the TME also contains immature CD56^bright^CD16^dim^/-NK cells lacking CD57 expression, which limits the anti-cancer immune response [171].

#### 3.1.6. B Cells in Head and Neck Cancer

In head and neck squamous cell carcinoma, B cells form TILs that, along with plasma cells, positively impact overall survival, though outcomes vary among patients. HNSCC is dominated by B cells expressing high levels of CD86, a marker of activated B cells that influences their antigen-presenting ability. Additionally, tumor tissues show increased memory B cells and plasmablasts compared to peripheral blood. A high number of CD20+ B cell infiltrations correlates with better prognosis in HNSCC patients [172].

HPV-positive HNSCC patients exhibit a stronger B cell-mediated immune response, as HPV infection generates specific cancer-associated antigens. The broader B cell repertoire against these antigens enhances immune response and correlates with improved prognosis. Additionally, the cooperation between B cells and CD8+ T cells contributes to more effective tumor elimination in HPV-positive patients [173].

Regulatory B cells are also present in HNSCC, though their prognostic significance remains unclear. The TME in HNSCC is dominated by B cells expressing CD24+CD38+CD19+, a subtype that promotes immunosuppression by inhibiting effector immune cells. Breg-derived IL-10 facilitates Th1 differentiation into Tregs, which correlates with worse prognosis [174].

At the same time, opposing findings indicate that CD24+CD38+ Bregs from non-sentinel lymph nodes in HNSCC patients are associated with limited cancer infiltration and low histologic grade, both positive prognostic markers. This contradiction highlights prognostic variability in this patient group depending on individual factors [172].

#### 3.1.7. Monocytes and Related Cells in Head and Neck Cancer

In the squamous cell carcinoma of the head and neck, the total number of intermediate monocytes in peripheral blood is significantly lower than in healthy individuals. Unfortunately, there are no studies demonstrating the direct influence of monocytes on the development of head and neck cancer. Similarly, the number of the mDCs in HNC correlates with advanced stages of the disease and shorter OS [175].

In HNC patients, MDSCs circulate in peripheral blood and enter into the draining lymph nodes. MDSCs are CD11b+, whereas the immature mo-MDSCs are characterized by the Ly6C^high^ and Ly6G- phenotype, and both cell types may transform into tumor-associated macrophages under the STAT3 signaling. The prevalence of this subpopulation negatively correlates with response to chemotherapy in HNC patients. Peripheral MDSCs suppress antigen-specific CD8+ T cells in peripheral blood, while MDSCs from the TME suppress antigen-specific immune response in tumor mass They are also involved in the T cell function modulation, and their number strictly correlates with the risk of recurrence [176].

#### 3.1.8. Neutrophils in Head and Neck Cancer

Neutrophil-to-lymphocyte ratio is a reliable predictor of overall survival and mortality associated with HNSCC. In the advanced and late stages of the disease, a considerable number of infiltrating neutrophils are present in HNSCC samples. Lymphotoxin β4 (LTB4) recruits additional neutrophils, sustaining tumoricidal activity. By attracting fresh neutrophils to replace exhausted effector cells, LTB4 helps maintain a favorable effector-to-tumor cell ratio. Furthermore, it recruits other immune cell types, including T cells. CD177+ neutrophils have been demonstrated to exert antitumor effects, including the prevention of disease progression and the improvement of patient survival. CD177+ neutrophils require a diminished activation threshold and exhibit a distinctive cytokine profile in comparison to their CD177- counterparts [177,178,179,180,181].

#### 3.1.9. Macrophages in Head and Neck Cancer

A 2018 study analyzed macrophage subpopulations in HNSCC patients by assessing the M1 macrophage marker CD80 and the M2 macrophage marker CD163. The results showed a significant increase in CD163+ tumor-associated macrophages, which inversely correlated with relapse-free survival (RFS), progression-free survival (PFS), and OS. These findings suggest that targeting macrophages, particularly the M2 phenotype, may improve prognosis and treatment outcomes in head and neck cancer patients. Gao et al. identified TAM biomarkers and EMT-related proteins, revealing a positive correlation between EMT protein expression and M2 macrophage biomarkers in HNSCC tissues. Their study suggests that M2-type TAMs promote the EMT process by activating the EGFR/ERK1/2 signaling pathway, thereby enhancing tumor growth [182,183].

### 3.2. Glioma

Gliomas are primary central nervous system (CNS) cancers with multiple histologic types. They are derived from glial cells or from stem cells that exhibit glial properties upon transformation. Traditionally, glioma in adults can be divided into astrocytoma, oligodendroglioma, and ependymoma. Oligodendroglioma is associated with a better prognosis and higher chemosensitivity than astrocytoma [184].

Astrocytoma is derived from astrocytes. Glial fibrillary acidic protein (GFAP) is a main constituent of their fibrils and a hallmark of differentiation. Oligodendroglioma is derived from oligodendrocytes, which are characterized by the overexpression of Nkx2.2, a homeodomain protein, and by the oligodendrocyte lineage-specific basic helix-loop-helix 2 (Olig2) transcription factor.

In turn, ependymoma is derived from ependymal cells that line the ventricles and the spinal cord and make cerebrospinal fluid. Ependymoma can spread to other parts of CNS in cerebrospinal fluid but spread outside the CNS is very rare. Ependymoma is more common in young children than in adults [185,186].

Approximately half of all diagnosed gliomas in adults are glioblastomas (GBMs), and only approximately 5% of them are hereditary. Most often, they develop spontaneously for unknown reasons. The only confirmed risk factor is ionizing radiation. Patients with gliomas have poor clinical outcomes [187].

The role of the TME in glioma development is primarily associated with the release of cytokines such as IL-6, platelet-derived growth factor (PDGF), VEGF, and epidermal growth factor receptor (EGFR). Hypoxia plays a crucial role in the glioma microenvironment, as HIF overexpression promotes a more aggressive, treatment-resistant phenotype. Additionally, glioma patients often exhibit systemic immunodeficiency, with prognosis linked to immune cell function. Notably, glioma treatment decisions depend on TME composition, as patients with the same tumor grade but different TME composition may require distinct therapeutic approaches [188,189].

#### 3.2.1. Non-Immune Cells in Glioma

**Figure 4:**
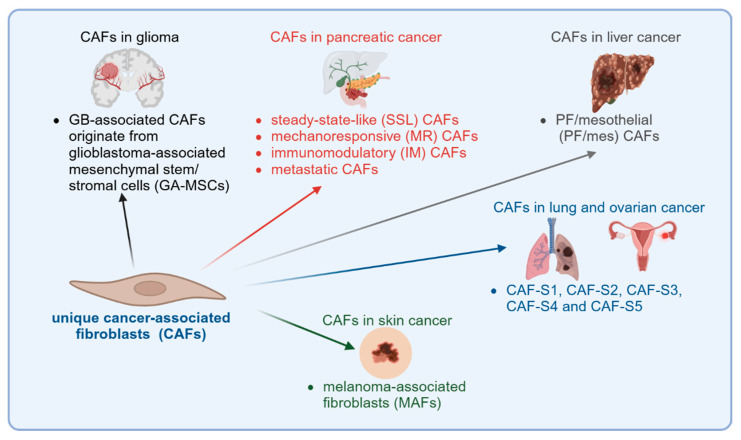
Cancer-associated fibroblasts (CAFs) of various origins specific to a given cancer type.

CAFs are important cells in the glioblastoma TME. They presumably originate from glioblastoma-associated mesenchymal stem/stromal cells (GA-MSCs) (Figure 4). Alternative origins of CAFs are not known. CAFs in glioblastoma are characterized by expression of α-SMA, COL1A1, FAP, TNC, PDGFR-α, PDGFR-β, PDPN, VIM (vimentin), LOX, CAV1 and S100A4. However, there are different subtypes of CAFs that express various markers. Two different subtypes of CAFs have been described: one subtype expressing α-SMA and PDGFR-β, and another subtype expressing FAP.

CAFs in glioblastoma are able to enhance the proliferation of glioblastoma stem cells by expressing osteopontin and hepatocyte growth factor, as well as drive macrophage polarization towards the tumor-promoting type, mediated by fibronectin produced by CAFs. Fibronectin, an important matrix protein, also promotes cancer cell survival and proliferation. CAFs also play an important role in increased angiogenesis in glioblastoma [152,190,191,192,193].

The direct link between obesity and glioma development remains unclear. However, prospective studies suggest an association between weight and glioma risk. Notably, patients with abdominal obesity have a higher glioma risk, with a positive correlation between leptin levels and tumor development [194].

These fibroblasts are distinct from the common fibroblast subtypes found in other malignancies, highlighting their role in the tumor microenvironment of the respective cancers.

CSCs play a crucial role in glioma development and progression, including GBM. Their interaction with the TME influences tumor invasiveness and heterogeneity. Glioma CSCs reside in specific TME niches, shielding them from therapy. They can survive and proliferate in hypoxic, nutrient-poor conditions, making them difficult to eradicate. Additionally, CSCs contribute to disease relapse by reinitiating tumor growth after cessation of therapy [195].

#### 3.2.2. Immune Cells in Glioma

In glioma, Tregs play a key role in tumor mass infiltration, comprising 60% of total T cells and being associated with poor prognosis. This may be due to TME-associated T cell immunosuppression, as Treg depletion has been shown to restore CD4+ T cells and improve prognosis. Tregs secrete high levels of IL-10 and TGF-β, contributing to poor prognosis. Higher IL-10 and TGF-β levels are associated with worse outcomes in glioma [196,197,198].

In glioblastoma, NK cells display CD56^dim^CD16- phenotype and they represent only approximately 2% of the total cells. Their cytotoxic activity is disrupted, partly by glioblastoma cells. Glioblastoma cells express high levels of HLA class I molecules, which interact with inhibitory receptors on NK cells, impairing NK cell-mediated cytotoxicity. Additionally, glioblastoma cells exhibit altered MIC-A and MIC-B expression affecting NKG2D and CD27 activation on NK cells and leading to cytotoxic function inhibition. Glioblastoma cell lines also express lectin-like transcript-1, a ligand for CD161, further suppressing NK cell function. NK cell cytotoxicity can be modulated by immunosuppressive cytokines released by Tregs and MDSCs within the tumor mass, as well as by pro-inflammatory cytokines (IL-12, IL-15, IL-18, IL-21) and glioblastoma-derived TGF-β [72,199,200,201,202].

The role of B cells in brain tumor development remains unclear. While they infiltrate meningioma and glioma, they constitute only a small fraction of the TME. B cells can function as antigen-presenting cells and contribute to CD4+ T cell-dependent induction of CD8+ memory T cells. In a murine glioblastoma model, they activate T cell-mediated antitumor immunity and tumor regression. However, B cells in glioblastoma may also suppress immune responses and promote M2 TAM differentiation, facilitating tumor progression. Additionally, Bregs contribute to glioma growth by releasing TGF-β in response to placenta growth factor, which induces B cell-to-Breg conversion [203,204].

Normal monocytes (CD14+), upon exposure to glioblastoma-specific antigens, acquire an MDSC phenotype, secreting IL-10 and TGF-β, which induce apoptosis in activated lymphocytes. This process is linked to unfavorable prognosis and poor clinical outcomes, as IL-10 and TGF-β contribute to T cell exhaustion [205].

In normal conditions, DCs are absent in the brain parenchyma; however, during cancer development, they move into the brain through endothelial venules or lymphatic vessels. In brain tumors, DCs are hypothesized to present cancer antigens within the brain or in surrounding lymphoid structures, to sensitize T cells to the antigens. In glioma patients, fibrinogen-like protein 2 (FGL2) produced by cancer cells interacts with the GM-SCF signaling, which influences differentiation of CD103+cDC1s and limits CD8+ T cells immune response. In glioma patients, cancer cell-derived PGE2 enhances IL-10 secretion by DCs, which subsequently promotes Treg activity. The glioma TME may also drive overexpression of nuclear factor erythroid 2-related factor 2 (Nrf2), a redox-sensitive transcription factor in DCs, inhibiting their maturation and reducing T cell activation. Notably, inhibiting Nrf2 restores DC maturation in glioma-conditioned medium [206].

In glioblastomas, MDSCs exhibit functional overlap with TAMs (M2 macrophages) in their immunosuppressive activity, as they share characteristic features of TAMs. In the peripheral blood in glioblastoma patients the CD33+ MDSCs were found in higher levels than in healthy individuals [205].

Glioma cells recruit MDSCs to the TME via chemokine signaling. Notably, recruited MDSCs exhibit high lysine demethylase 6B (KDM6B) expression, and in a mouse model, KDM6B knockdown led to strong proinflammatory activity and improved prognosis. Systemic and intratumoral MDSC levels increase with tumor progression, correlating with glioma grade, and their rise during recurrence is an unfavorable prognostic factor [207].

In human glioma, both PMN-MDSCs and Mo-MDSCs are detected in peripheral blood and tumors, though their numbers are higher in blood. Interestingly, MDSC accumulation appears sex related: elevated PMN-MDSC levels in females predict worse outcomes, while Mo-MDSCs predominate in males and are also linked to poor prognosis [206]. Recent studies suggest that neutrophils play a key role in glioblastoma progression. Elevated circulating neutrophil levels correlate with increased tumor infiltration and poorer prognosis [208].

TAMs are the most abundant immune cells in glioblastoma, comprising up to 50% of the tumor mass. Recent studies highlight their key role in promoting tumor progression and immunosuppression in GBM. As a result, the TME may contribute to GBM progression and resistance to chemotherapy and immunotherapy [209,210,211,212].

### 3.3. Thyroid Cancer

Thyroid cancers are common endocrine malignancies with low mortality but high recurrence and persistence rates. Risk factors for thyroid cancer (TC) fall into high-risk, low-risk, and unclear categories. High-risk factors include head and neck radiation exposure, genetic mutations, and hereditary conditions. Lower-risk factors include I-131 thyroid imaging, iodine deficiency, elevated serum TSH, autoimmune processes, thyroid nodules, and environmental exposures (e.g., nitrates, certain vegetables, asbestos, benzene, formaldehyde, pesticides, and polybrominated diphenyl ethers (PBDEs)). Poor diet and a sedentary lifestyle are also linked to lower risk. The influence of blood estrogen levels remains unclear [213].

There are several histological types and subtypes of TC differing in cellular origin, characteristic features, and prognosis. TC is classified into those derived from follicular thyroid cells (including PTC—papillary TC, FTC—follicular TC, PDTC—poorly differentiated TC, and ATC—anaplastic TC) and those derived from parafollicular C cells. PTC and FTC are referred to as differentiated TC (DTC) and constitute the majority, while ATC is the rarest but most aggressive form. Medullary TC (MTC), which is derived from parafollicular C cells, accounts for only a small percentage of thyroid cancers. Poorly differentiated thyroid cancer (PDTC) and anaplastic thyroid cancer (ATC) are rare cancers associated with aggressive behavior and short survival times. PDTC represents approximately 5%, while ATC accounts for 1% of all cases [214].

As in other cancers, the TME in TC influences the biology of cancer, its growth and progression. Thyroid cancer is characterized by a large amount of fibrous stroma and may be associated with CTLs (chronic lymphocytic thyroiditis) [215].

#### 3.3.1. CAFs in Thyroid Cancer

In TC, the primary sources of CAFs are normal fibroblasts and MSCs. The two main subpopulations of CAFs found there are myCAFs and iCAFs. In TC, CAFs promote cancer cell proliferation, migration, invasion, and metastasis formation [216,217,218].

CAFs increase the expression of vimentin and decrease the expression of E-cadherin in thyroid cancer cells, and they also secrete the Sonic-Hedgehog ligand, which induces migration [216,217,218].

CAFs synthesize and secrete CXCL12, which is a potent inflammatory factor that promotes proliferation, migration, and invasion of TC cells. CXCL12 binds to CXCR4 and CXCR7 proteins on thyroid cancer cells and activates several signaling pathways such as mTOR, ERK1/2, SAPK/JNK, Akt, p38, and BTK. Ursolic acid (UA), a natural triterpenoid with anti-inflammatory properties, inhibits the secretion of CXCL12 in CAFs, and also reduces the expression of CXCR4 and CXCR7, thereby exerting strong anti-cancer effects. Therefore, therapies targeting the CXCL12/CXCR4/CXCR7 axis could be a promising and effective therapeutic strategy for thyroid cancer [219].

CAFs in cooperation with TC cells increase ECM stiffness. CAFs contribute to this by abnormal and excessive deposition of COL-1 fibers, whereas TC cells, through the expression of LOX, cross-link collagen fibers originating from fibroblasts. These collagen fibers delineate pathways through which TC cells can migrate from the primary tumor mass to local and distant secondary sites. COL-1 and LOX have been identified as markers of aggressiveness in human TC and are associated with a poor prognosis. Therefore, the ability of CAFs to remodel the ECM could be a promising therapeutic target in thyroid cancer [220,221].

CAFs facilitate TC progression by immunosuppression. CAFs increase the expression of various regulators of immune checkpoints such as CTLA-4, PDL-1/2, and IDO-1, thereby enabling immune escape and tumor growth. A high level of CAFs in TC patients is positively correlated with increased expression of CD274, PDCD1LG2, CD86, CD80, CTLA-4, EGF-like module-containing mucin-like hormone receptor-like 1 (EMR1), CSF1R, CD163, and integrin alpha M (ITGAM), indicating the role of CAFs in modulating immune cell functions. The exact mechanisms underlying these processes in TC have not yet been fully elucidated [222,223].

CAFs are also responsible for the metabolic reprogramming of TC cells [217,218].

#### 3.3.2. Other Non-Immune Cells in Thyroid Cancer

Obesity and adipocyte count positively correlate with papillary, follicular and anaplastic TC, whereas the correlation with medullary thyroid cancer is negative [224].

The origin of thyroid cancer stem cells (TCSCs) remains incompletely understood. It remains unclear whether TCSCs originate from precursors or mature cells, and whether TC cells are the result of genetic mutations or epigenetic alterations occurring in thyroid stem cells (TSCs). Both TCSCs and TSCs generate thyrospheres in culture. However, only thyrospheres derived from normal thyroid stem cells differentiate when plated in adhesion in the presence of thyroid-stimulating hormone (TSH). The presence of stemness markers, including CD133, CD44, Oct-4, Sox-2, and Nanog, is observed in both normal and cancer thyrospheres. Thyroid differentiation markers, including thyroperoxidase (TPO), thyroglobulin (Tg), and thyroid-stimulating hormone receptor (TSH-R), are expressed at low levels in both cell types. In contrast, insulin receptors (IR-A, IR-B), insulin-like growth factors (IGF-I, IGF-II), and the IGF receptor (IGF-IR) are more highly expressed in TCSCs than in differentiated cells. These findings suggest a link between insulin resistance and increased TC susceptibility, highlighting insulin and IGFs as potential therapeutic targets [225,226].

#### 3.3.3. T Cells in Thyroid Cancer

In thyroid cancer, Tregs are highly abundant in PTC tissue, correlating with extrathyroidal extension and lymph node metastasis. Their presence is linked to papillary microcarcinoma aggressiveness, as immune suppression depends on Treg differentiation promoted by IDO-1 overexpression. In PTC, Tregs mainly arise from Th1 cells via DC-mediated ICOS signaling. The total Treg count correlates with PTC and FTC aggressiveness, while tumor size inversely correlates with FoxP3 expression [227,228].

A distinct T cell subtype, CD3+CD4-CD8- (double-negative or DN T cells), represents the dominant T cell population in PTC. Additionally, Th17 cells are abundant in differentiated thyroid cancer (DTC). In DTC, CD8+ T cell infiltration correlates with better prognosis, but high levels, combined with COX-2 expression, are linked to recurrence [215].

#### 3.3.4. NK Cells in Thyroid Cancer

Thyroid-stimulating hormone binds to peripheral blood mononuclear cells (PBMCs), including monocytes, NK cells, and activated B cells. In older individuals, NK cell cytotoxicity declines but can be restored by triiodothyronine (T3). Notably, in thyroidectomy patients with early-stage thyroid cancer, anti-tetraiodothyronine treatment increases serum IL-18 levels and NK cell activation. Thyroid hormones also influence NK cell cytokine secretion, with T3 inducing IL-2R expression in PBMCs [229,230].

In PTC patients, NK cell infiltration is more extensive in early stages, with higher cancer-infiltrating NK cell numbers compared to goiters and healthy tissues. CD56^bright^ NK cell levels are elevated in PTC regardless of stage, whereas NK cell cytotoxicity correlates with disease stage. NK cell recruitment to thyroid cancer tissue is mediated by the CXCL10/CXCR3 axis [231,232,233].

#### 3.3.5. Monocyte-Related Cells in Thyroid Cancer

In thyroid cancers, DCs infiltrating the tumor mass exhibit an immature phenotype and fail to properly activate T cells due to low costimulatory molecule expression. High levels of IL-10 and TGF-β released by DCs further suppress T cell activity. DCs are generally scarce in thyroid cancers, except in PTC [234].

In PTC, DC numbers, particularly CD1a+ DCs, are elevated and correlate with improved survival. However, S100+ DCs do not influence long-term survival and instead promote Th1-to-Treg transformation, which is detrimental to patients [235,236].

MDSC levels are elevated in TC patients compared to those with benign thyroid disorders, with circulating MDSCs correlating with differentiated thyroid cancer aggressiveness. However, no significant link to pathogenesis has been observed. Peripheral blood MDSCs are higher in ATC patients and strongly correlate with IL-10 serum levels, indicating their role in systemic immunosuppression. In contrast, intratumoral MDSC levels do not associate with clinical outcomes in TC patients [227].

#### 3.3.6. Neutrophils in Thyroid Cancer

Neutrophils are activated in TC, with their levels correlating with increased tumor size. As escort cells for circulating tumor cells, they promote cell cycle progression and enhance metastatic potential. Tumor-activated neutrophils release proinflammatory factors (IL-6, IL-8, IL-1β, TNF-α), which activate the ERK pathway and epithelial–mesenchymal transition, driving TC cell migration and invasion. It has been shown that co-incubation with neutrophils induces cancer cell proliferation in a dose-dependent manner via elastase and cyclooxygenase-2 secretion (COX2). Inhibiting neutrophil elastase or COX-2, either in vitro or via gene knockout, significantly reduces tumor growth [237,238,239,240].

#### 3.3.7. Macrophages in Thyroid Cancer

An increase in TAMs is observed in high-grade TC and is associated with an increased likelihood of invasive cancer and a reduction in cancer-related survival. It is currently believed that TAM infiltration in the TME of TC occurs via the recruitment of circulating monocytes. Cytokines and chemokines within the TME facilitate macrophage polarization and influence TAM functions, including the promotion of tumor proliferation, stemness, gene instability, blood and lymphatic vessel proliferation, and immunosuppression. High TAM density is a characteristic feature of poorly differentiated papillary thyroid cancer (PTC) and is associated with poor prognosis. This suggests that TAMs may play a role in supporting the growth of PTC. Fiumara et al. used CD68 immunohistochemistry to assess TAMs in PTC and found that they inhibited metastasis in a subset of patients. Among 121 PTC cases, 15% had CD68+ TAMs with phagocytic activity against tumor cells. Patients with CD68+ cells showed significantly lower vascular invasion, distant metastases, and reduced lymphocyte and dendritic cell infiltration compared to those without. These findings suggest that CD68+ TAMs may enhance the host immune response against tumor cells [241,242,243,244].

Caillou et al. described TAM morphology and their proximity to cancer cells, suggesting a role in tumor growth and metastasis. However, thyroid cancer tissue morphology remained unchanged regardless of TAM presence, indicating a limited role in TC development. The activation of a pro-tumoral microenvironment by senescent thyroid cells indicates their detrimental impact. Targeting senescent cells and their effectors may thus serve as a therapeutic strategy for thyroid tumors. The in vitro results are corroborated by the co-expression of the *PTGS2* gene (COX2) and M2 markers, which have been detected in a significant proportion of human TC [245,246].

### 3.4. Esophageal Cancer

Esophageal cancer (EC) is the sixth leading cause of cancer-related mortality worldwide, with a higher prevalence in Western countries. Due to the absence of distinct clinical symptoms, nearly 40% of patients are diagnosed at advanced stages with metastases, rendering them inoperable. In this group, overall survival (OS) following conventional therapy is typically less than one year. Histologically, primary EC could be divided into esophageal squamous cell carcinoma (ESCC) and esophageal adenocarcinoma (EAC) with different types of metastases [247,248].

Metastases in ECs can be classified as lymphatic, hematogenous and direct diffusion. The latter occurs when the tumor invades surrounding tissues. Lymphatic metastasis is the primary mechanism of EC spreading and a key prognostic factor. The process involves multiple factors, including cytokines, chemokines, proteins, and cancer-associated cells [249].

In EC, immune cells contained in the TME control prevent further EC development in early stages. In later stages, EC cells effectively suppress the immune response by inhibiting the expression of cell surface proteins or releasing immunosuppressive proteins by limiting the expression of HLA proteins. At the same time esophageal cancer cells generate inhibitory signals and proteins, including PD-L1/2, VISTA, CTLA-4, LAG-3, and TIM-3. Notably, CTLA-4 is typically expressed on immune cells rather than on cancer cells, leaving its role in esophageal cancer cells unclear. EC cells release high-mobility group box 1 (HMGB1), an evolutionarily conserved DNA-binding nuclear protein, into the tumor microenvironment. There, HMGB1 interacts with Toll-like receptors (TLRs), promoting cancer cell proliferation. Its overexpression further facilitates immune evasion by enhancing the proliferation and differentiation of MDSCs, B cells, and T cells [250,251].

#### 3.4.1. Non-Immune Cells in Esophageal Cancer

The sources of CAFs in esophageal cancer are primarily normal fibroblasts, pericytes, and MSCs. Seven different CAFs subtypes have been identified in esophageal cancer, with the most common being myCAFs and iCAFs. CAFs in esophageal cancer secrete IL-6, IL-8, CCL5, CXCL1, and TGF-β, leading to the activation of signaling pathways such as Akt, STAT3, ERK1/2, and NF-κB, which affect cancer cell survival, proliferation, and migration. CAFs influence macrophage polarization and migration by secreting cytokines such as CCL2, IL-6, and CXCL8. TGF-β, IL-6, and FGF produced by CAFs contribute to chemoresistance in esophageal cancer. It has been shown that the use of TGF-β and IL-6 inhibitors sensitizes cancer cells to cisplatin [30,252,253,254,255,256,257,258,259].

Obesity is one of the main risk factors of esophageal cancer development. EC is predominantly associated with abdominal adiposity, not with the BMI index. Obesity leads to the transformation from squamous cell carcinoma to adenocarcinoma [260,261,262].

Adipocytes release leptin, a 16 kDa protein regulating energy consumption homeostasis. Leptin is also involved in the regulation of EMT-related genes (a-SMA-alpha-smooth muscle actin and E-cadherin. In esophageal adenocarcinoma cell lines, leptin promotes cell proliferation and activates JAK2, ERK or Akt signaling pathways leading to cancer progression. In patients with esophageal adenocarcinoma, an increase in adipocyte size is observed together with angiogenesis/lymphangiogenesis and increased leptin expression [263].

#### 3.4.2. Immune Cells in Esophageal Cancer

In EC patients, high numbers of CD8+ T cells infiltrating tumor tissue positively correlate with the prognosis and long-term survival [264].

T cells targeting cancer/testis antigen 2 (CTAG2) and melanoma-associated antigen 3 (MAGE-3), highly expressed on esophageal cells, play a key role in the anti-cancer immune response. Prognosis depends on the cytotoxicity of T cells and NK cells. High levels of immunosuppressive factors (IL-10, TGF-β) released by Tregs suppress CD8+ T cells and NK cell activity. Tregs promote CD8+ T cell exhaustion in EC, inducing cancer progression. They facilitate also cancer cell migration and angiogenesis through cytokine release and serve as a negative prognostic factor for overall survival. Tregs are recruited to the tumor mass by chemokines released by stromal cells [265,266,267].

NK cells are involved in the process of immune surveillance and play a role in anti-cancer immunity. The number of the CD56^dim^ NK cells that infiltrate tumors in EC patients gradually decreases with the cancer progression [250].

B cells response can be considered as a predictive marker in EC. High infiltration of tumor tissue by B cells means better prognosis and clinical outcome, although their precise role in initiating and supporting immune response in EC is unclear. In turn, high numbers of Bregs (IL-10+CD19+CD1d^high^CD5+) in EC patients corresponds to worse prognosis. Bregs suppress CD8+ T cells activation by releasing TGF-β and IL-10 that corresponds to poor clinical outcomes [268].

In EC patients, dendritic cells present the MAGE-A3 antigen, triggering a specific immune response. DCs in esophageal tissues primarily consist of Langerhans cells, which originate from bone marrow and express CD1a on their surface. The population of these cells correlates positively with more frequent angiogenesis and more aggressive course of EC [269,270]. In EC patients, the number of intratumoral dendritic cells (IDCs) is associated with survival time, which is longer the higher the number of IDCs. Therefore, the number of IDCs may be considered as a prognostic factor in these patients [271].

DCs isolated from peripheral blood and surgical tissues of EC patients express the costimulatory molecules CD83, CD80, and CD86. However, their ability to induce an immune response is significantly reduced compared to healthy individuals [272].

The activation of MDSCs in EC patients is regulated by IL-6 and signaling pathways mediated by aldehyde dehydrogenase 1. A high number of MDSCs correlates with poor prognosis due to their strong immunosuppressive properties. IL-6 is mainly secreted by monocytes and macrophages and stimulates inflammation. Higher IL-6 levels in the TME lead to increased MDSC infiltration [272].

In EC patients, CD38 is a key marker of MDSCs, playing a crucial role in modulating their immunosuppressive activity. Higher CD38 expression enhances MDSCs’ ability to suppress T cell activation and promote cancer progression. Ex vivo studies have identified IL-6, insulin-like growth factor-binding protein 3 (IGFBP3), and CXCL16 as factors that induce CD38 expression on MDSCs [254,273].

### 3.5. Gastric Cancer

Gastric cancer (GC) is considered one of the most common cancers worldwide. Its major cause appears to be chronic infection with *Helicobacter pylori*, which is associated with mucosal atrophy and increased pH levels in the stomach. A direct complication of bacteremia is intestinal metaplasia. *H. pylori* also release peptidoglycan that upregulates the levels of pro-inflammatory cytokines IL-8 and COX-2, contributing to the development of chronic inflammation in the TME and promoting oncogenesis. However, excess body mass, smoking, alcohol consumption, and gastroesophageal reflux disorders are also mentioned as contributing factors to the development of GC. Patients diagnosed with GC usually have a poor prognosis (the 5-year survival rate is approximately 6%). This type of cancer primarily affects older men from lower socio-economic backgrounds. In addition, several genetic risk factors should be considered during the diagnosis of gastric cancers (GCs), including blood group (A group), pernicious anemia, and hereditary non-polyposis colon cancer, as they predispose individuals to the development of GCs. On the other hand, high consumption of salts and nitrates, along with low levels of vitamins A and C, also predispose individuals to GCs [274,275,276].

Histologically, GC can be subdivided according to several classifications, among which the WHO classification and the Lauren classification are the most important. The WHO classification divides GC into papillary, tubular, mucinous, and poorly cohesive types. On the other hand, classification based on the expression of specific genes or molecular changes is more useful in choosing an appropriate treatment strategy. For instance, antibodies against HER2 (trastuzumab) are effective only in patients with HER2/neu-positive gastric cancer [277].

Each gastric cancer has its own genetic profile influenced by both cancer epithelial cells and the cells of the cancer TME. The detailed mechanisms by which the cells present in the TME drive gastric cancer phenotypes cells present in the TME drives GCs phenotypes is poorly understood [278].

The strong acidic environment and unique endocrine system of the stomach also contribute to shaping the TME of gastric cancer, with effects that are adverse to the overall situation. In gastric cancer, the TME is classically composed of the extracellular matrix (ECM), fibroblasts, endothelial cells, MSCs, macrophages, lymphocytes, neutrophils, and other components, all of which play a role in the progression of gastric cancers [69].

#### 3.5.1. CAFs in Gastric Cancer

In gastric cancer, CAFs arise from normal fibroblasts, cancer stem cells, and MSCs. CAFs with high expression of α-SMA and B7-H3 are associated with poor patient prognosis. On the other hand, CAFs with high expression of FAP are associated with the creation of an immunosuppressive environment, angiogenesis, and increased metastatic potential in gastric cancer [279,280,281,282].

In GC, CAFs promote cell proliferation by secreting dipeptidyl peptidase-4 (DPP-4) and C-X-C chemokine receptor 4 (CXCR4). Additionally, IL-6 produced by CAFs induces STAT3 activation, which facilitates GC cell proliferation [283,284].

CAFs play an important role in maintaining the stemness of GC cells likely through TGF-β. CXCL12 produced by CAFs can influence gastric cancer cells invasion by clustering integrin β1 on their surface [285,286]. CAFs responsible for remodeling the TME promote EMT, increase tumor invasion and migration by creating a collagen-rich matrix. CAFs can generate gaps in stromal components and the basement membrane in an MMP-dependent manner, allowing cells to escape from the primary tumor [287,288].

CAFs can promote angiogenesis by overexpressing Galectin-1, which can affect VEGFR2 phosphorylation and VEGF expression. They can also directly stimulate angiogenesis by secreting CXCL12 [289]. CAFs inhibit the antitumor functions of T cells by secreting CXCL12, as well as exosomal OIP5-AS1, which reduces miR-142-5p levels and increases PDL-1. Extracellular vesicles containing annexin A6 from CAFs induce FAK-YAP activation and tubular network formation by stabilizing integrin β1, thereby increasing cisplatin resistance in GC. Additionally, miR-522 from CAFs increases resistance to cisplatin and paclitaxel in GC. Furthermore, brain-derived neurotrophic factor (BDNF) from CAFs provides resistance to anlotinib by stimulating TrkB [290,291,292].

#### 3.5.2. Adipocytes in Gastric Cancer

Adipocytes play a crucial role in GC development. FFAs released by them promote peritoneal metastasis of GC, while in omentum (where abdominal fat tissue is localized), the migration of gastric cancer cells can be increased by several cytokines. In turn, adiponectin suppresses the proliferation of GC cells through adiporeceptor, which is an indicator of a longer survival. Infusions of adiponectin into the peritoneal cavity slightly limits the number of peritoneal metastatic nodules in murine models of GC. In advanced stages of GC, cancer cells penetrate gastric bare areas and cooperate with the adipocytes changing their phenotype towards cancer-associated adipocytes, CAAs [293,294,295].

High adipocyte levels in GC correlate with worse prognosis and clinical outcomes. Genetic studies link four adipocyte-expressed genes—alcohol dehydrogenase 1B (*ADH1B*), secreted frizzled-related protein 1 (*SFRP1*), placenta-specific 9 (*PLAC9*), and fatty acid-binding protein 4 (*FABP4*)—to GC development. Increased *ADH1B* copy numbers during adipocyte differentiation correlate with a higher GC risk. In GC patients, elevated *SFRP1* levels are associated with advanced disease and poor prognosis, likely via TGF-β pathway activation, promoting proliferation and EMT. FABP4, a lipid transport protein involved in PPAR-γ (peroxisome proliferator-activated receptor gamma) regulation, is downregulated in poor-prognosis cases but upregulated in tumors with higher immune cell infiltration. Expression of ADH1B, SFRP1, PLAC9, and FABP4 positively correlates with memory CD4+ resting T cells and memory B cells, while macrophages and neutrophils are reduced in the GC TME. High adipocyte expression appears linked to greater GC aggressiveness [296].

#### 3.5.3. CSCs in Gastric Cancer

Gastric cancer stem cells (GCSCs) can be labeled with the biomarker *Villin* (V-GCSC), an actin-binding protein specific to epithelial cells. Given that V-GCSC are found in the lesser curvature of the antrum, a location where GC are frequently present, it has been postulated that the transformation of V-GCSC may result in GC formation [297,298,299].

#### 3.5.4. T Cells in Gastric Cancer

CD8+ T cells predominate in the TME of GC due to their role in cytotoxic elimination of tumor cells, although CD4+ T cells are represented by large numbers of Treg cells. The reduction and dysfunction of CD8+ T cells is believed to be one of the important causes of the development of immune tolerance in gastric cancer. High expression of the CD276 (B7-H3) marker in stomach or intestine correlates with a decrease in the CD8+ T cells density within the tumor mass, suggesting that this marker may be involved in the immune response evasion of cancer cells. Moreover, expression of TLR2 and CD276 on the surface of the CD8+ T cells is downregulated in patients with GC, which affects the release of perforins and granzymes. In GC, the population of CD103+CD4+ T cells contributes to inhibiting the immunosuppressive phenotype and has high retention capacity. This results in CD8+ T cell dysfunction and reduced levels of granzyme B, IFN-γ, and TNFA [300].

In gastric cancer, prognosis depends not on the total number of Tregs but on their localization—stromal Tregs are associated with better outcomes, whereas epithelial Tregs correlate with a poorer prognosis. Gastric cancer cells induce Treg generation by releasing TGF-β1, with higher TGF-β1 levels leading to increased Treg numbers. TNFR2-positive Treg infiltration increases with GC progression and serves as a prognostic marker, as the TNFA/TNFR2 pathway enhances the immunosuppressive phenotype of Tregs. Additionally, Tregs upregulate PDL-1 expression in MSCs via the p-STAT3 pathway, promoting immune tolerance and accelerating cancer progression in GC patients. In contrast, gastric mucosal infections with *Stenotrophomonas* and *Selenomonas* have negative impact on the number of Tregs and pDCs [300].

#### 3.5.5. NK Cells in Gastric Cancer

The number of the CD56^dim^ NK cells that infiltrate the TME gradually decreases with the progression of cancer in patients with GC, although the expression of the activatory and inhibitory KIRs on the NK cells is similar in patients with GC as in healthy individuals. Only the effector function of NK cells is impaired, due to lower production of IFN-γ, TNF-α and Ki-67. A low percentage of cancer-infiltrating NK cells corresponds to the cancer progression and low OS [301].

The level of the NKG2D activatory receptor on NK cells in GC patients positively correlates with prognosis and OS rates. Although inactive NK cells exhibit limited cytotoxicity against gastric cancer cells, their activation induces a strong anti-cancer response. Factors such as MMP2 and MMP9 enhance NKG2D expression, “sensitizing” gastric cancer cells to NK cell-mediated attack [302,303].

#### 3.5.6. B Cells in Gastric Cancer

In GC patients, IL-10-secreting B cells in the TME stimulate CD4+ and CD8+ T cells to release antitumor cytokines. Additionally, CD20+ B cells are associated with a lower likelihood of nodal and lymphatic metastasis, likely due to their production of anti-sulfated glycosaminoglycan (GAG) antibodies, which inhibit cancer growth and progression [304].

In GC patients infected with Epstein–Barr virus (EBV) or *Helicobacter pylori*, susceptibility to gastric cancer is significantly increased. Those with *H. pylori* infection show a high frequency of IL-10-secreting B cells expressing CD24 and CD38, while naive and resting memory B cells are reduced. Elevated IL-10 secretion by B cells limits T effector cell function and inhibits antigen presentation, highlighting the conflicting role of B cells in GC [305].

#### 3.5.7. Monocytes and Related Cells in Gastric Cancer

Monocytes are key components of the TME in GC, where they are recruited from peripheral blood. They are among the primary immune cells infiltrating the tumor and exhibit metabolic alterations, including changes in serotonin metabolism, histamine receptor function, and hydroxycarboxylic acid-binding receptors (HCARs), compared to healthy individuals. These cells are predominantly of CD14+CD16- phenotype (intermediate monocytes), losing their anti-cancer activity and suppressing other immune cells. They influence cancer progression by modulating the MIF and TNF pathways, directly promoting tumor growth. Additionally, they indirectly support cancer development by interacting with macrophages, B cells, and T cells. High-risk group GC patients exhibit increased monocyte levels, along with higher numbers of dendritic cells and M2 tumor-associated macrophages (TAMs) [306].

Clinical observations show that a high absolute number of lymphocytes and monocytes is linked to better prognosis in GC. Combining lymphocyte and monocyte counts improves prognostic prediction in stage II/III GC patients. Additionally, monocyte count correlates with OS in this group [307,308].

The role of pDCs in gastric cancer remains unclear. In peripheral blood, pDCs, Tregs, and ICOS+ Tregs are elevated compared to healthy individuals, with circulating pDC levels significantly higher in advanced cancer stages and cases with lymph node metastasis. Within the TME, pDCs exhibit immunosuppressive and tolerogenic properties. A high pDC percentage in tissues correlates with larger tumor mass. The BDCA-2+ (a novel C-type lectin contributing to the potential suppression of secretion of IFNa/b in pDCs) pDC subpopulation, linked to potential suppression of IFN-α/β secretion, is homogeneously distributed in gastric cancer tissues but is particularly elevated in EBV-positive patients, where it is associated with the diffuse-type of cancer [309,310].

The presence of Tregs and MDSCs in the TME of GC patients is associated with worse outcomes due to their role in suppressing cancer antigen-specific T cells. MDSCs in patients with GC express higher levels of immunosuppression-related genes. The discovery of immediate early response 3 (IER3)-positive mo-MDSCs highlights their potential as targets for future immunotherapies in GC patients. IER3, expressed on the surface of mo-MDSCs and T cells, protects cells from apoptosis. In GC patients, IER3-positive MDSCs are abundant in cancer tissue, correlate with poor prognosis, and are more prevalent in individuals resistant to gemcitabine treatment [311,312].

#### 3.5.8. Other Immune Cells in Gastric Cancer

Elevated neutrophil counts in both peripheral blood and tumor tissues have been demonstrated to predict a poor prognosis in patients with gastric cancer (GC). Tumor-associated neutrophils (TANs) have been shown to produce interleukin IL-17a, which promotes epithelial–mesenchymal transition of GC cells via JAK2/STAT3 signaling. Blockade of IL-17a signaling with a neutralizing antibody has been observed to inhibit TAN-stimulated activity in GC cells [313].

In TME, TAMs and cancer cells interact via mediators such as cytokines that facilitate cell growth. M2 TAMs enhance the migratory and invasive abilities of GC cells by increasing MMP2 secretion and promoting EMT. In parallel, Zhang et al. showed that mixed M1 and M2 TAMs facilitated the malignant behavior of diffuse gastric cancer by increasing CAF activity via IL-1β secretion [75,314].

In another study, Qiu and colleagues demonstrated that M2 TAMs facilitate the development of liver metastasis in gastric cancer by influencing the microenvironment prior to metastasis and the subsequent development of angiogenesis following metastasis. Notably, in tissues with high infiltration of M2 TAMs, high microvessel density was observed [258,315].

### 3.6. Pancreatic Cancer

Pancreatic cancer (PanC) is one of the deadliest malignant tumors in the world. Its incidence rate nearly matches its mortality rate. Symptoms of PanC usually do not appear until the disease reaches an advanced stage and metastasis occurs. As a result, most cases of pancreatic cancer are diagnosed too late, making treatment very difficult. The two main types of PanC are adenocarcinoma, which accounts for approximately 85% of cases, and pancreatic neuroendocrine tumors, which make up less than 5% of all cases. Pancreatic ductal adenocarcinoma (PDAC) occurs in the exocrine tissue, while pancreatic neuroendocrine tumors (PanNET) occur in the endocrine tissue of the pancreas [316,317,318,319].

Genetic mutations commonly associated with PanC include mutations in Kirsten rat sarcoma viral oncogene homolog (KRAS), tumor protein p53 (TP53), smothers against decapentaplegic homolog 4 (SMAD4), also known as deleted in pancreatic cancer 4 (DPC4), and cyclin-dependent kinase inhibitor 2A (CDKN2A). PanC is extremely complex in terms of its genomic, epigenetic, and metabolic characteristics. It involves the activation of multiple pathways and interactions of various cells within the TME. The TME in the PanC impacts their development, metastatic potential and resistance to the therapeutic strategies [320].

#### 3.6.1. CAFs in Pancreatic Cancer

In PanC, CAFs primarily originate from resident pancreatic fibroblasts, pancreatic stellate cells, and mesenchymal stem cells infiltrating the tumor. Additionally, other sources can include bone marrow cells, adipocytes, pericytes, fibrocytes, smooth muscle cells, mesothelial cells, and endothelial cells. CAFs account for up to 90% of the tumor tissue in PDAC [321].

In this cancer, three unique clusters of CAFs with distinctly different functions have been identified. These are steady-state-like (SSL) CAFs, mechanoresponsive (MR) CAFs, and immunomodulatory (IM) CAFs (Figure 4). MR CAFs are activated in response to mechanical stimuli from the TME. IM CAFs have immunoregulatory functions. SSL CAFs can transform into MR or IM CAFs when needed. Within these clusters, there are many subtypes of CAFs. The most important in pancreatic cancer are iCAFs, myCAFs, and apCAFs (see also Section 2.1.1). The most prevalent subtypes of CAFs in PDAC are myCAFs and iCAFs. Different subtypes of CAFs are located in various areas within the pancreatic tumor microenvironment. myCAFs have been shown to be distributed near cancer cells, whereas iCAFs are located farther from the cancer cells. In PDAC, the desmoplastic stroma mainly consists of myCAFs. PanC cells stimulate both myCAFs and iCAFs. myCAFs generate desmoplasia surrounding the tumor, and they can both inhibit and facilitate tumor progression. myCAFs inhibit tumor progression by encapsulating the tumor in a dense collagen matrix, which constricts and blocks cancer cells from progressing and invading. In turn, they facilitate tumor progression by creating an active barrier against radiation therapy. myCAF in PDAC induces metastasis through induction of type III collagen hypertrophy via the IL-33/ST2/CXCL3/CXCR2 axis. Metastatic CAFs exhibit also higher expression of heparan sulfate proteoglycan 2 (HSPG2), which promotes metastasis. iCAFs inhibit the immune response to pancreatic tumors by secreting factors that suppress immune recognition and antitumor defense [28,30,103,322,323,324,325,326].

In PDAC, TGF-β induces transformation of quiescent fibroblasts into CAFs. Activated CAFs themselves secrete TGF-β, promoting tumor cell growth and extracellular matrix (ECM) deposition [327]. In PDAC, CAFs play a dual role in modulating the immune system. Firstly, they hinder the function of immune cells by forming a dense fibrotic stroma mainly composed of immunosuppressive cells such as DCs, TAMs, MDSCs, Tregs, and cytotoxic T cells TAMs, MDSCs, and Tregs suppress antitumor responses contributing to tumor growth Additionally, some CAFs can cleave type I collagen (COL 1), enhancing macrophage adhesion and potentially influencing interactions between fibroblasts and immune cells in the TME. Moreover, CAFs contribute to immune evasion by expressing immune checkpoint ligands such as cytotoxic T-lymphocyte-associated antigen 4 (CTLA-4) and programmed death-ligand 1 (PD-L1), which engage effector T cells Cancer cells secrete paracrine factors such as IL-1, which activate NF-κB signaling and the expression of leukemia inhibitory factor (LIF) in iCAFs. LIF secreted by iCAFs subsequently activates the STAT3 signaling pathway in cancer cells, which promotes tumor progression [21,328,329,330,331].

Pancreatic CAFs produce growth factors, chemokines, and cytokines that promote tumor growth. Increased SHH signaling in PDAC induces insulin-like growth factor 1 (IF1) expression and growth arrest-specific (GAS6) expression, leading to Akt signaling activation in cancer cells. This, in turn, enhances proliferation and resistance to apoptosis in cancer cells [332,333].

In PDAC, CXCL12 secreted by CAFs contributes to tumor progression and gemcitabine resistance by enhancing SATB expression. CAFs increase resistance to gemcitabine by transferring miR-106b, targeting the TP53INP1 gene, directly to pancreatic cancer cells [334,335].

CAFs can inhibit tumor growth by promoting an antitumor immune response. CAFs produce immunomodulatory cytokines such as IL-10, TGF-β, TNF, IFN-gamma, IL-6, CXCL9, CXCL10, and CXCL11, which can also recruit immune cells like T lymphocytes, macrophages, and natural killer cells to the TME. CAFs can also stimulate the differentiation of monocytes into M1 macrophages, which have antitumor properties. Studies on mouse models of PDAC have shown that the population of CAFs expressing SMA exhibit increased Shh signaling, which inhibit the production of VEGF, CXCL12, and IL-8, factors that promote angiogenesis, tumor growth, and immunosuppression in the TME. Other studies have demonstrated that targeting FAP+ CAFs resulted in the recruitment of CD8+ T lymphocytes and inhibition of collagen synthesis, allowing for immune control over tumor growth [9,325,336,337,338].

In PDAC, stromal fibrosis restricts the availability of nutrients and oxygen. As a result, PanC cells undergo metabolic reprogramming, shifting from oxidative phosphorylation (OXPHOS) to glycolysis, that allows them to adapt and survive under altered conditions. Additionally, PDAC cells can utilize CAFs to obtain nutrients, a phenomenon known as the reverse Warburg effect. In this scenario, CAFs, being glycolytic cells, release lactate and pyruvate into the intercellular space, which are taken up by cancer cells to fuel OXPHOS. CAFs enhance glycolytic metabolism of cancer cells by secreting paracrine hepatocyte growth factor (HGF). Moreover, CAFs can provide alanine through autophagy, serving as an alternative carbon source that supports the metabolism and growth of cancer cells. CAFs can also indirectly nourish cancer cells: it has been demonstrated that cancer cells can absorb collagen and hyaluronic acid from the TME [339,340,341,342,343,344,345].

In pancreatic tumors, MR CAFs exhibit increased reactivity to mechanical stimulation within the TME. They are characterized by elevated levels of mechanosensitive signaling mediators, mechanosensors such as integrins and focal adhesion proteins, and increased expression of genes such as *POSTN*, *FOSB*, *MGP*, and *GAS6*, which are associated with mechanotransduction and the focal adhesion kinase (FAK) signaling pathway. MR CAFs participate in ECM remodeling and influence its stiffening through increased deposition of collagen and fibronectin fibers. This facilitates cancer cell invasion and metastasis. In response to mechanical signals from the TME, MR CAFs activate multiple signaling pathways, including TGF-β, Yes-associated protein (YAP), and MAPK. The presence of MR CAFs in pancreatic cancer is associated with increased tumor aggressiveness, therapy resistance, and reduced patient survival [12,323,346].

#### 3.6.2. Stellate Cells in Pancreatic Cancer

Activated pancreatic stellate cells in pancreatic cancer enable immune system evasion, promote tumor growth and metastasis formation, stimulate angiogenesis, impair immune system function, and contribute to resistance to anti-cancer therapies [347,348].

PSCs inhibit T lymphocyte infiltration both directly and indirectly through cytokine secretion. PSCs produce NF-kB, which increases CXCL12 secretion, thereby reducing cytotoxic T lymphocyte infiltration, promoting pancreatic cancer cell survival and tumor growth. Activated PSCs regulate chemokines, cytokines, and T lymphocyte adhesion molecules, thus reducing CD8 T lymphocyte migration to the peritumoral stromal compartments, which blocks the antitumor immune response. Activated PSCs have the ability to suppress the antitumor immune response also by negatively affecting NK cells [349,350,351]

PSCs exhibit increased activity of glutamine anabolic pathways compared to pancreatic cancer cells. PSCs activate the β-catenin/Wnt/TCF7 pathway to enhance glutamine synthesis and secretion and accelerate pancreatic cancer cell growth. They also utilize autophagy-derived alanine to promote lipid biosynthesis and the production of non-essential amino acids, thereby supporting cancer cell growth [352,353].

Aging PSCs exhibit tumor-promoting functions: they promote cancer cell proliferation and migration through the CXCL1/CXCR2 axis. Conversely, antagonists of the CXCL1/CXCR2 axis weaken this effect [354].

PSCs secrete leukemia inhibitory factor (LIF), which regulates cancer cell differentiation and EMT, as well as influences chemotherapy resistance. PSCs also secrete Wnt and tenascin C (TnC), which activate the oncogenic signaling pathways of β-catenin and YAP/TAZ, promoting cancer cell survival [355].

#### 3.6.3. Adipocytes in Pancreatic Cancers

Pancreatic cancer cells can significantly affect the role and activity of adipocytes because they require nutrients for proliferation and differentiation. Obesity in patients with PanC is a recognized risk factor for cancer progression and metastasis. Typically, PanC is surrounded by visceral adipose tissue, which may promote cancer cell proliferation, angiogenesis, and migration [356].

In PanC patients, steatosis of pancreatic tissue is a common issue. This condition, characterized by fat accumulation, can lead to pancreatic dysfunction, impairing both the endocrine and exocrine functions of the pancreas. Pancreatic cancer cells rely on glucose and free fatty acids (FFAs) for their growth and development. Under unfavorable aerobic conditions, these cells enhance glycolysis as well as lipid and glutamine metabolism to sustain their rapid proliferation. Disruption of lipid metabolism in cells is a hallmark of cancer. The strong upregulation of lipid metabolism-related proteins and enzymes accelerates the progression of malignant pancreatic cancers [356,357].

In PanC patients, the expression of ATP citrate lyase (ACLY) is elevated, which negatively impacts prognosis. Similarly, the level of fatty acid synthase (FAS) in cancer cells is increased and is also associated with a worse prognosis in PanC patients. Cancer cells produce and utilize FFAs for the generation of phospholipids and signaling molecules. Therefore, targeting the enzymes involved in FFA release can serve as an effective therapeutic strategy in the context of PanC treatment. Additionally, cancer cells rely on FFAs for β-oxidation, a process essential for providing the energy required to sustain their proliferation. Cancer cells often obtain FFAs from adipocytes or low-density lipoproteins (LDLs) circulating in the peripheral blood. These FFAs are normally stored as a lipid droplet in PanC cells which protects cells from lipotoxicity and toxicity related to ROS. At the same time, adipocytes secrete glutamine protecting PanC cells in nutrient-depleted conditions [358,359].

Furthermore, during the PanC development, the communication between PanC cells and adipocytes is provided by inflammatory cytokines secreted by FcyRIIIA cancer cells. They stimulate adipokine generation leading to the development of the inflammation, tissue fibrosis and adipocyte death. Adipocytes release lipocalin 2 (Lcn-2) after contact with PanC cells, facilitating muscle atrophy and enhancing fibrosis process. During cancer development, adipocytes gradually lose their characteristic features (secretion of adiponectin, leptin, resistin, FABP4) and transform into cells expressing markers typical for fibroblasts (α-SMA-α smooth muscle actin, S100A4; ferroptosis suppressor protein 1, FSP-1; plasminogen activator inhibitor-1, PAI-1; and MMP9 and 11). In patients with PanC, tumors surrounded by peri-pancreatic fat infiltration are linked to an advanced cancer stage, locoregional recurrence, and shortened overall survival (OS) [360,361].

#### 3.6.4. Cancer Stem Cells in Pancreatic Cancer

The self-renewal, proliferation and resistance to treatment exhibited by CSCs in PDAC are regulated by several key signaling pathways. One of the pathways is Wnt/β-catenin, which plays a pivotal role in regulating the “stemness” of cancer cells and influencing the processes of differentiation and proliferation. In PanC, its activation has been linked to more aggressive tumor development and increased resistance to treatment. Furthermore, WNT plays a role in the capacity of CSCs to evade therapeutic intervention, thereby contributing to disease recurrence. Also, activation of the Notch pathway contributes to the development of chemoresistance and tumor progression, and its inhibition. The Hedgehog (SHH) pathway exerts control over numerous aspects of cancer cell “stemness,” including their capacity to proliferate and instigate tumor formation. In PanC, the activation of this pathway contributes to the formation of a dense desmoplastic stroma, which impedes drug delivery and also supports the survival of CSCs [360,362].

#### 3.6.5. T Cells in Pancreatic Cancer

T cells are considered to be the most important cells of the TME of PDAC, and their phenotype and activity correspond to the aggressive course of cancers. T cell distribution in PanC is highly heterogenous and the T cell infiltrates are mostly diffused. In addition, a high percentage of these cells is associated with the better OS in PDAC patients after resection [361,363].

In PanC patients, many stromal cells including γδ T cells or Tregs suppress immune anti-cancer response by releasing cytokines. PanC cells can evade T cell responses by undergoing mutational changes in their phenotype, upregulating immune checkpoint expression, or releasing immunosuppressive cytokines such as TGF-β [364].

Tregs contribute to the suppression of T cell immune responses by inducing apoptosis in activated CD4+ T cells and reducing cytokine accumulation. In PanC, the tumor microenvironment is predominantly infiltrated by CD4+ T cells (Th2) rather than CD8+ T cells. Alongside Th2 cells, Tregs and Th17 cells are also present in the tumor mass. The number of Tregs is significantly elevated in PanC patients, both in peripheral blood and within the tumor mass, and is associated with poor prognosis and unfavorable clinical outcomes. Tregs promote pancreatic cancer progression via the TRAIL pathway and by sequestering IL-2, impairing the function of other immune cells. Additionally, non-classical T cells, γδ T cells, constitute approximately 40% of the T cell population infiltrating PanC tumors. These cells support cancer progression and inhibit the function of classical αβ T cells [364,365].

#### 3.6.6. NK Cells in Pancreatic Cancer

In patients with stages Ib-III PDAC, a positive correlation was observed between the number of NK cells in peripheral blood before and after surgery and the time to disease relapse. Specifically, patients with higher NK cell counts experienced longer disease-free survival. Similarly, the NK cells infiltrations in the TME correlated with delayed recurrence. This correlation is linked to high levels of type I and type II IFNs, which activate NK cells. These IFNs also induce CXCL10 expression, facilitating NK cell recruitment to cancer tissue and serving as a positive prognostic marker for PDAC patients [366].

In PanC patients, the percentage of the activating KIR receptors (DNAM1—CD226) and CD96 on NK cells is low, and reduced expression of these molecules correlates with the tumor histological grade and lymph node metastasis. It is hypothesized, that in PanCs, there are distinct subtypes of NK cells with impaired activities and that the final prognosis depends not only on the NK cells number, but also on their selected functionality [367].

#### 3.6.7. B Cells in Pancreatic Cancer

In general, B cell presence is associated with better clinical outcomes in patients with PDAC. In PDAC models, B cells number corresponds with the limited tumor mass and longer survival. B cells play a key role in fibrogenesis during PDAC progression. The population of circulating plasmablasts with fibrogenic properties is significantly expanded in both the peripheral blood and the TME of PDAC patients. B cells infiltrate cancer lesions, often in response to signals such as CXCL-13, and exhibit a regulatory phenotype (Bregs). These Bregs promote cancer cell growth by releasing TGF-β and IL-10, which suppress CD8+ T cell responses. Additionally, B cells in PDAC can recruit inflammatory cells, including Th2 cells, eosinophils, and TAM M2 macrophages, all of which contribute to the fibrotic potential of the TME [368].

It is proposed that B cell activity in non-metastatic PDAC depends on their localization. Favorable outcomes are associated with the B cells localized in TLS, while poor OS is expected when they are diffused within the tumor mass [369,370].

#### 3.6.8. Monocytes in Pancreatic Cancer

In PDAC patients, a high percentage of circulating monocytes corresponds to higher cancer aggressiveness. The strong activation of monocytes suggests their involvement in rapid cancer progression. Notably, monocytes exhibit both anti-cancer and pro-cancer activities simultaneously. This dual role is attributed to their ability to differentiate into tumor-associated TAMs, which promote cancer progression and metastasis. In PDAC, circulating monocytes suppress T cell activity while also inhibiting Treg function. Simultaneously, they contribute to NK cell activation, primarily influencing IL-10 release by NK cells. Moreover, in PDAC patients, TNF-α induces epithelial–mesenchymal transition (EMT), which facilitates development of stroma within the tumor. Consequently, an increased number of monocytes correlates with higher TNF-α levels and a more aggressive disease course. Additionally, PDAC patients show an elevated proportion of intermediate monocytes, which is associated with higher cancer grades, highlighting their potential role in cancer progression [114,371].

PanC patients demonstrate constitutive phosphorylation of signal transducer and activator of transcription (STAT) family members and their impaired response upon stimulation by monocytes [371].

#### 3.6.9. Dendritic Cells in Pancreatic Cancer

In PDAC, increased number of DCs is associated with a better prognosis and better OS. Surgical resection of the tumor mass improves DCs activity in peripheral blood, supporting cancer’s impact on immune functions. DCs activity depends on the effect of CXCL17 and intercellular adhesion molecule 2 (ICAM2), and the TGF-β, IL-10 and IL-6 that suppress DCs proliferation. ICAM-2 plays a role in lymphocyte recirculation and antigen-specific immune response. The DCs activity may be also inhibited through NO secretion by MDSCs. Meanwhile, the process of antigen presentation in which DCs participate is impaired due to the fact that the expression of HLA class I and transporter associated with antigen processing (TAP) proteins are downregulated in almost 76% of PDACs patients [372].

#### 3.6.10. MDSCs in Pancreatic Cancer

In pancreatic cancer, MDSCs with the CD11b+CD33+HLA-DR- phenotype play an important role in immunosuppression. Elevated numbers of MDSCs and levels of cytokines secreted by them to the peripheral blood can be used as a predictive marker of failure of chemotherapy [372].

MDSCs recruitment into the tumor mass in PDACs depends on the oxygen availability, stromal pH and nutrient delivery, which influence MDSC activity. The Warburg effect is a key factor in PDAC development and correlates with cancer cell response to radiotherapy. In radioresistant cells, glycolysis and lactate production are elevated, contributing to radiotherapy resistance. Increased lactate levels after radiation enhance immunosuppressive MDSC activity, which impairs anti-cancer T cell responses and promotes PDAC progression [373,374].

Interestingly, PanC cells can induce proliferation and migration of MDSCs from the bone marrow directly to the TME using cytokines/chemokines/growth factors as “attractants”. Among them, granulocyte-macrophage colony-stimulating factor (GM-CSF) plays a crucial role. High levels of GM-CSF are observed in patients with pancreatic cancers bearing KRAS mutation (approximately 90% of PanCs) [370].

#### 3.6.11. Neutrophils in Pancreatic Cancers

MMP9 facilitates the release of VEGF from the extracellular matrix and contributes to the interaction between VEGF and its receptors, playing thus a pivotal role in angiogenesis. Neutrophils are a source of MMP9 in tumor angiogenesis in turn, the protein neutrophil gelatinase-associated lipocalin (NGAL) secreted by neutrophils has the potential to inhibit angiogenesis by reducing VEGF production in pancreatic cancer cells. Moreover, CXCL5 has been demonstrated to facilitate pancreatic cancer angiogenesis in a murine model through the activation of multiple signaling pathways, including the STAT and extracellular signal-regulated kinase (ERK) pathways in human endothelial cells [375,376].

#### 3.6.12. Macrophages in Pancreatic Cancers

A desmoplastic stroma observed in PDAC impedes the penetration of therapeutic agents. Macrophages are well documented for their role in the promotion of fibrosis in both physiological and pathological conditions. A recent study has demonstrated that subsets of TAMs are capable of directly depositing or remodeling the ECM, thereby facilitating the development of fibrosis. Consequently, TAM density is an independent prognostic factor associated with a higher risk of disease progression, recurrence, metastasis, and shorter OS in human PDAC patients. Additionally, TAMs have been demonstrated to contribute to chemoresistance against gemcitabine therapy. Resistance to gemcitabine develops within weeks of treatment, due to a combination of intrinsic resistance mechanisms and modulation of the TME [377,378,379,380,381].

It is worth noting that M2 macrophages promote angiogenesis by suppressing E2F2 expression in TECs. Interactions between TECs and M2 macrophages are mediated by exosomal miR-155-5p and miR-221-5p, derived from M2 macrophages [382].

### 3.7. Liver Cancer

Liver cancer (LC) is among the cancers with high mortality rates. It encompasses a heterogeneous group of malignancies with diverse histological characteristics and generally poor prognoses. LC includes hepatocellular carcinoma (HCC), intrahepatic cholangiocarcinoma (iCCA), mixed hepatocellular cholangiocarcinoma (HCC-CCA), fibrolamellar hepatocellular carcinoma (FLC), and the pediatric neoplasm hepatoblastoma, among which HCC and iCCA are the most common. The most common types are hepatocellular carcinoma (HCC; ~80% of cases) and intrahepatic cholangiocarcinoma (iCCA; ~20% of all primary liver cancers) [383,384].

The common risk factors of the HCC development are age (60–64 in men and 65–69 in women), sex (2–4 times often in men than in women), race/ethnicity, infection with HBV, HCV and other factors like alcohol, diabetes, metabolic syndromes and obesity [385].

The TME in HCC displays a chronic immunosuppressive and necro-inflamed profile, characterized by a typical decrease in the number of T cells and impaired T cell activity. In HCC, the TME promotes cancer progression and creates a specialized niche for liver cancer cells; however, it is important to note that there is significant variability in HCC among individuals [386].

Notably, the liver is a major site for the formation of metastases from extrahepatic tumors and approximately 25% of metastases occur in the liver. Risk factors include infection with hepatitis B virus, hepatitis C virus, liver steatosis, alcohol-related liver cirrhosis, smoking, obesity and diabetes [387,388,389].

#### 3.7.1. CAFs in Liver Cancer

In LC, CAFs originate from normal fibroblasts, hepatic stellate cells, MSCs, epithelial cells, and endothelial cells. Markers used to identify CAFs in liver tumors include α-SMA, FAP, PDGF, and FSP-1. In LC, three main subtypes of CAF can be distinguished: myCAFs, iCAFs, and PF/mesothelial CAFs (PF/mesCAFs) (Figure 4). CAFs in liver tumors can be clustered, sporadic, and located along liver sinusoids. The amount of CAFs is positively correlated with tumor size [390,391].

CAFs in LC secrete cytokines such as TGF-β, HGF, IGF, and IL-6. CAFs influence the immune response in the TME and affect the proliferation and migration of LC cells. CAFs remodel the ECM by synthesizing collagen and fibronectin, which stiffens the tumor stroma and impacts treatment resistance. Under normal conditions, the rate of ECM production in the liver is equal to its rate of degradation. However, in LC, there is excessive deposition of ECM components. CAFs play a key role in the deposition of collagen, one of the main components of the ECM, which influences the initiation and progression of liver cancer [391,392,393].

#### 3.7.2. Stellate Cells in Liver Cancer

Over 80% of HCC cases develop as a result of liver fibrosis. It has been shown that hepatic stellate cells promote the progression of HCC by influencing cancer cell proliferation, angiogenesis, and immunosuppression, as well as through the secretion of ECM components such as laminin [394,395].

Kupffer cells can activate HSCs, which can influence the remodeling and stiffness of the ECM. They can also create an immunosuppressive environment by recruiting Tregs and MDSCs, which can suppress CD8+ T cell functions [396,397].

Activated HSCs can influence tumor growth by inhibiting tumor dormancy. They secrete chemokine CXCL12, which reduces NK cell activity, thereby removing the blockade that inhibits metastasis growth. HSC-specific deletion of p53 is associated with liver fibrosis, development of HCC, and reduced survival. The p53-dependent senescence program in HSCs supports an immunosuppressive tumor environment by promoting a macrophage phenotype similar to M1. In contrast, the loss of p53 in HSCs supports M2 macrophage polarization and the progression of HCC [398,399].

#### 3.7.3. Other Non-Immune Cells in Liver Cancer

In hepatocellular carcinoma, TECs express glycoprotein non-metastatic melanoma protein B (GPNMB) to induce the exhaustion of tumor-infiltrating T cells [400].

The number of adipocytes in HCC correlates with a more aggressive cancer course and poor prognosis and clinical outcomes. The influence of adipocytes on LC cells results from the release of adipokines, which affect the functioning of liver cells. Adipocytes are responsible for the release of FFAs and glycerol from visceral adipose tissue (larger in men than in women) contributing to the onset of HCC. VAT size and age/male gender correlate positively with HCC development [401].

It has been demonstrated that CSCs in HCC undergo EMT, which enables them to infiltrate, circulate in the bloodstream, and extravasate to distant sites, forming metastatic lesions [402].

#### 3.7.4. T Cells in Liver Cancer

The TME in HCC exhibits chronic immunosuppressive and necro-inflamed characteristics, marked by reduced T cell numbers and impaired T cell activity. In liver cancer, CD4+ and CD8+ T cells localize primarily in the peritumoral zone, with tumor-infiltrating antigen-specific CD8+ T cells playing a key role in immune response and cancer control. A high CD4+/CD8+ T cell ratio correlates with a lower recurrence rate following liver transplantation for HCC. Additionally, CTL infiltration is associated with better prognosis and longer survival [403,404].

In HCC, Tregs exhibit immunosuppressive properties, while CD8+ T cells show impaired effector activity and express high levels of inhibitory receptors (PD-1, LAG-3, CD244, TIM-3, and CTLA-4). Patients with LC have elevated Treg levels in both peripheral blood and liver tissue, correlating with worse prognosis, rapid disease progression, and increased recurrence risk after liver transplantation, highlighting their role in LC development [403].

Alongside CD8+ T cells, CD4+ T cells play a key role in anti-cancer immunity. However, in LC, the Th1/Th2 ratio shifts towards Th2, promoting cancer immunosuppression. High Th2 levels are associated with poor prognosis, advanced tumor stages, and reduced treatment response [405].

In LC patients, Th17 cell infiltration is higher in tumor tissues than in tumor-free zones, correlating with shorter OS and disease-free survival (DFS). Notably, Th17 cell numbers are also elevated in HCC of viral origin [403].

Th22 cells comprise another important population of T cells in LC. They release IL-22 and are implicated in the pathogenesis of autoimmune diseases and cancer. Th22 accumulation in the liver protects hepatocytes from apoptosis but also promotes LC progression. High Th22 levels in both tumor infiltrates and peripheral blood are associated with rapid disease progression [406].

#### 3.7.5. NK Cells in Liver Cancer

Liver NK cells, characterized by high CD56 expression, constitute approximately 50% of the intrahepatic lymphocyte population. In contrast, NK cells infiltrating liver tumors express memory NK cell markers (KLRC1 and KLRC2—killer cell lectin-like receptors 1 or 2). A high presence of NK cells, along with CD8+ T cells, is associated with better prognosis in early-stage hepatocellular carcinoma (HCC) [407].

Conversely, in late-stage HCC, the percentage of CD56^dim^ NK cells is reduced in both peripheral blood and liver. Intratumoral CD56^dim^ NK cells exhibit PD-1 and NKG2A expression, correlating with poorer prognosis. In these patients, NK cells lose their anti-cancer cytotoxic activity due to an imbalance among NK cell subtypes [264,408].

#### 3.7.6. B Cells in Liver Cancer

A high density of B cells in the tumor zone is associated with better prognosis, reduced tumor size, absence of metastasis, and increased CD8+ T cell infiltration, particularly in HBV-positive HCC patients [409].

In HCC, plasma cells (CD20−CD79a+) are crucial, as their presence correlates with increased Ig secretion and improved prognosis. As in other cancers, B cells in HCC form tertiary lymphoid structures, whose density positively correlates with T and NK cell numbers and activation. Higher TIB density is also linked to increased apoptosis in HCC patients [410,411].

B cells play a dual role in HCC. IgD-IgG+CD27-CD38− atypical B memory cells cooperate with CD8+ T cells, contributing to better prognosis and enhanced anti-cancer effects. Conversely, high intratumoral CD20+ B cell infiltration correlates with poor differentiation and reduced OS in HCV-induced HCC [412]. B cells contribute to humoral immunity and antibody-dependent cellular cytotoxicity (ADCC) against HCV-infected liver cancer cells. Their neutralizing antibodies aid in HCV elimination but may also trigger autoimmunity, exacerbating patient suffering [413].

In HCC patients, B cells produce high levels of IL-10 and express PD-L1, contributing to the anti-cancer immune response. However, in advanced cancer stages, the number of regulatory B cells (Bregs) increases, directly promoting tumor cell proliferation through CD40/CD154 interactions. Interestingly, a distinct population of Bregs with the CD5+CD24−/+CD27+CD38+ phenotype has been identified in humans, showing a positive correlation with the lymph node metastasis [414].

#### 3.7.7. Monocytes and Related Cells in Liver Cancer

HCC patients’ monocytes are recruited to the tumor mass by C-C motif chemokine ligand 2 (CCL2), and different subsets of monocytes appear during the cancer progression. In early stages of HCCs, monocytes are capable of eliminating cancer cells, but liver cancer cells induce monocyte transformation into PD-L1/2+ CD14+ cells. This transformation is correlated with the shortening of the OS. Moreover, CCR1+CD14+ monocytes present in tumor lesions express PD-L1, B7-H3 and TIM-3, and enhance angiogenesis and metastasis. CCL14 appears to be the primary recruiter of CCR1+CD14+ monocytes to the tumor mass, with high CCL14 levels correlating with poor prognosis. Additionally, a distinct population of CD86+HLA-DR+ monocytes has been identified, capable of recruiting neutrophils into the tumor via oncostatin M, a pro-metastatic factor that promotes cancer progression. Elevated oncostatin M levels positively correlate with metastasis in HCC patients [301,310].

In liver cancer, the activation of CD8+ T cells is related to the number of DCs and also CD4+ T cells. In the peripheral blood of patients with HCC, a significant decrease in the number of pDCs and cDCs is observed in comparison to healthy individuals. Nevertheless, the CD303+ pDCs secreting IFN I accumulate within the mass of tumor which is correlated with a poor prognosis. Additionally, there are also SIGN+ infDCs, derived from monocytes, which exhibit characteristics of both DCs and macrophages. Inflammatory DCs (infDCs) promote Th17 differentiation from naive T cells, and their high numbers worsen prognosis [415].

In HCC, CD303+ pDCs contribute to altered antigen presentation to CD8+ T cells. DCs promote HCC progression by inducing tolerance to cancer antigens or suppressing cytotoxic T cell (CTL) activity. Cancer cells, in turn, influence DC maturation via IL-10 or VEGF, while immature DCs facilitate tumor proliferation by inducing antigen tolerance. The interaction between DCs and Tregs plays a key role in generating tolerance to cancer antigens in HCC, correlating with poor prognosis [416,417,418].

In addition, the number of pDCs corresponds to advanced tumor-node-metastasis stage and Th17 infiltrations in patients with HCCs. mDCs in this group of patients highly express HLA class II molecules, whereas HLA class I expression is limited. Interestingly, in HCCs patients, in addition to the classical mDCs subpopulations (cDC1 and cDC2), cDC3 has been identified, which expresses CCL19, lysosomal associated membrane protein 3 (LAMP-3) and CCR7. The LAMP3+ DCs subset shows a high ratio of maturation and migration and strongly corresponds to the number of exhausted T cells [419].

As in other cancers, MDSCs are elevated in the peripheral blood of HCC patients. Their accumulation in the tumor mass correlates with high oncogenic cell cycle-related kinase (CCRK)/IL-6/CD11b/CD33 expression and poor prognosis. Notably, HSCs (hepatic stellate cells) induce mo-MDSC formation from circulating CD14+HLA-DRhigh monocytes. Additionally, MDSCs in HCC fail to stimulate T cell cytotoxicity, leading to worse prognosis and accelerated tumor growth [420,421].

#### 3.7.8. Other Immune Cells in Liver Cancer

The presence of TANs and a high neutrophil-to-lymphocyte ratio are prognostic indicators of poor outcomes in HCC patients. Increased TAN infiltration correlates with enhanced tumor growth, lymph node metastasis, and overall poor prognosis [105,422].

IGF-1 and IGF-2 are involved in the remodeling of macrophages during their maturation. IGF-1 enhances tumor cell proliferation in a macrophage-dependent manner. Elevated levels of TAMs facilitate angiogenesis, cancer cell proliferation, invasion, and metastasis. Additionally, high levels of TAMs have been linked to poor prognosis in HCC patients. M2 macrophages facilitate cancer cell migration in HCC through the TLR4/STAT3 signaling pathway. CXCL8 chemokine produced by activated macrophages has been demonstrated to promote HCC progression and metastasis [423,424,425,426].

### 3.8. Colorectal Cancer

Colorectal cancer (CRC) is the third most common type of cancer and the second most common cause of cancer-related deaths. It is diagnosed more often in males than in females and can affect the colon, anus, or rectum. There is an increasing incidence of this cancer in individuals under the age of 50, which is highly concerning. Approximately 30% of cases are hereditary forms of the disease. Risk factors associated with this cancer include hereditary CRC syndromes, such as *Lynch* syndrome, and inflammatory bowel disease (IBD), which includes ulcerative colitis (UC) and Crohn’s disease, as well as obesity, smoking, alcohol consumption, poor diet, and a sedentary lifestyle [427,428,429,430].

Based on unique genes and signaling pathways, four molecular subgroups of colorectal cancer are distinguished: CMS1 (immune), CMS2 (canonical), CMS3 (metabolic), and CMS4 (mesenchymal). CMS4 is the most aggressive and has the worst prognosis compared to the other subtypes [431,432].

CRC exhibits significant heterogeneity, with the major histological type being adenocarcinoma, which accounts for approximately 95% of all large bowel tumors. At least 17% of large bowel tumors are classified as colloid or mucinous adenocarcinomas, characterized by large amounts of extracellular mucin. Other rare variants of epithelial carcinomas include squamous cell carcinomas and adenosquamous carcinomas (adenoacanthomas). Additionally, undifferentiated carcinomas, including carcinoma simplex, medullary carcinoma, and trabecular carcinoma, are recognized, along with carcinoid tumors and non-epithelial tumors such as leiomyosarcomas, hematopoietic and lymphoid neoplasms, and gastrointestinal stromal tumors (GISTs) [433].

Common therapeutic strategies for colorectal cancer include surgical tumor removal, chemotherapy, and targeted therapy. However, the prognosis for patients with colorectal cancer is usually poor [434].

#### 3.8.1. CAFs in Colorectal Cancer

CAFs in CRC originate from resident fibroblasts, bone marrow cells, and stem cells. There are three subtypes of CAFs in colorectal cancer: myCAFs, iCAFs, and apCAFs [435,436].

CAFs promote colorectal cancer cell survival, proliferation, stemness, and metastatic potential. A high number of CAFs in the TME of colon cancer is associated with a poor prognosis. CAFs promote tumor development by communicating with cancer cells through multiple signaling pathways. CCL2 and CXCL8, secreted by CAFs, induce cancer cell proliferation and invasion. CXCL14 secreted by CAFs stimulates the pro-tumor activity of CAFs and also acts on cancer cells, enhancing their proliferation. IL-6 and IL-11 influence the formation of new CAFs in the TME and promote tumor growth. These chemokines activate STAT3, which is associated with cancer progression and poor prognosis. STAT3 activity in CAFs induces the release of IL-6, TGF-β, and VEGF by cancer cells, promoting immunosuppression and metastasis). TGF-β reduces T cell activity, allowing immune evasion, and interacts with CAF-derived exosomal miR-17-5p, influencing cancer cell invasion and metastasis formation. WNT2 produced by CAFs induces angiogenesis. ADAMs expressed by CAFs affect invasion and metastasis formation [290,292,437,438,439,440,441,442,443].

#### 3.8.2. Other Non-Immune Cells in Colorectal Cancer

TECs can stimulate and activate colorectal cancer stem cells by mediating Notch and Akt signaling [444,445].

Similarly, as in other types of cancers, in CRC there are cancer-associated adipocytes impacting on the cancer cells growth and metastasis. In obese patients, the risk of colon cancer development is increased. It is associated with the inflammation process that contributes to the development of colitis-associated cancers. Moreover, the secretion of TNF-α, a key mediator of obesity-induced inflammation and colon cancer development, is an important biomarker of risk of cancer development [446].

#### 3.8.3. T Cells in Colorectal Cancer

In CRC patients, tumor-infiltrating lymphocytes comprise T, B, and NK cells, macrophages, and other immune cells, serving as a prognostic factor for disease progression and survival. A higher TIL count is associated with better prognosis, while a lower count correlates with metastasis and cancer cells spreading via blood, lymphatic vessels, or the perineural space. Notably, increased CD4+ and CD8+ T cell levels correspond to a lower risk of metastasis, improved prognosis, reduced relapse rates, and longer OS. The positive correlation between T cell abundance and survival is particularly linked to CD8+ T cells. Patients without early metastatic invasion exhibit enhanced expression of cytotoxic T cell-specific genes (CD8α, granzyme B, granulysin) and Th1 markers (T-bet, IFN-γ). Additionally, CD45RO+CCR7- memory T cells are proposed as independent positive prognostic indicators, correlating with better clinical outcomes [447].

In CRC, T cell dysfunction limits the ability to eliminate cancer cells within the TME. This impairment results from defects in T cell activation and cytokine production, particularly IFN-γ and TNF-α. Additionally, Tregs, TAMs, MDSCs, and CAFs further suppress T cell activity in gastric cancer. Notably, microsatellite instability in colon cancer significantly enhances CD8+ T cell infiltration, which correlates with a favorable prognosis [448].

In CRC patients, Tregs are linked to tumor growth inhibition and intestinal inflammation. Increased proinflammatory cytokine release, particularly IFN-γ from various T cell subtypes, NK cells, γδ T cells, and Th1 cells, is associated with longer survival. Additionally, Tγδ (Vδ1) cells, comprising <4.5% of the total leukocyte population, play a crucial role. Their elevated numbers are associated with poor prognosis, as they serve as a primary source of IL-17A. Infiltration of IL-17A-secreting γδ T cells correlates with advanced tumor stages, larger tumor size, lymphatic and vascular invasion, and lymph node metastasis [449,450].

#### 3.8.4. NK Cells in Colorectal Cancer

The number of NK cells in CRC is limited, even if the levels of intratumoral IFN-γ, CCL3, CXCL10 and CXCL12 contributing to the NK cells recruitment to the inflamed tumor area are elevated. NK cells in colon cancers exhibit both pro- and anti-cancer activity. In CRC, the proportion and absolute number of CD56^dim^CD16- NK cells, which regulate immunity by producing cytokines that suppress CD4+ T cell activity, are increased. Conversely, a decrease in CD56^dim^CD16+ NK cells correlates with a weaker anti-cancer immune response. Interestingly, tumor-infiltrating NK cell phenotypes differ based on tumor localization. In left-sided CRC, CD56^bright^ NK cells predominate, correlating with a better response to chemotherapy [451,452].

In CRC, high levels of CD3+CD56+ NK cells are associated with OS. However, it is important to note that CD56+ expression occurs on NK cells and T cells only upon activation [453].

#### 3.8.5. B Cells in Colorectal Cancer

In CRC, CD20+ B cell infiltration into the tumor mass is an independent prognostic factor, with high levels of CD20+ B cells and CD8+ T cells correlating with better prognosis. Similarly, CD138+ plasma cells are associated with improved outcomes. In tertiary lymphoid structures within colon cancer, B cells readily differentiate into plasma cells producing IgG, leading to an increased IgG/IgA ratio. High IgG1 levels correlate with high clonality and hypermutation, enhancing antitumor immunity. However, some studies suggest that B cells may also suppress T cell activity in CRC by releasing cytokines that inhibit T cell function [454,455,456].

#### 3.8.6. Monocytes and Related Cells in Colorectal Cancer

In colorectal cancer (CRC) patients, intermediate monocyte levels are higher in primary tumors than in metastatic site. In these patients, DCs are activated by TAM/CD4 cells and involved in antigen presentation to T cells. The presence of mDCs in these patients correlates with better prognosis and response to treatment strategies, whereas the presence of pDCs correlates with poor prognosis and poor response to the treatment. In CRC patients, tumor-associated dendritic cells (TADCs) represent a specific dendritic cell population whose increased presence in the tumor microenvironment (TME) is linked to poor prognosis. TADCs contribute to immune suppression by inhibiting T cell-mediated responses [457].

Patients with locally advanced CRC exhibit significantly lower infiltration by mature DCs expressing CD83+ on their surface in tumor stroma and margins. In these patients, a lower presence of CD1a+ immature DCs in the stroma and CD83-DCs in invasive margins is commonly associated with a higher frequency of distant metastases [458].

Observations suggest that the extent of dendritic cell (DC) infiltration and prognosis depend on cell maturity. While high intraepithelial infiltration of iDCs tends to correlate with longer survival, the presence of mature DCs exhibit adverse effects on the length of survival (due to their distinct phenotypes and cytokine profiles) [459].

MDSCs modulate the immune response by suppressing immune activity and facilitating cancer progression. In CRC, elevated MDSC levels positively correlate with advanced cancer stage and metastasis [460,461].

#### 3.8.7. Neutrophils in Colorectal Cancer

High levels of TANs are associated with an improved overall survival in patients with stage II CRC. TANs exert an antitumor effect in the early stages of tumor development. In these stages, CD62L and CD54 neutrophils facilitate T cell growth and IFN release. IFN signaling is responsible for antitumor activity of neutrophils, which express high levels of TNF-α, ICAM-1 and CCL3, and low levels of arginase-1 (Arg-1). Neutrophils interact with other immune cells to enhance antitumor responses. In CRC, they colocalize with CD8+ T cells and enhance their responsiveness to T cell receptor signaling, promoting activation and proliferation. However, neutrophil extracellular traps contribute to tumor progression and metastasis. In the CRC microenvironment, NETs, in the presence of tumor-derived IL-8, significantly stimulate CRC cell proliferation, invasion, and migration via TLR9-dependent signaling. TANs further support cancer progression by secreting cytokines and chemokines that enhance tumor cell survival and therapy resistance. For example, TANs release anterior gradient-2 (AGR2), which promotes CRC cell migration through its receptor CD98hc-xCT. Additionally, CXCL2 stimulation increases colon cancer cell proliferation and adhesion via CXCR2 signaling. Clinically, mild chemotherapy-induced neutropenia has been associated with improved therapeutic response and prognosis across multiple tumor types, including CRC [432,462,463,464,465,466,467,468,469].

### 3.9. Lung Cancer

Lung cancer is the second most common type of cancer in both sexes. They constitute a heterogeneous group of cancers classified according to the histological, cellular, molecular and genetic features, and divided into two main groups: small-cell lung cancer (SCLC) and non-small-cell lung cancer (NSCLC). SCLC is a highly malignant neuroendocrine cancer primarily affecting current or former tobacco smokers. It accounts for approximately 15% of all lung cancer cases. Over 60% of patients with SCLC develop metastases in the opposite lung, brain, liver, adrenal glands, or bones. NSCLC accounts for approximately 85% of all lung cancers and includes three histological subtypes: adenocarcinoma, squamous cell carcinoma of the lung, and large cell carcinoma of the lung. Based on the new WHO classification, several types of lung cancers are recognized: epidermoid carcinomas, large cell neuroendocrine carcinomas, adenosquamous carcinomas, sarcomatoid and pleomorphic carcinomas. The composition of the TME in lung cancers impacts on patients survival and their response to standard therapies [470,471,472,473,474,475].

#### 3.9.1. CAFs in Lung Cancer

In lung cancer, CAFs originate from normal resident fibroblasts, mesenchymal stem cells, adipocytes, pericytes, epithelial cells and smooth muscle cells. The main subtypes of CAFs in lung cancer microenvironment are: myCAFs, iCAFs and apCAFs. CAFs in lung cancer can be divided into different subsets: CAF-S1, CAF-S2, CAF-S3, CAF-S4 and CAF-S5 (Figure 4). They express fibroblasts activation markers on different levels [476,477].

CAFs influence the invasion and metastasis of primary lung tumors by participating in ECM degradation and promoting EMT. CAFs promote EMT in lung cancer cells by delivering Snail-1 proteins and microRNAs miR-210 and miR-224 through exosomes, as well as by secreting IL-6 and TGF-β IL-6 plays a role in the interactions between CAFs and cancer cells in lung cancer. TGF-β, produced by cancer cells, increases IL-6 expression in CAFs, which in turn influences EMT. IL-6 also affects the metastatic potential of lung cancer cells by activating the JAK2/STAT3 signaling pathway, which impacts angiogenesis by increasing the expression of VEGF and bFGF [267,445,477,478,479,480,481,482,483].

CAFs influence the immune response in lung cancer. They express ligands of the PD1 receptor, including PDL1 and PDL2, and inhibit T cell functions. CAFs can also cross-present antigens in complex with MHC I to antigen-specific CD8+ T cells, that results in antigen-specific upregulation of PDL2 and FAS ligand (FASL) in T lymphocytes, ultimately leading to their elimination [484].

CAFs inhibit the response of lung cancer cells to chemotherapy through several signaling pathways. CAFs can modulate the response to cisplatin-based chemotherapy by activating the Gas6/AXL, IL-11/IL-11R/STAT3, SDF-1/CXCR4/NF-κB/Bcl-xL, SRGN/CD44, ANXA3/JNK, IGF2/AKT/Sox2/Pgp, and PI3K/Akt/GRP78 signaling pathways. Gas6 protein derived from CAFs has a high affinity for the AXL tyrosine kinase receptor, whose levels increase during cisplatin-based chemotherapy. AXL activation is associated with EMT and promotes cancer cell survival, chemotherapy resistance, invasion, and metastasis formation. IL-11 affects the resistance of lung cancer cells to cisplatin. IL-11 releases results in increased expression of the anti-apoptotic protein Bcl-2, enabling cells to evade apoptosis. SDF-1 release by CAFs promotes cell proliferation and induces resistance to cisplatin by increasing the expression of CXCR4, NF-κB, and Bcl-xL. SRGN influences chemoresistance and also induces cancer cell stemness by promoting Nanog induction. Increased ANXA3 expression in CAFs activates c-Jun N-terminal kinase (JNK), which inhibits cisplatin-induced apoptosis. IGF2 produced by CAFs binds to the IGF1 receptor in lung cancer cells and activates the AKT/Sox2 pathway, leading to increased expression of P-glycoprotein, which can pump out chemotherapeutic agents reaching the tumor. CAFs-derived HGF inhibits paclitaxel-induced apoptosis of lung cancer cells by increasing the expression of the PI3K/Akt pathway and glucose-regulated protein 78 (GRP78), which supports cancer cell survival and contributes to chemoresistance [485,486,487,488,489,490,491,492].

#### 3.9.2. Endothelial Cells in Lung Cancer

The proliferation and migration of endothelial cells enables the formation of new blood vessels, which is crucial for tumor progression and metastasis. Lung cancer cells activate endothelial cells by releasing factors such as VEGF. In turn, activated endothelial cells facilitate tumor metastasis through epigenetic mechanisms. It has been shown that microRNAs derived from TECs promote metastasis formation. VEGF reduces the expression of miR-1 in TECs, playing a significant role in tumor progression and angiogenesis. miR-143/145 expressed in TECs promotes angiogenesis and impacts tumor expansion [493,494,495,496].

#### 3.9.3. Adipocytes in Lung Cancer

While the connection between lung cancer cells and adipocytes remains unclear, preclinical studies in murine models of lung cancer have shown that obesity increases mortality in mice with this cancer [497].

It is worth noting that a common site of lung cancer metastasis is bone. With increasing numbers of adipocytes from bone marrow in the TME the potential for lung cancer cells to metastasize into the bones increases. The expression of the S100A8/A9 (heterodimeric calcium-binding protein) released by bone marrow adipocytes is a predictive factor of bone tropism. Interaction between these adipocytes and lung cancer cells increase secretion of IL-6 by the former cells, which enhances inflammation during cancer development [498,499].

#### 3.9.4. CSCs in Lung Cancer

CSCs are typically isolated based on the presence of specific molecules on their surface or within the cells, frequently belonging to the cluster of differentiation (CD) group. A substantial body of evidence has validated the presence of the following markers on lung CSCs: CD133, CD44, and CD90. CD133 is one of the most frequently used markers of lung CSC. High levels of CD133 expression significantly correlate with the occurrence of low-grade lymph node metastases. CD44 plays a pivotal role in a multitude of signaling processes, including cell differentiation, survival, apoptosis, migration, and proliferation. Recent research has demonstrated that CD44 is a crucial factor for CSCs, self-renewal, resistance to apoptosis, and preparation of the niche. CD90 plays a role in cell–matrix and cell–cell interactions. Although CD90 has been identified as a marker of various types of CSCs, its role as a marker of CSCs in the lung has yet to be fully elucidated. It has been demonstrated that lung cancer stem cells co-expressing CD44 and CD90 can be identified in primary lung cell cultures. Thus far, no mutations that activate CD90 expression have been identified. The results of studies conducted on mouse models indicate that DNA methylation may be a factor in the promotion of CD90 expression [474,500,501,502,503].

#### 3.9.5. T Cells in Lung Cancer

The NSCLCs are characterized by the high levels of somatic non-synonymous mutations described as a tumor mutation burden (TMB) that are presented as the neoantigens to the T cells. The TILs presence in lung cancers is an independent prognostic factor for patients survival. Intensive infiltration of the TME by T cells correlates with better prognosis and longer survival in patients with lung cancers. Among TILs, CD8+PD-L1+ T cells are associated with the better response to the therapy based on the PD-1/PDL-1 axis. In general, the high number of the CD4+ and CD8+ T cells in tumor stroma indicates a better prognosis; however, the presence of high numbers of CD8+ T cells in NSCLC patients after resection could be considered as the additional factor in the classification involving tumor-node-metastasis [504,505].

In patients with the advanced stage of NSCLC, CD8+ TILs are dysfunctional and show a high proliferation rate and high expression of activation markers. The number of cells with high expression of PD-1, mucin domain-containing protein 3 (TIM-3) and LAG-3 (CD223) is associated with resistance to immunotherapy. At the same time, Tregs expressing the lymphocyte-activation gene 3 (LAG-3) are more active in lung cancers than those not expressing it. In contrast, CTLs expressing LAG-3 show decreased activity and proliferation [506,507].

The number and activity of Tregs in lung cancer are associated with poor clinical outcomes, and an increase in PD1+ Tregs populations could be observed in patients with NSCLCs resistant to PD-1/PDL-1 therapy. Another important factor to consider is the balance between PD1+CD8+ T cells and PD1+Tregs in the TME of patients with NSCLCs. Evaluation of this ratio allows predict the clinical efficacy of immune checkpoint inhibitors, because the density of PD-1+ Tregs is considered a predictive biomarker of response to that treatment [460].

#### 3.9.6. NK Cells in Lung Cancer

In response to cancer or infection, the NK cells normally circulating in the peripheral blood are attracted to the lungs, where they form infiltrations in stroma. Interestingly, in murine model of lung cancer with Kras mutation, their number decreases almost 6-times during the cancer development [508].

Most tumor-infiltrating NK cells exhibit a CD56brightCD16- phenotype and likely express CXCR3, since CD16- NK cells typically express this marker. Given that human lung cancers highly express CXCR3 ligands, they are thought to attract CD16- NK cells. In murine models of lung cancers, these NK cells demonstrate low glycolytic rates, and expression of fructose 1,6-bisphosphatase (FBP1), that diminishes glycolysis in NK cells. This leads to a decrease in their cytotoxic activity in lung cancer [509,510,511].

NK cells in NSCLC patients show high expression of TIM-3 which correlates with decreased cytotoxic activity of NK cells and poor prognosis. At the same time, decreased expression of the NKp30, NKp80, DNAM1 (PTA1), CD16 and ILT2 receptors observed in NK cells in these patients, can also influence the clinical outcomes [512,513].

NK cells secrete perforins, granzymes, and IFN-γ, which inhibit lung cancer growth. However, in vivo studies show that lung NK cells respond poorly to activation, even less than those in peripheral blood, likely due to interactions with alveolar macrophages and soluble factors in the lower respiratory tract [514].

After lung resection, NK cells release less granzyme B, likely due to soluble factors from lung cancer cells. Perforins and IFN-γ levels are also reduced. Additionally, TAMs and MDSCs suppress NK cell function by releasing IL-10 and TGF-β, correlating with lower survival rates in lung cancer patients [515].

#### 3.9.7. B Cells in Lung Cancer

In human models of NSCLC, tumor-infiltrating lymphocytes B are detected in the tumor mass. The number of TILBs positively correlates with OS ratio. TIBs are present in both early and advanced stages of lung cancer. Their localization is regulated by signals from the TME. B cell-enriched areas include germinal center cores and mantle regions. In the former, B cells differentiate into plasma cells, which play a key role in the anti-cancer response. Plasma cells in lung tissue produce antibodies against cancer antigens (e.g., LAGE-1, TP53, and NY-ESO-1), with higher antibody levels observed in lung cancer patients than in healthy individuals [516].

Activated TILBs with the phenotype CD19+CD20+CD69+CD27+CD21+ secrete IFN-γ, whereas TILBs with the phenotype CD19+CD20+CD69+CD27-CD21- are associated with the Bregs. The latter B cells are still able to present antigens but only for a short time [516].

B cells contribute to the promotion of T cell-mediated responses. High levels of CD4+ and CD8+ T cells, along with CD20+ B cell infiltration in the tumor mass, are associated with longer survival in NSCLC patients. A similar correlation between CD20+ B cells and CD8+ T cells has been observed in large cell lung cancer (LCC).

Moreover, the CD20+ B cells infiltration corresponds with the smoking status of patients and their number and activity is higher in non-smokers with NSCLC as compared to smokers with NSCLC; however, in the large cell carcinomas, high degree of the CD20+ B cell infiltration corresponds with longer survival. In general, a low number of follicular B cells and mDCs is associated with a higher risk of poor survival.

In NSCLC patients, the number of CD69+HLA-DR+CD27+CD21+ B cells correlates with CD4+ T cells releasing IFN-γ. Increased follicular B cell density in lung tumors is associated with higher CD4+ and CD8+ T cell receptor (TCR) clonality. Conversely, a low number of follicular B cells or myeloid dendritic cells (mDCs) correlates with a higher risk of poor survival [517].

Regulatory B cells (Bregs) in lung tumors polarize towards IL-10-secreting B cells, a response to inflammation. In lung cancer models stimulated by LPS, B cells also polarize towards Bregs, which promote tumor progression in experimental models. Although most evidence comes from murine studies, human lung cancer patients show higher levels of CD24+IL-10+ B cells than normal lung tissue. Additionally, IL-10+ B cell levels are elevated in patients with high numbers of Tregs and MDSCs in advanced disease stages, suggesting a similar role for Bregs in human and murine models [518].

#### 3.9.8. Monocytes and Related Cells in Lung Cancer

In NSCLC patients, a higher proportion of the classical and intermediate monocytes is observed in comparison to the healthy individuals. The effects are enhanced in patients with smoking and drinking history and in patients with metastatic lesions in liver [519].

DCs found in the tertiary lymphoid structures are considered as a strong independent positive prognostic factor, in the context of survival. DCs regulate T cell polarization in the lungs. They infiltrate lung tissue, with peritumoral DCs showing increased CD11b expression, facilitating CD4+ T cell activation. Another DC population, characterized by high CD141 expression, primarily interacts with CD8+ T cells. However, the proportion of CD141+ DCs is lower in lung cancer patients than in healthy individuals. In these patients, pDCs are associated with a worse prognosis due to their impaired type I IFN secretion, which is crucial for cytotoxicity, immune suppression, and antitumor responses. pDCs are more abundant in patients with rapidly proliferating cancer cells and reach higher levels in advanced-stage NSCLC. They may contribute to the immunosuppressive properties of the lung cancer TME, promoting disease progression [520,521].

In lung cancer, classical dendritic cells with a regulatory phenotype (regDCs) are present alongside pDCs. These cells can be divided into GR1+ and GR1-regDCs, which primarily inhibit tumor growth while also facilitating cancer progression. They are most prevalent in the early stages of NSCLC, with cDC1 playing a key role in immune control and response to immunotherapy [522].

The number and phenotype of DCs in lung cancer influence patient survival after treatment with atezolizumab (PD-L1 blockade). This correlates with the high expression of PD-L1 on lung cancer cells, aiding in the selection of NSCLC patients for monotherapy with checkpoint inhibitors. In the immunogenic early stages of lung cancer, the tumor mass is infiltrated by mDCs, CD4+, and CD8+ T cells, whereas in later stages, it is dominated by MDSCs, TAMs, Tregs, and CAFs [523].

In patients with the NSCLCs, MDSCs similar to Tregs express CD39 and CD73 markers that are capable of converting ATP into adenosine, an important mediator of immune suppression. The presence of these MDSCs positively correlates with the disease progression, but chemotherapy can limit their number. The complete role of the MDSCs in the lung cancer development and sensitivity to treatment is still being studied, but they have been implicated in the lung cancer metastasis and inhibition of their activity may overcome resistance to existing treatments [524,525].

#### 3.9.9. Other Immune Cells in Lung Cancer

A substantial body of evidence from numerous studies indicates that neutrophils play a pivotal role in tumor development. They facilitate tumor growth by degrading the extracellular matrix, suppressing the immune system, stimulating tumor cell proliferation, enhancing metastatic potential and promoting angiogenesis. Furthermore, clinical studies have demonstrated that patients with lung cancer exhibit elevated levels of neutrophils in both the tumor and the bloodstream. A higher neutrophil count is associated with an increased risk of recurrence following surgery, a poorer response to treatment, and a poorer prognosis in patients with lung cancer, compared to those with lower neutrophil levels [526,527,528].

In lung cancer, a high density of M2 TAM infiltrates in the tumor stroma is associated with a number of factors, including tumor differentiation, growth, invasion, lymph node metastasis, pathological stage and poor prognosis. In contrast, M1 macrophages are typically situated within tumor islands and are associated with a more favorable prognosis [529,530].

### 3.10. Breast Cancer

Breast cancers are the most common malignancies in women and in most cases arise from the normal breast ducts. Generally, they are a result of an accumulation of mutational changes in the duct epithelium. Progression from normal breast tissue to breast malignancy is usually accompanied by qualitative and quantitative changes in the function and localization of the immune cells in the stroma and parenchyma [531].

Breast cancers are categorized into invasive and non-invasive types. The two most common types of invasive breast cancer, invasive ductal carcinoma (IDC) and invasive lobular carcinoma (ILC), are defined by their site of origin. Breast cancer can also occur in men, though it is very rare and accounts for only 1% of all breast cancers. Most male breast cancers are invasive ductal carcinomas [532].

According to the molecular and histological features BC could be divided into the three groups: BCs expressing hormone receptor (ER—estrogen receptor), or (PR—progesterone receptor), BCs expressing HER+ (human epidermal receptor 2) and TNBC (triple-negative breast cancers). Furthermore, the TNBC could be further divided into: basal-like 1 (BL-1), basal-like 2 (BL-2), immunomodulatory (IM), mesenchymal (M), mesenchymal stem-cell-like (MSL) and luminal androgen receptor (LAR). Among them, the BCs expressing HER are the most common. The most important factors influencing the BCs development are: obesity, alcohol consumption, sedentary lifestyle, exposure to high levels of exogenous (contraceptive pills or hormone replacement therapy administration) and endogenous (early menarche/late menopause and late pregnancy) hormones, exposure to the radiation, old age and mutations in several genes (e.g., in *BRCA1*, *BRCA2*, *PALB2*, *TP53*, *PTEN*, *STK11*, and *NF1*) [531,533].

The microenvironment in breast cancer could be considered at three different levels: local, regional or distant (metastasis related). The composition of the breast stroma influences breast tissue density and plays a role in the BCs development. Among others, immune cells (T CD4+, CD8+, B cells, dendritic cells, macrophages, NK cells) are considered a factor playing a critical role in breast carcinogenesis [534].

#### 3.10.1. CAFs in Breast Cancer

CAFs are an extremely heterogeneous group of cells, but in breast cancer, the sources of CAFs are limited. In breast cancer, CAFs usually originate from normal fibroblasts, MSCs, adipocytes, and pericytes. CAFs exhibit varying levels of expression of different molecules depending on the molecular subtype of breast cancer. It has been shown that CAFs from HER2-positive breast cancer exhibit significant differences compared to triple-negative and ER-positive breast cancers. Notable differences have been observed in the levels of genes related to the cytoskeleton and integrin signaling, which are associated with increased migration and poor prognosis [535,536,537].

CAFs influence the development of breast cancer by enabling cancer cells to acquire a malignant phenotype through communication via various factors. The interaction of CAFs with breast cancer cells results in excessive proliferation, increased permeability of the endothelial cell layer, and the formation of metastases. Fibroblasts transferred from primary tumors increase the efficiency of metastasis formation in the distant organs [538].

CAFs promote EMT and increase breast cancer cell motility through secretion of TGF-β, which enhances the expression of fibronectin, vimentin, MMP2, MMP9, Snail, and TWIST. TGF-β also contributes to therapy resistance and drives breast cancer cell stemness by increasing the expression of the lncRNA HOX antisense intergenic RNA (HOTAIR), which silences tumor suppressor gene expression in breast cancer cells. FGF5 secreted by activated CAFs also supports breast cancer cell stemness and therapy resistance. FGF5 activates HER2 through the FGF receptor (FGFR)2/c-Src/HER2 axis, leading to resistance to HER2-targeted therapies, which can be reversed by using FGFR inhibitors. FGF2 produced by CAFs promotes cancer cell growth and migration through interaction with FGFR1. IL-6 from CAFs affects breast cancer cell invasiveness and contributes to apoptosis resistance. IL-32, expressed by CAFs, binds to integrin β3 via an RGD motif, activating downstream intracellular p38 mitogen-activated protein kinase (MAPK) signaling, which increases the expression of EMT markers and promotes cancer cell invasion. Tenascin-C secreted by CAFs increases breast cancer cell resistance to apoptosis, thereby contributing to the formation of metastatic foci. Osteopontin, gremlin-1, and collagen triple helix repeat-containing 1 (CTHRC1) influence migration, invasion, and EMT in breast cancer cells [539,540,541,542,543,544,545,546,547].

The reverse Warburg effect is an important mechanism promoting breast cancer progression. The loss of Cav-1 in CAFs, occurring through autophagy, leads to mitochondrial dysfunction, oxidative stress, and enhanced aerobic glycolysis. This results in increased production of lactate, pyruvate, glutamine, and ketones, which fuel cancer cell metabolism and enable tumor growth. Cancer cells reprogram CAFs from oxidative metabolism to glycolytic metabolism by activating the estrogen receptor (GPER)/cyclic adenosine monophosphate (cAMP)/protein kinase A (PKA)/cAMP response element-binding protein (CREB) signaling pathway. Glycolytic CAFs, in addition to producing nutrients for cancer cells, increase mitochondrial activity in cancer cells and make them therapy resistant. Exosomal miR-105, derived from breast cancer cells expressing MYC and the HMGB1 protein can also reprogram CAFs [343,548,549,550,551].

CAFs play a crucial role in suppressing the immune response in breast cancer. elimination of CAFs increases the level of IL-2, IL-7, the number of dendritic and cytotoxic T cells with antitumor functions, and decreases the level of IL-4, IL-6, VEGF, the number of pro-tumor macrophages, and immunosuppressive T lymphocytes [552].

CAFs promote angiogenesis in breast cancer through both VEGF-dependent and VEGF-independent mechanisms. Under hypoxic conditions, there is increased expression of VEGF and promotion of angiogenesis via the HIF-1α/GPER signaling pathway. The secretion of VEGF from CAFs can be induced by IL-6. This process can be halted by the use of tocilizumab, an IL-6 receptor inhibitor [544,553,554].

#### 3.10.2. Adipocytes in Breast Cancer

In non-lactating breasts, adipose tissue comprises 50–60% of the total mass, primarily consisting of white adipose tissue (WAT). Epithelial cells and adipocytes interact closely under both normal and pathological conditions. In breast cancer, this interaction plays a key role in cancer initiation, progression, and metastasis. Adipocytes influence metastasis in breast cancer (BC). They promote this process by releasing leptin, which enhances the expression of metastasis-related genes (e.g., *SERPINE-1*, *MMP2*, and *IL-6*). Additionally, adipocytes in primary tumors, bone, and bone marrow can direct metastasis. Bone is a common site of secondary lesions in BC, likely due to its high adipocyte content [555].

Breast adipocytes could be divided into three subtypes: adipose-derived stem cells (ADSCs), preadipocytes and mature adipocytes. It has also been suggested that a special type of adipocyte may be found in the matrix of invasive breast cancers. The cancer-associated adipocytes (CAAs) display fibroblast-like phenotype, smaller size, small, dispersed lipid droplets and very low expression of the adiponectin and other adipokines. They contribute to promoting metastasis [556].

CAAs occur in the peripheral area of the primary tumor mass, and their large number is associated with a poor prognosis. In vitro, co-culture of breast cancer cells with mature adipocytes leads to increased invasiveness. In general, upon co-culturing adipocytes lose their lipid content and show a decrease in adipocyte markers and an overexpression of cytokines/chemokines involved in the inflammatory process. Breast cancer cells surrounded by adipose tissue promote F-actin remodeling, migration, invasiveness and spheroid organization in in vitro conditions. In turn, in vivo, the cancer-associated adipose tissue secretes oncostatin M (OSM) contributing to the breast cancer progression. Furthermore, adipokines (e.g., adiponectin) stimulate the growth of breast cancer cells and enhance their survival [372,557,558,559].

#### 3.10.3. Other Non-Immune Cells in Breast Cancer

In breast cancer, a subset of tumor-associated macrophages expresses podoplanin (PDPN), which interacts with Gal-8 on lymphatic ECs, promoting the formation of new lymphatic vessels and the migration of cancer cells through the lymphatic system. Studies in a mouse model have shown that the removal of PDPN-expressing macrophages or inhibition of Gal-8 reduces metastatic potential in breast cancer [560].

CSCs in breast cancer demonstrate a high degree of plasticity, which enables them to adapt to alterations in the tumor microenvironment and to evade destruction by therapeutic agents. Such cells have been observed to survive even the most intensive forms of treatment, thereby contributing to the recurrence of the disease. Additionally, CSCs contribute to tumor heterogeneity, which further complicates treatment [561].

#### 3.10.4. T Cells in Breast Cancer

Similarly, as in other cancers, the large number of TILs in BC correlates with a better prognosis and increased likelihood of positive response to neoadjuvant chemotherapy, regardless of molecular subtypes. TILs in breast cancers can be subdivided into sTILs (stromal) and iTILs (intratumoral) T cells [562,563,564].

In TNBC patients, a higher number of CD8+ T cells and CD4+ T cells infiltrating tumor mass is observed, and the number of B and NK cells is higher than in patients with other BC subtypes. Increased CD8+ T cell levels are associated with ER- and PR- subtypes, as well as with favorable prognosis. Similarly, in the HER+ subtype, the presence of TILs correlates with a favorable prognosis, regardless of ER status. In contrast, ER+ BC tumors display low TIL counts, which corresponds to a poor prognosis [562,565].

In BC patients, the large clusters of Tregs are observed in the infiltrate and the number of Tregs corresponds to a high tumor grade, positive lymph node status and short OS. Tregs dominate in TNBC and HER+ subtypes of BC. An increased percentage of Tregs in HER+ is associated with its overexpression and limited OS, whereas the high ratio of CD8+ T cells to Tregs correlates with better prognosis. In turn, in the ER+ type of BC a high number of Tregs is associated with lower ER expression and, consequently, with a better prognosis [566,567].

#### 3.10.5. NK Cells in Breast Cancer

Breast cancer cells can suppress NK cell cytotoxicity through the secretion of estrogen and cytokines/chemokines. In TNBCs, high NK cell numbers are associated with poor prognosis, likely because CD56^bright^CD16^dim^ NK cells express VEGF, which influences angiogenesis and immunosuppression. In HER2+ breast cancers, a higher NK cell count is linked to better prognosis. In ER+ subtypes, a higher NK cell count negatively correlates with ER expression, but increased NK cell infiltration is associated with improved prognosis [568].

In chemotherapy-resistant BC, regardless of subtype, NK cell numbers are similar to those in healthy individuals, but the expression of genes associated with antibody-dependent cellular cytotoxicity (ADCC) is decreased. Despite similar NK cell numbers in healthy controls and women with BC, NK cells in the latter group exhibit a different phenotype (CD56^bright^). Moreover, in this group, NK cells show lower expression of activating receptors (NKp30, NKG2D, NKp46, DNAM1, CD16) and higher expression of the inhibitory receptor NKG2A, which correlates with impaired cytotoxic activity. Additionally, in BC, inactivation of glycogen synthase kinase-3 (GSK3β) is linked to NK cell dysfunction and metastasis [569,570].

#### 3.10.6. B Cells in Breast Cancer

In BC patients, B cells form the tertiary lymphoid structures mainly in the cancer stroma and are associated with high-grade tumors. In addition, in TNBC types of BC, TLSs are found in higher numbers in comparison to other BC subtypes, and they consist of more memory B cells than naïve or plasma cells [571].

In BC patients, the number of B cells with the CD19+PD-1/PD-L1+ phenotype is slightly increased in the TME, along with the number of MDSCs. Moreover, the number of IL-10+CD19+CD24+CD38+PDL1+ Bregs correlates with the Tregs proliferation ratio and with the inhibition of PD-1-positive effector T cells in patients with invasive BC. In contrast, in murine models of breast cancers, Bregs facilitate metastasis by releasing TGF-β, mainly after activation by lipoxygenase. In addition, B lymphocyte-induced maturation protein-1 (BIMP-1), a key regulator of antibody-producing B cell development and function, controls gene expression associated with the transition of B cells to plasmablasts, facilitating the invasion of breast cancer cells into new sites [572,573].

#### 3.10.7. Monocytes and Related Cells in Breast Cancer

In BC patients, a higher number of monocytes expressing CD163 on their surface is observed as compared to healthy women. Typically, the expansion of non-classical monocytes characterizes patients with breast and endometrial cancers [574].

In breast cancers, circulating DCs dominate as compared to the healthy controls. Among them iDCs (immature dendritic cells) are the most abundant and are associated with impaired ability of the immune system to stimulate antitumor activity. In this cancer, large amounts of IL-10 released by cells surrounding the tumor inhibit IL-12 production by cDC1 and suppress the T cell response, promoting cancer development and metastasis. Additionally, cancer-associated pDCs have impaired type I IFN secretion, which supports Treg proliferation [575,576,577].

In HER2+ BC, pDCs predominate, whereas in ER+ subtypes, mDCs are more abundant. In advanced BC, circulating pDC levels were low; however, their presence correlates with better prognosis. In TNBC, a high abundance of intratumoral and stromal immature pDCs is observed [578].

BC cells release G-CSF, IL-6, and TGF-β, promoting MDSC expansion and activation within the tumor. In TNBC, MDSC levels are elevated, and the aggressive subtype is characterized by increased ΔNp63 expression. High ΔNp63 levels correlate with reduced metastasis-free survival. Studies suggest that ΔNp63 promotes cancer progression and metastasis by recruiting MDSCs and T cells to the TME [579].

Interestingly, the level of circulating MDSCs is higher in metastatic than in non-metastatic BC, and the increased numbers of MDSCs correlate with the aggressive cancer course and shortened OS [372,580,581].

#### 3.10.8. Other Immune Cells in Breast Cancer

Neutrophils are a critical component of the TME, with the ability to enter tumors and contribute to their progression. The phenotypic and functional changes that neutrophils undergo in the complex TME provide support for tumor growth in breast and other cancers [582,583].

M1 macrophages play a pivotal role in antitumor immunity by recognizing and phagocytosing breast cancer cells. This activity is accompanied by the production of proinflammatory cytokines, including interferon-γ (IFN-γ) and interleukin-12 (IL-12). These factors facilitate the immune response, which ultimately results in the eradication of cancer cells. However, as tumors progress, M2 macrophages increase while M1 macrophages decrease, leading to a dominant M2 phenotype in the tumor microenvironment. M2 macrophages are regarded as “tumor promoters” due to their capacity to facilitate invasion, metastasis and angiogenesis. They also promote cancer cell stemness, regulate energy metabolism, and enable tumor evasion of the immune response [584,585].

### 3.11. Ovarian Cancer

Ovarian cancer (OC) is the second most common cancer in women over 40, after breast cancers. In more than 60% of cases, OC is diagnosed at a metastatic stage. The prognosis for patients with this cancer is related to the stage at the time of diagnosis: in early stages, the 5-year survival rate is 90%, while in late, metastatic stages, it is approximately 25% [586].

OC can be subdivided into three main types: epithelial, germ cell, and sex-cord-stromal. Moreover, there are four primary histologic subtypes of epithelial ovarian cancers: serous, endometrioid, mucinous, and clear cell. Among these, the most common is serous OC, which is derived from the surface of the ovary or the fimbriated end of the fallopian tube. Primarily, the metastasis of ovarian cancer occurs in the omentum, which is a large fat pad extending from the stomach to the bowel. Invasion of the omentum by cancer results in the transformation of this soft tissue, composed mainly of adipocytes, into a solid tumor devoid of adipocytes [587,588].

Ovarian cancer can occur sporadically in any woman, but certain factors increase the risk. Key contributors include genetic predispositions (e.g., *BRCA1/BRCA2* and *MMR* gene mutations, or *Lynch* syndrome), frequent ovulatory cycles (likely due to pro-inflammatory responses in the distal fallopian tubes), endometriosis, poor dietary factors (e.g., low fiber and vitamin D levels), and ethnicity [589].

TME significantly influences ovarian cancer progression and treatment resistance. In OC, the TME includes ascitic fluid and solid omental niches, each with distinct immune and non-immune cell populations. The omentum contains vascularized immune structures called milky spots, which regulate cancer colonization. These spots house B cells, T cells, NK cells, TAMs, and neutrophils [590,591].

#### 3.11.1. CAFs in Ovarian Cancer

The sources of CAFs in OC are primarily normal fibroblasts, epithelial cells, endothelial cells, smooth muscle cells, adipocytes, and bone marrow MSCs. In this cancer, four CAFs populations have been identified: CAF-S1, CAF-S2, CAF-S3, and CAF-S4 (Figure 4). This classification is based on the differential expression of markers such as α-SMA, FAP, FSP1, CAV1, CD29, and PDGFR-β. CAF-S2 and CAF-S3 resemble normal fibroblasts, whereas CAF-S1 and CAF-S4 are found only in cancerous and metastatic sites. Different types of CAF may correlate with various subtypes of OC and can significantly impact tumor development and treatment outcomes. In the high-grade serous ovarian cancer (HGSOC) subtype, a high concentration of CAF-S1 fibroblasts has been correlated with poor patient survival. CAF-S1 performs immunosuppressive functions by recruiting CD25+FOXP3+ T cells. Within the CAF-S1 population, there are different subtypes such as myCAF and iCAF. MyCAF, characterized by high α-SMA expression, is located near cancer cells, whereas iCAF, which has low α-SMA expression, is located farther from cancer cells. myCAF produces a dense matrix that positively influences tumorigenesis. iCAF secrete inflammatory cytokines, inducing an immunosuppressive environment and activating the JAK/STAT pathway, which promotes tumor growth and contributes to therapeutic failure [27,592,593,594,595].

myCAFs produce TGF-β, which regulates innate and adaptive immunity through its inhibitory effects on NK cells, CD4+/CD8+ T cells, macrophages, and other effector immune cells. TGF-β can reduce the expression of MHC class I in OC. This can be reversed to pre-treatment levels using the TGF-β inhibitor Galunisertib. TGF-β is also a crucial mediator in regulating interactions between cancer cells and the stroma, promoting tumor growth and immunosuppression. Many TGF-β signaling-related genes expressed by CAFs play important roles in the communication between fibroblasts and OC cells. Proteins such as periostin (POSTN), versican (VCAN), collagen type XI alpha 1 (COL11A1), and collagen triple helix repeat containing-1 (CTHRC1), through TGF-β signaling, participate in CAF activation and promote tumor progression. Upregulated CTHRC1 enhances tumor invasion and migration via the EGFR/ERK1/2/AKT pathway and also influences immune response and angiogenesis. Increased COL11A1 expression, through the regulation of TGF-β3, activates CAFs via the NF-κB/IGFBP2 axis and also affects tumor aggressiveness and therapy resistance through the TGF-β1/MMP3 axis. POSTN activates the PI3K/Akt pathway via TGF-β1, affecting migration and invasion. Increased VCAN expression promotes cell motility and invasiveness through the activation of the NF-κB pathway and elevated expression of CD44, MMP9, and the hyaluronan-mediated motility receptor [78,308,592,596,597,598,599,600].

Hepatocyte growth factor (HGF) supports the proliferation of OC cell lines SKOV3 and HO-8910 by activating the c-Met/PI3K/Akt and GRP78 pathways. HGF can also inhibit the effectiveness of paclitaxel, thus complicating treatment. FGF-1 influences tumor progression through the phosphorylation of FGF-4, activation of the MAPK/ERK pathway, and increased expression of EMT-related genes such as Snail-1 and MMP3. The FGF-1 and FGF-4 axis appear to be crucial in OC and may represent a significant strategy for OC treatment [601,602].

In HGSOC, ascitic tumor cells with high expression of integrin α5 (ITGA5) are recruited by CAFs to form heterotypic spheroids, known as metastatic units (MUs). ITGA5 is essential for the formation of MUs. Therefore, CAFs secreting epidermal growth factor (EGF) maintain ITGA5 expression and facilitate maintenance of MUs. MUs centered around CAFs can promote the spread of HGSOC in the peritoneum and accelerate the development of ascites. iCAFs, along with cancer cells, activate the JAK/STAT pathway, influencing the ascites system, promoting tumor growth, and ensuring treatment resistance [595,603].

OC with low immune reactivity is characterized by the lowest levels of interferon and inflammatory response, as well as infiltration by non-T cells, and high activity of MYC and WNT/β-catenin signaling pathways, which are associated with immune exclusion—a phenomenon where the TME prevents immune cells from effectively infiltrating and attacking cancer cells. Additionally, the WNT/β-catenin pathway plays a significant role in resistance to combined anti-PD-L1 and anti-CTLA4 therapy [604,605,606].

The mesenchymal subtype of HGSOC is characterized by a high number of CAF-S1 fibroblasts, which are the main cellular source of CXCL12, regulated by miR200. CXCL12β accumulates in CAF-S1 and exerts immunosuppressive functions through CD25+ FOXP3+ T cells (Tregs). CXCR2 ligands, including CXCL1 and CXCL2, promote MDSC infiltration in cells expressing Snail and lead to OC progression [593,607].

CAFs participate in the metabolic processes of OC through cytokines, metabolites, extracellular vesicles containing metabolites, and TGF-β. Autophagy also plays a significant role in metabolic changes. Studies have shown that in co-cultures of OC cells and fibroblasts, the activation of p38αMAPK kinase in CAFs and phosphoglucomutase 1, involved in glycogen metabolism, can provide a pathway for glycolysis and supply energy for cancer cell proliferation, invasion, and metastasis. Conversely, reduced levels of p38αMAPK and activation of glycogen phosphorylase can inhibit cancer metastasis. CAFs with high CXCL14 expression can increase the expression of long non-coding RNA LINC00092, which affects glycolysis by binding to 6-phosphofructo-2-kinase/fructose-2,6-bisphosphatase 2 (PFKFB2) and promoting cancer metastasis with the support of surrounding CAFs. Low expression of focal adhesion kinase (FAK) in CAFs is associated with reduced overall survival, possibly through the promotion of glycolysis and cancer cell growth. OC cells can induce upregulation of MCT4 and loss of Cav-1 expression through autophagy, resulting in the creation of a microenvironment conducive to tumor growth. Disrupting the metabolic cross-talk between OC cells and fibroblasts may be a promising strategy to prevent OC progression and recurrence [608,609,610].

#### 3.11.2. Adipocytes in Ovarian Cancer

IL-6, IL-8, monocyte chemoattractant protein-1 (MCP-1), tissue inhibitor of metaloproteinase-1 (TIMP-1) and adiponectin released by adipocytes are associated with the ovarian cancer cells homing in the omentum and are considered as factors responsible for recruiting of cancer cells to the omentum. Adipocytes stimulate growth of OC cells, probably due to their role in the TAGs accumulation. In vitro, in the presence of the OC cells, adipocytes secrete more FFAs and glycerol than adipocytes cultured alone, which suggests that cancer cells could induce adipocyte lipolysis [588].

In an intraperitoneal mouse model of ovarian cancer, metformin reduces OC dissemination by blocking MCP-1 release from adipocytes. An important factor regulating tumor growth and metastasis in OC is integral membrane fatty acid receptor (CD36). Reduced CD36 expression decreases FFA uptake, intracellular cholesterol levels, and lipid droplet accumulation, leading to lower ROS generation in cancer cells. Omental adipocytes induce CD36 expression in ovarian cancer cells, promoting FFA uptake. Conversely, CD36 inhibition reduces migration, adhesion, and invasion in vitro, decreasing metastatic burden in xenograft models of ovarian cancer metastasis. CD36 expression is more frequent in visceral metastases than in normal ovarian tissue or primary ovarian cancer lesions. It is also associated with poorer prognosis and shorter survival in several cancers, including squamous cell lung carcinoma, bladder cancer, and luminal-type breast cancer [611].

Adipocytes can impair the effectiveness of chemotherapy both directly and indirectly. They release various soluble factors, including hormones, lipids, cytokines, and miRNAs, which can alter chemotherapy sensitivity in ovarian cancer cell lines, particularly reducing paclitaxel efficacy. Additionally, adipocytes influence FABP4 expression, and its inhibition has been linked to increased resistance to carboplatin.

Beyond chemotherapy resistance, adipocytes promote metastasis by releasing hepatocyte growth factor, which induces E-cadherin loss in ovarian cancer cells, facilitating EMT. Furthermore, adipokines enhance ovarian cancer cell migration, secondary lesion formation, and metabolic reprogramming [612,613].

#### 3.11.3. CSCs in Ovarian Cancer

An analysis of ovarian tissue has revealed the presence of a small subpopulation of Nanog-positive cells within the ovarian surface epithelium (OSE). These cells are likely cancer stem cells, which are dormant in ovarian surface and tubal epithelium. It is also noteworthy that the majority of OC cells originate from OSE, confirming the prominent role of CSCs in the progression and recurrence of OC. The panel of ovarian CSC markers that have been reported include CD24, CD44, CD117, CD133, ALDH1, ABC transporter proteins, EpCAM, Nestin, Oct4, Nanog, Sox2, SSEA4 and SCF. Of these, CD24, CD44 and CD133 are among the most prominent markers. CD24+ cells in OC have been observed to exhibit anoikic resistance, enhanced tumor growth, colony formation, an EMT phenotype and stem-like properties, including self-renewal. CD133 expression is associated with a poor prognosis and is also implicated in increased platinum resistance and metastasis. Furthermore, the expression of Nanog has been demonstrated to modulate platinum resistance and EMT in OC. The heterogeneity of the tumor microenvironment is a defining feature of OC. The distinctive mutational profile of epithelial OC contributes to its rarity and complexity, rendering it challenging to diagnose in comparison to other cancers. A number of regulatory pathways, including Wnt, STAT3, Hedgehog, BMI1, Notch and NF-κB, are aberrantly activated, thereby facilitating the self-renewal of CSCs in OC. The dysregulated cell cycle and hyper-proliferation of CSCs are governed by a number of key modulators, including β-catenin, mitogen-activated protein kinase (MAPK), NF-kB, paired box 6 (PAX6), forehead box O3 (FOXO3) and signal transducers and activators of transcription 2 (STAT2). In the context of combating OC, drugs are being designed with the objective of targeting self-renewal signaling pathways that have the potential to impede the survival, differentiation and replication of stem cells. A number of such drugs are currently in clinical use, including percept (OMP-54F28) and DAPT (GSI-IX). The former targets the Wnt ligand (Wnt pathway), while the latter acts on the γ-secretase (Notch pathway) [614,615,616,617,618,619].

#### 3.11.4. T Cells in Ovarian Cancer

In ovarian cancer, TILs including B and T cells are generally localized in primary lesions or in omental metastases and their presence correlates to a better prognosis, especially when they are enriched with T cells. The CD3+ TILs are found in >50% of advanced stages of epithelial OC. The presence of CD8+ T cells and a high CD8+/Treg ratio are positive prognostic factors, correlating with longer survival. CD4+ T cells support CD8+ T cell activation, enhancing cancer cell elimination, and their high numbers are also associated with improved clinical outcomes. In contrast, the lack of TILs corresponds to the poor prognosis [620].

CD4+ T cells influence ovarian cancer progression, particularly through the Th2 subpopulation, which secretes IL-4 and correlates with poor prognosis. CD4+CD25+Foxp3+ Tregs are induced by COX-2/PGE-2 secretion from ovarian cancer cells, promoting tumor growth. Their accumulation is associated with a higher risk of death, while their depletion enhances the production of IL-6, IL-12, IFN-γ, and TNF-α [621,622].

In early-stage OC, Th17 cells promote Th1 recruitment and increase Treg numbers, with CD4+ Tregs capable of converting into Th17 cells upon IL-2 stimulation. Treg levels correlate with EpCAM+ expression in cancer-derived epithelial cells in ascites and are associated with cancer progression. Conversely, CD8+ T cell infiltration into tumors is linked to longer survival in OC patients. Upregulation of the number and activity of CD8+ T cells in OC allows for eradication of ovarian cancer cells through granzymes, IFN-γ and TNF-α secretion after cancer-antigen recognition. Unfortunately, most CD8+ T cells in OC are dysfunctional and only 10% of them are capable of properly recognizing cancer antigen and exhibiting their cytotoxic activity. OC cells can impact on the anti-cancer function of CD8+ T cells through signaling pathways including CTLA-4, PD-1, LAG-3 or TIM-3 [620,621,623,624].

#### 3.11.5. NK Cells in Ovarian Cancer

Several molecules may affect NK cells cytotoxicity in ovarian cancer. For instance, in the ascites of ovarian cancer patients, mature NK cells overexpress PD-1 that corresponds to their poor proliferation rate and diminished cytotoxic activity. In turn, ovarian cancer cells express a ligand of NK cells receptor UL16-binding protein 2 (ULBP2), which is an indicator of poor prognosis, due to disruption of T cell function [625].

In xenograft models, NK cells exhibit a CD56^bright^CD16+ phenotype, typical of ovarian cancer patients. These NK cells expand rapidly and maintain cytotoxic activity against cancer cells. However, increased CD56^bright^ activity does not correlate with cytokine secretion [626,627].

In turn, the presence of the CD57+ NK cells and CD103+ NK cells within the tumor mass in OC is associated with an improved survival; however, their function may be inhibited by increased secretion of migration inhibitory factor (MIF), contributing to downregulation of the expression of the NKG2D activating receptors on NK cells. Moreover, the expression of another activating receptor on NK cells, NKp30, is diminished on cells in the ascites of ovarian cancers [628].

#### 3.11.6. B Cells in Ovarian Cancer

In ovarian cancer, various B cell populations, including naïve, memory, plasma, and regulatory B cells (Bregs), are detected. While B and plasma cells generally have a neutral or positive prognostic effect, some studies suggest a negative impact. For instance, CD19+ B cell infiltration into the omentum correlates with poor survival in high-grade serous carcinoma (HGSC). Similarly, a high percentage of CD19+ B cells along with NK cells in metastatic ovarian carcinoma is linked to poor prognosis. In contrast, CD20+ B cells are associated with longer survival, particularly when colocalized with activated CD8+ T cells [629,630].

In epithelial ovarian cancer, plasma cell infiltration influences prognosis and overall survival. High CD138 expression on plasma cells correlates with reduced OS [631]. B cell depletion in murine OC models inhibits CD8+ T cell responses by reducing CD107 (a degranulation marker) expression. B cells also contribute to the immune response in metastatic HGSC. Bregs (B10) play a key role in immune suppression through cell-to-cell interactions and facilitate IL-10+ IgM+ memory B cell generation [632,633].

Bregs promote tumor growth and suppress immune responses, correlating with higher Treg numbers in OC. Additionally, ovarian cancer cells enhance B cell transformation into Bregs, further inhibiting immune responses [634].

#### 3.11.7. Dendritic Cells in Ovarian Cancer

In high-grade serous OC, mature DCs participate in CD20+ B cells infiltration impacting on the OS after chemotherapy. This suggests that the number of DCs can be considered as a prognostic factor. Ovarian cancer cells may limit the activity of the DCs by disturbing the process of their activation, differentiation, recruitment and the process of antigen presentation to immune cells. In ovarian cancers, myeloid DCs are present in the draining lymph nodes, and may influence T cells activation. In general, DCs fail to maintain T cell proliferation, due to the secretion of TGF-β, which induces CD4+ T cells differentiation into the Tregs [635].

Typical for OC are so-called vascular DCs—DCs involved in tumor vasculogenesis. Vasculogenesis of tumors requires, on the one hand, the accumulation of vascular DCs and, on the other hand, the absence of anti-angiogenic mDCs. In addition, in ovarian cancer the infDCs can be found. They are the most abundant subset of DCs, facilitating the cancer progression through IL-6 and galectin-1 releasing. The correlation between the number of infDCs and clinical outcomes in patients with OC is unclear, probably due to the heterogeneity of the TME. On the other hand, the accumulation of pDCs in tumor mass, especially at advanced stages of OC, predicts early relapse, whereas the ratio of mDCs and pDCs within ascites does not correlate with the survival time of patients [636,637,638].

#### 3.11.8. MDSCs in Ovarian Cancer

The number of MDSCs present in the tumor mass, ascites and peripheral blood of OC patients is highly elevated. This abundance strictly correlates with the elevated levels of IL-6 and IL-10, advanced cancer stages and poor survival of patients. Additionally, MDSCs suppress the T cell activity in several different ways, for instance by depletion of arginine which contributes to T cell and NK cell proliferation. In human ovarian cancers, MDSCs infiltrations correspond to the shorter OS and increased levels of CXCR2 ligands involved in the EMT process. MDSCs play a role in T cells immune response modulation and may be considered as biomarkers in this cancer. The number of expressing the VEGF receptor on their surface (MDSCs) in the TME is increased in ovarian cancer with high VEGF expression, leading to the suppression of the local immune response. In the murine model of epithelial OC, MDSCs are also considered a driving factor for immunosuppression [638,639,640].

#### 3.11.9. Neutrophils in Ovarian Cancer

A 2017 meta-analysis found that a reduced neutrophil-to-lymphocyte ratio was associated with improved OS and progression-free survival (PFS) in ovarian cancer patients. Neutrophil influx into the premetastatic omentum is essential for metastasis in OC models. Inflammatory factors from ovarian tumors trigger neutrophils to release chromatin networks, called neutrophil extracellular traps (NETs), which are present in premetastatic ovarian tumors in mice and early-stage ovarian cancer in women. NETs bind OC cells, and their formation depends on neutrophil-specific peptidyl arginine deiminase 4 (PAD4). Pharmacological inhibition of PAD4 reduces NET formation and premetastatic omental colonization, suggesting that NETs contribute to establishing a premetastatic niche favorable for ovarian cancer cell implantation. Targeting NET formation may offer a strategy to prevent omental metastasis [641].

#### 3.11.10. Macrophages in Ovarian Cancer

A significant difference has been observed in both progression-free and overall survival in patients with stage III–IV ovarian cancer expressing high and low levels of CD163 (M2 TAM marker). The low CD163 patients exhibited significantly higher survival rates. Additionally, the CD163/CD68 ratio (equivalent to the M2:M1 ratio) was a negative predictor of overall survival. A meta-analysis of nine studies, comprising a total of 794 OC patients, demonstrated that a high M1/M2 ratio in tumors was associated with a more favorable prognosis. Exosomes play a pivotal role in the cross-talk between OC cells and the immune system. Cancer cell-derived exosomes containing miR-200b induce M2 macrophage polarization via KLF6 (Kruppel-like factor 6) reduction. Exosomes with microRNA-222-3p polarize macrophages through the SOCS3/STAT3 pathway. TAM-derived exosomes increase the Treg/Th17 ratio by inhibiting STAT3, creating an immunosuppressive environment. Hypoxia enhances microRNA940 release from OC epithelial cells, driving M2 TAM polarization. Secreted proteins like IL-4 activate the PI3K pathway, inducing M2 polarization. GATA-binding protein 3 (GATA3), secreted primarily by TAMs in high-grade serous ovarian cancer, promotes tumor progression, angiogenesis, and chemotherapy resistance. TAMs also release EGF, activating the EGFR-ERK pathway and increasing VEGF expression, leading to angiogenesis [180,642,643,644,645,646,647,648,649,650].

In epithelial ovarian cancer, TAMs affect TECs function through the production of IL-8. IL-8 signaling positively influences the survival and proliferation of TECs, as well as stimulates the promotion of angiogenesis by them [651,652].

### 3.12. Cervical and Endometrial Cancers

Cervical cancer belongs to the group of tumors associated with HPV infections. Predominantly, cervical cancer is caused by HPV-16 or HPV-18. In addition to HPV infections, risk factors include age of first sexual intercourse, multiple sexual partners, prolonged use of oral contraception, cervical dysplasia, early menarche, late menopause, age, obesity, smoking, diabetes, menstrual disorders, anovulatory cycles, polycystic ovary syndrome and hormone replacement therapy. HPV-16 and HPV-18 encode genes for the E6 and E7 oncoproteins, which inhibit the functions of p53 and Rb, contributing to HPV’s ability to evade the immune response. Both oncoproteins interfere with the NF-κB signaling pathway, thereby increasing the risk of cervical cancer development [653,654,655].

Endometrial cancer can be classified as type I or type II. Type I accounts for 80% of endometrial cancer diagnoses and is associated with a favorable prognosis. This type of cancer is characterized by an excessive response to both endogenous and exogenous estrogens, whereas type II is characterized by poor prognosis and a more aggressive course [654].

#### 3.12.1. CAFs in Endometrial Cancer

In endometrial cancer, CAFs primarily originate from normal fibroblasts. CAFs in endometrial cancers secrete cell cycle-related proteins such as MAD2L1, CDKN1A, and CEBPβ, as well as growth factors IGF and TGF-β, which influence apoptosis evasion, migration, and invasion. Endometrial CAFs secrete IL-6, which induces cancer cell proliferation through STAT-3 transcriptional activity dependent on c-Myc expression. It has been shown that the downregulation of miR-31 in CAFs is associated with increased expression of the homeobox gene *SATB2*, which affects cancer cell migration and invasiveness in endometrial cancers. Exosomal nuclear paraspeckle assembly transcript 1 (NEAT1) from CAFs contributes to the progression of endometrial cancer through the STAT3/YKL-40 pathway, dependent on miR-26a/b-5p. miRNA-148a from CAFs regulates the migration of endometrial cancer cells by activating the Wnt/β-catenin pathway. In endometrial cancers, activation of the PI3K/AKT pathway affects EMT and cancer cell stemness [656,657,658,659,660,661].

#### 3.12.2. T Cells in Cervical and Endometrial Cancers

In dysplastic cervical cancer, the number of TILs is high and correlates with the grade of dysplasia. Inhibition of CD4+ and CD8+ T cell response to HPV accelerates cervical cancer development, as HPV is a major risk factor in this cancer group. In early-stage cervical carcinoma, a high percentage of CD8+ T cells localized in the epithelium correlates with better prognosis. Moreover, the number of CD3+ T cells and CD8+ T cells within tumor mass and in peritumoral tissues correlates with the risk of relapse. The higher the number of CD8+ T is detected, the better the response to treatment in cervical cancer patients. At the same time, the number of Tregs in cervical cancer is increased, which is associated with an increased number of HPV copies and correlates with an increased recurrence rate and shortened time of OS. In general, cervical cancer expresses high amounts of CCL20 which enhances infiltration of Th17 cells into the tumor mass. Furthermore, cytokines released by Th17 cells generate chronic inflammation in the TME and facilitate HPV infection in patients with cervical cancer [662,663].

In turn, in endometrial cancer, the number of CD8+ T cells is increased which corresponds to a long OS. Moreover, the patients have high percentages of Tregs and memory T cells (CD45RO+), but immune cell infiltration densities differ between individuals. The number of CD8+ T cells is considered as the strongest indicator of recurrence, especially in patients with the p53 mutations [663].

#### 3.12.3. NK Cells in Cervical Cancer

Cervical cancer cells employ several strategies to evade NK cell responses. NK cells play a crucial role as the first line of defense during HPV infections. The E7-HPV oncoprotein, however, inhibits the expression of CXCL14, a chemokine involved in recruiting NK and T cells to infected lesions. Additionally, HPV infection negatively affects the expression of MHC class I polypeptide-related sequence A (MIC-A), a ligand recognized by NK cells, which limits the elimination of HPV-infected cells [664].

Keratinocytes play a significant role in activating the immune response in patients with cervical cancer by presenting HPV antigens to target cells. In HPV-infected cells, however, the expression of HLA is reduced, leading to NK cell inactivation [665].

In the uterine cervix, NK cells predominantly exhibit a CD56^bright^CD16- phenotype. This phenotype can be modulated by HPV infection. Interestingly, a higher percentage of NK cells is found in low-grade cervical cancers, which correlates with a lower number of infected cells. However, these NK cells fail to release IL-2 and exhibit limited cytotoxicity [666,667].

In cervical cancer patients, tumor cells evade NK cell-mediated elimination by inhibiting the production of cytokines essential for NK cell activation. Moreover, tumor cells release immunosuppressive factors such as TGF-β and IL-10, which further contribute to NK cell dysfunction. A reduction in the number or function of NK cells is associated with worse prognosis and shorter survival [665].

#### 3.12.4. B Cells in Cervical Cancer

The number of B cells positively correlates with the number of Bregs. B10 cells play a key role in cervical cancer progression and reveal longer lifetime, and lower HLA class II expression. Further, they also express PDL-1 on their surface that is associated with the enhancing of the cytotoxic activity of CTLs. The blockade of the PD-1 receptor leading to HPV-specific memory B cells development that improves patients clinical outcomes. Due to the fact, that Bregs respond to the TNF-α through inducing cancer tolerance, their enhanced activity is associated with the aggressive course of the cancer, and higher possibility of metastasis [668,669,670].

#### 3.12.5. Dendritic Cells in Cervical Cancer

In cervical cancer, in addition to classical dendritic cells (cDCs), Langerhans cells (LCs) and epithelial cells present antigens to T cells. HPV infection reduces DC percentages in cancer lesions and inhibits CCL20 expression, which is crucial for DC recruitment to the tumor. Despite this reduction, DCs remain a significant cell population. Furthermore, the decline in DC numbers during HPV infection may contribute to cervical cancer progression [669,670].

In cervical cancer patients, DCs often exhibit an immature phenotype but can mature in response to cancer antigens. However, cervical cancer cells secrete high levels of IL-6, which limits CCR7 expression on DCs, preventing their migration to lymph nodes. Additionally, IL-23-secreting DCs promote Th17 cell infiltration into the tumor mass, while IL-10 from Th2 cells contributes to DC reprogramming within the tumor microenvironment [671,672].

#### 3.12.6. MDSCs in Cervical and Endometrial Cancer

Cooperation between cervical cancer cells and peripheral blood mononuclear cells frequently leads to transformation of monocytes into MDSCs. In a mouse model of endometrial cancer, the main factors involved in this process are G-CSF, IL-6 and estradiol. MDSCs in endometrial cancer inhibit cytotoxicity of CD8+ T cells, regulate angiogenesis, form a premetastatic niche and modify stem-like features of cancers cells, which influences endometrial cancer progression, metastasis and even resistance to chemo- and/or radiotherapy [673,674].

In contrast, the number of circulating MDSCs negatively correlates with the number of TILs and their increasing percentage is considered as an unfavorable risk factor in advanced clinical stages, lymph node infiltration and a poor response to first-line treatments [675].

In mouse models of cervical and endometrial cancers, levels of G-CSF, IL-6 and estradiol contributing to MDSC generation are significantly increased. Similarly, in both types of cancers, numbers of mo-MDSCs and PMN-MDSCs are increased in peripheral blood, lymph nodes and in tumor mass. The high number of MDSCs correlates also with leukocytosis and high concentrations of PGE2 (in serum) and TGF-β (in tumor mass) [675].

In pregnant patients suffering from cervical cancer, mobilization of MDSCs from bone marrow by estradiol enhances their immunosuppressive activity, leading to cancer progression [674].

### 3.13. Prostate Cancer

Prostate cancer (PC) usually affects middle-aged men, and its risk factors include genetic predisposition, ethnicity, age, obesity, and various environmental factors. PC primarily affects men between the ages of 45 and 60 and is a leading cause of cancer-related deaths in men. The main cause of prostate cancer is believed to be gene mutations involved in the androgen pathway and testosterone metabolism. Prostate cancer can be classified as either androgen sensitive or androgen insensitive [676,677,678].

During aging, there is an upregulation of pro-inflammatory cytokines and growth factors, as well as molecular and structural changes such as alterations in ECM composition and increased recruitment of inflammatory cells to the matrix. This can contribute to prostate enlargement, prostatitis, and PC. The normal TME can undergo further phenotypic changes. There is a loss of well-differentiated smooth muscle cells and an expansion of CAFs. As a result, a reactive stroma is formed, characterized by a large presence of CAFs, altered ECM composition, neovascularization, infiltration of immune cells [679,680].

Enhanced cytokine secretion, which is followed by angiogenesis, epithelial–mesenchymal transition (EMT), and metastasis. All of these factors contribute to the development of advanced and metastatic PC [681].

Obesity stimulates adipose tissue to release inflammatory cytokines (IL-6, MCP-1, TNF-α), the levels of which are associated with prostate cancer progression in clinical studies. In addition, adipose tissue can secrete androgens and androgen precursors (testosterone, dihydrotestosterone, androstenedione, progesterone, and dehydroepiandrosterone) [682].

#### 3.13.1. CAFs in Prostate Cancer

CAFs communicate with prostate cancer cells and influence their metabolism as well as their drug sensitivity. CAFs can support the growth of cancer cells, affect their viability, and promote metastasis formation. Studies have shown that CAFs derived from prostate tumors increase the growth of PC cell lines BPH-1 and LNCaP, in contrast to normal fibroblasts derived from the prostate gland [679,683,684].

The sources of CAFs in PC are normal fibroblasts, MSCs, adipocytes, pericytes, and endothelial cells. Three subtypes of CAFs have been identified in prostate cancer: myCAFs, iCAFs, and apCAFs. myCAFs, due to their contractile properties and high expression of α-SMA, have a significant impact on ECM remodeling and the modification of the reactive stroma. They also control the motility of PC cells and support their survival. The role of apCAFs has not yet been fully elucidated. They are likely associated with resistance to anti-cancer therapies. In PC, iCAFs produce chemokines and cytokines to recruit regulatory T lymphocytes and alter macrophage polarization, as well as inhibit the cytotoxic activity of NK cells through the production of MMPs. iCAFs form a complex network of interactions with macrophages and M2 polarization, as well as with cancer cells, creating a pro-inflammatory immunosuppressive environment. In this way, they influence cancer cell survival, invasiveness, and metastasis formation [27,32,168,528,685,686,687].

CAFs can promote tumor invasion by stimulating cancer cell motility through modulation of ephrin signaling. Direct contact between cancer cells and CAFs likely stimulates tumor growth via Notch signaling in stromal cells. CAFs may also act indirectly by overproducing ECM components such as fibronectin, collagen, hyaluronic acid, and tenascin-C, increasing cancer cell proliferation and invasion, leading to metastasis. Prostate CAFs induce ECM remodeling by secreting MMP1, -2, -7, -9, and -14, as well as creating an imbalance between MMPs and tissue inhibitors of metalloproteinases (TIMPs), which also contributes to the increased invasiveness of PC [688,689,690,691,692,693].

Androgens can significantly affect CAFs. They stimulate DNA synthesis in CAFs and change their phenotype to a migratory one. It has been shown that at low androgen concentrations, the androgen receptor binds to Src and p85α, leading to increased MAPK and Akt activity, D1 regulation, and increased cell proliferation. Conversely, at higher androgen concentrations, the stromal androgen receptor associates with filamin A (FlnA) and integrin-β1, activates Rac, and promotes cell motility. Additionally, the AR/FlnA complex regulates invasiveness in CAFs expressing androgen receptors [694,695].

CAFs produce VEGF, which enhances abnormal tumor angiogenesis, and they also induce a more stem-like phenotype in PC cells. IL-6, produced by either PC cells or CAFs, is a crucial factor in the PC microenvironment, affecting various aspects, including androgen insensitivity. Consequently, clinical trials are underway investigating the use of IL-6 inhibitors or related STAT3 inhibitors in the treatment of PC [696].

#### 3.13.2. Adipocytes in Prostate Cancer

After contact with the PC cells, adipocytes are reshaped to a less differentiated and form prostate cancer cells-associated adipocytes (CAAs). It has been shown that preadipocytes co-cultured with PC-3 cell line release higher amount of adipokines, TNF-α, IL-6, osteopontin and MMP9. Moreover, they are transformed into neoplastic-like cells. PCs are affected by adipose-dependent factors increasing their proliferation and migration. The bone marrow adipocytes are derived from a population of multipotent progenitor bone marrow stroma cells (BMSCs) that also gives a rise to the bone cells They secrete proinflammatory cytokines and adipokines with local and systemic effects (TNF-α, adiponectin) [74].

Bones are the most popular metastatic site in case of PC, and BMSCs are considered as key factors of the progression and exacerbation of these bone metastases—there is a positive correlation between increased number of BMSCs and the progression of PC cells in the bone marrow niche [697].

#### 3.13.3. CSCs in Prostate Cancer

Knowledge about the role of CSC in PC is constantly evolving. Available data on putative markers, the cell of origin and the location of prostate cancer stem cells (PCSCs) within the organ are inconsistent. The majority of studies investigating PCSC have employed established cell lines, primary tumors or xenografts in immuno-deficient mice. A number of markers have been proposed for the characterization of PC and PCSCs, including cell surface markers, markers of self-renewal, pluripotency and resistance to therapy. Using a combination of surface markers, CD44+, α2β1 integrin high, and CD133+, Collins et al. were able to isolate rare cells from human primary PC. These cells demonstrated the capacity for self-renewal in vitro. The same combination was used to isolate prostate CSCs from the DU145 cell line. It is noteworthy that CD44, a glycoprotein implicated in cell–cell interactions, cell adhesion and migration, has been identified as a marker of stemness for CSCs in a multitude of organs. Patrawala et al. demonstrated that CD44+ PC cells derived from xenografted human tumors exhibited a greater propensity for tumor formation and metastasis compared to CD44 cells. Hurt demonstrated that CD44+CD24- prostate stem-like cells isolated from the LNCaP cell line exhibited tumor-forming potential following the injection of as few as 100 cells into NOD/SCID mice [605,698,699,700,701,702,703].

#### 3.13.4. T Cells in Prostate Cancer

In PC, T cells accumulation affects mainly cancers induced by diet. The number of CD4+ T cells correlates with poor survival in PC patients, as these cells release high levels of chemokines, such as CXCL9. Infiltrating T cells promote PC cell proliferation through the FGF11/miRNA 541/AR/MMP9 signaling pathway. Additionally, the CCL5/CCR5 axis drives PC progression. TILs in PC exhibit high heterogeneity and, as in other cancers, play a critical role in cancer progression. Elevated Treg levels in PC patients are associated with poor survival and increased mortality, with their numbers rising significantly in the bone metastatic TME of PC. Conversely, Treg depletion enhances the anti-cancer immune response in mouse models [704,705,706,707,708].

#### 3.13.5. NK Cells in Prostate Cancer

NK cells interact with PC cells and limit prostate growth through direct contact. In patients suffering from PC, the number of the CD56^bright^ NK cells and the number of NK cells producing IFN-γ is significantly lower than in healthy individuals, and correlates negatively with the cancer progression in patients with the androgen-deprived PC [709].

#### 3.13.6. B Cells in Prostate Cancer

In PC, the role of CD20+ B cells remains unclear, but evidence suggests they contribute to castration-resistant prostate cancer (CRPC). Clinical observations and murine models of androgen ablation indicate that B cells may promote cancer recurrence. In a murine model of PC, several splice variants of Igs (immunoglogulins) and complements (C4b) were detected. B cell-mediated and Igs-mediated responses contribute to the high-fat diet-related chronic inflammation and PC growth. Additionally, tumor-infiltrating B cells produce large amounts of lymphotoxin, a cytokine that belongs to the TNF family that contributes to activation of the IkB kinase α and STAT3-dependent pathway, which in turn increases the survival of androgen-deprived PC cells. The net result of this is a castration-resistant state which develops in murine models of PC. Furthermore, Igs released by B cells play a crucial role in the promotion of cancer progression. In human PC cell line LNCaP, silencing of IgG induces apoptosis and suppresses proliferation and migration of these cells. At the same time, complement activation also contributes to PC progression and metastasis [710,711,712,713,714].

#### 3.13.7. Monocytes and Related Cells in Prostate Cancer

During the course of PC, monocytes display dysfunction especially in advanced stages [710]. They exert their effects through direct cell-to-cell contact and cytokine release. A high number of MDSCs correlates with a poor prognosis in prostate cancer patients [715].

In a mouse model of prostate cancer (TRAMP mouse), IL23-secreting MDSCs activate the androgen receptor pathway promoting cancer cell survival and proliferation in the absence of androgens. Blockade of the IL-23 secretion influences the MDSCs-related resistance to the castration process. Additionally, CXCL5 released from the PC cells attracts MDSCs secreting CXCR2. Elimination of MDSCs as well as inhibition of the CXCL5-CXCR2 signaling positively influences anti-cancer immune response in these models [716,717].

In PC patients, counts of mo-MDSCs with CD14+HLA-DR-/low phenotype and Tregs are increased. The elevated count of mo-MDSCs is considered a negative prognostic marker in these patients [718,719].

#### 3.13.8. Neutrophils in Prostate Cancer

A study conducted in 2020 revealed that neutrophils directly inhibited STAT5 expression, which appeared to be a prerequisite for neutrophil-dependent PC apoptosis. Recent studies have demonstrated that STAT5 plays a role in the growth and invasion of metastatic prostate cancer to bone. Inhibition of STAT5 via siRNA has been shown to significantly increase caspase 7 expression and prostate cancer apoptosis. Thus, STAT5 represents a potential target for the prevention of aggressive, metastatic PC progression [720,721,722].

#### 3.13.9. Macrophages in Prostate Cancer

Similarly to other forms of cancer, chronic inflammation in prostate cancer is believed to act as a precursor to tumor formation. In prostatic inflammatory atrophy, a precancerous condition, macrophages are observed to coalesce at sites where inflammation-driven neoplasia results in disruption of the epithelial lining of the prostate. In primary PC, both pro- and anti-inflammatory TAMs represent a substantial proportion of the immune cells infiltrating the TME. Researchers are beginning to clarify the roles of different cell populations in PC progression. Most studies indicate that macrophages contribute significantly to primary PC progression, with meta-analyses linking macrophage infiltration to more aggressive disease and poorer prognosis. Anti-inflammatory macrophages promote PC through immune-suppressive and angiogenic effects, key hallmarks of progression. PC cells secrete factors like colony-stimulating factor 1 (CSF-1) and CCL2, which recruit monocytes and macrophages to facilitate these processes [723,724,725,726].

### 3.14. Kidney Cancer

Kidney cancer constitutes the sixth most common cancer in men, and tenth in women. Renal cell carcinoma (RCC) is the most common kidney cancer. There are three major histological subtypes of RCC: clear cell renal cell carcinoma (ccRCC), papillary RCC and chromophobe RCC. Among these subtypes ccRCC is the most prevalent. Generally, RCC is a group of heterogeneous cancers with different clinical symptoms. The most common risk factors contributing to the development of RCC include age and gender; however, ethnicity, smoking, hypertension, and obesity are also significant. In addition, several diseases such as chronic kidney disease (CKD), acquired renal cystic disease (ARCD), end-stage renal disease (ESRD), and factors like red meat consumption, viral hepatitis, vitamin D levels, triglycerides (TAGs), and type II diabetes can also play a role. RCC can originate from different cells in the nephrons and is classified as clear cell, papillary, and chromophobe carcinomas. Clear cell carcinoma has a poor prognosis, while papillary carcinoma has a more favorable outcome. In contrast, chromophobe RCC is the least aggressive and has the best prognosis among RCC subtypes [727,728,729].

The TME in ccRCC consists of both immunostimulatory and immunosuppressive factors. Predominantly, the immune infiltrates in ccRCC are composed of CD8+ T cells, which improve clinical outcomes. In the TME of RCC, T cells constitute 51% of immune cells, myeloid cells account for 31%, natural killer (NK) cells represent 9%, and B cells make up 4% [730].

The pathogenesis of ccRCC is associated with an early genetic loss of von Hippel–Lindau (VHL), accumulation of HIF1-α and HIF2-α and elevated expression of VEGFA. RCC is a highly vascularized tumor generally insensitive to cytotoxic chemotherapy [727,731].

#### 3.14.1. Non-Immune Cells in Renal Cell Carcinoma

The differentiation of normal fibroblasts into CAFs is induced by TGF-β and PDGF in renal cancer. CAFs produce pro-tumoral cytokines such as IL-6, IL-8, IL-10, TNFA, and TGF-β, and also generate ECM components that hinder the function of T cells in the TME [732,733].

In ccRCC, anti-VEGF receptor tyrosine kinase inhibitors (VEGFR-TKIs) promote the development of CAFs, which in turn enhance tumor aggressiveness, metastatic potential, and resistance to therapies. It has been demonstrated that anti-angiogenic therapies (such as VEGFR-TKI and sunitinib), which modify the vessel structure and cellular environment by increasing CAFs activity, lead to limited efficacy of the treatment. CAFs induced by VEGFR-TKI are associated with lymphatic vessel development and lymph node spread [732].

Adipocytes may regulate the activity of cancerous and non-cancerous renal epithelial cells by releasing free FFAs, hormones and adipokines which influence cancer progression by promoting low-grade chronic inflammation. The risk of ccRCC development can be associated with polymorphism in leptin and adiponectin gene receptors. High expression of these receptors in human adipose explants from renal carcinomas, which corresponds to enhanced lipolysis, is associated with rapid cancer progression [734,735].

Considerable research has focused on identifying RCC CSCs, with several markers being evaluated. Prominin-1 (CD133), a glycoprotein on stem and progenitor cells in normal tissues, is a proposed CSC marker in various cancers. In vivo CD133+ cells alone lack renal tumor-forming capacity. However, co-transplantation of RCC cells with CD133+ enhances tumor engraftment, vascularization, and growth. CD24, widely expressed but most prevalent in progenitor and stem cells, has been linked to RCC progression, overall survival, and relapse-free survival [736,737].

#### 3.14.2. Immune Cells in Renal Cell Carcinoma

In the TME of ccRCC patients, TLSs structures are composed mainly of CD20+ B cells surrounded by T cells resembling lymph follicles of secondary lymphoid organs. T cells present in TLSs belong to CD4+ T cells, CD8+ T cells and Tregs. The TME in metastatic renal cancers is composed of high numbers of CD8+. However, most express the PD1+ marker and exhibit reduced cytotoxic potential and low polyclonality, leading to worse prognosis and clinical outcomes. A high effector T cell-to-Treg ratio is associated with a lower recurrence rate in patients with ccRCC. In contrast, in advanced and metastatic ccRCC, T cells show significantly lower T cells receptors diversity compared to normal kidney tissue [738,739,740].

In ccRCC patients, NK cells exert both cancer-promoting and cancer-inhibiting effects. On the one hand, they are involved in cytokines and growth factors secretion, supporting renal cancers growth and angiogenesis. On the other hand, NK cells can eliminate renal cancer cells using perforins or granzymes. It is therefore important to maintain the appropriate balance between pro- and anti-cancer effects of NK cells, although the ultimate effect of their activity depends on NK cell subtypes and TME composition [738,740].

In renal cell carcinoma, tumor-infiltrating B cells are localized in tertiary lymphoid structures, which exist at various stages of cancer maturity. B cells are recruited to the TME by cytokines and chemokines released by CD8+ and CD4+ T cells, though their function is negatively impacted by Tregs and MDSCs [741].

Interestingly, TILBs likely exert a pro-tumorigenic effect in clear cell renal carcinoma, contrasting with their positive role in breast, colon, lung, and ovarian cancers. This pro-tumor effect is linked to increased TAM2 levels and T cell exhaustion, promoting cancer progression. High tumor-infiltrating B cell levels in these patients correlate with poor prognosis and reduced survival. However, some studies indicate that B cells in TILBs secrete large amounts of IgG and IgA, contributing to cancer inhibition [742].

TANs in RCC play a multifaceted role in tumor progression. TANs promote tumor growth, angiogenesis and metastasis by the secretion of pro-angiogenic factors, including VEGF, and by degradation of the extracellular matrix, which facilitates tumor cell migration. Additionally, neutrophils can facilitate the development of an inflammatory environment within the tumor, thereby supporting tumor growth and the formation of new blood vessels. This increases the tumor’s supply of oxygen and nutrients. Additionally, TANs influence the immune response, frequently impeding T cell activity and fostering immunosuppression, which facilitates tumor development. A high neutrophil-to-lymphocyte ratio is an unfavorable prognostic marker in RCC, indicating an active inflammatory response linked to poor prognosis. Additionally, studies show that TANs promote therapy resistance, including immunotherapies, by supporting tumor survival and creating a growth-favorable microenvironment [743].

Studies link M2 macrophage infiltration to poor prognosis in cancers, including RCC. M2 macrophages promote RCC migration, invasion, and EMT via CXCL13 secretion. Higher M2 macrophage levels correlate with increased RCC recurrence, highlighting their role in tumor progression and therapy resistance. A 2011 study identified most CD68+ TAMs in RCC as M2 macrophages, expressing CD204 and CD163. It also showed that direct macrophage–RCC coculture induces M2 polarization, likely due to RCC membrane-type M-CSF expression. A recent study found that RCC-derived TAMs produce high levels of CCL2, recruiting monocytes, and IL-10, an immunosuppressive cytokine. Notably, TAMs from larger tumors exhibited increased IL-10 production, suggesting that either larger tumors enhance M2 polarization or M2 macrophages accelerate tumor growth [744,745,746,747].

### 3.15. Bladder Cancer

Bladder cancer (BC) is the most common malignancy of the urinary tracts. The incidence of BC varies significantly depending on the geographical region. It is suggested that disparities in smoking, obesity and alcohol consumption contribute to these differences. The most significant risk factor appears to be smoking. A strong correlation has been demonstrated between tobacco use and the incidence and mortality of BC [748,749,750,751].

Bladder walls are composed of five to seven layers of epithelial cells, with the urothelium underlying fibroconnective tissue, blood vessels, thick muscular bundles, and a fat layer. Most bladder cancers arise from urothelial cells (95%) and other forms of BC are rare. Urothelial cancers are classified as muscle-invasive bladder cancer (MIBC) or non-muscle-invasive bladder cancer (NMIBC) [752].

The bladder cancer microenvironment is composed of immune cells, MSCs, endothelial cells, ECM components and a wide spectrum of inflammatory mediators. The association between bladder cancer cells and the TME impacts treatment effectiveness [753].

#### 3.15.1. Non-Immune Cells in Bladder Cancer

In BC, CAFs originate from fibroblasts, endothelial cells, MSCs, and possibly also from smooth muscle cells. Two subtypes of CAFs are distinguished in BC: iCAFs and myCAFs. iCAFs secrete various cytokines such as CXCL1, CXCL2, CXCL12, IL-6, and CXCL14. myCAFs regulate ECM remodeling, affecting tumor growth, metastasis, and therapy resistance [754,755,756]. CAFs stimulate BC cells by secreting TGF-β1. It has been shown that inhibiting TGF-β1 suppresses tumor development [757].

IL-1β, overexpressed in CAFs, activates Wnt signaling, which increases IL-1β expression through feedback mechanisms to promote the growth and aggressiveness of BC cells. Inhibition of Wnt signaling has been shown to suppress the pro-tumorigenic functions of CAFs [758]. CAFs also support cancer cells by regulating their metabolism. Through autophagy, CAFs can regulate the glycolysis level in BC cells and influence their growth. CAFs can also produce lactate, which cancer cells utilize for energy [759].

CSC are a significant contributor to the development of both NMIBC and MIBC. CSCs are capable of regenerating diverse subpopulations of tumor cells, thereby sustaining tumor heterogeneity. This heterogeneity contributes to treatment resistance and elevates the risk of relapse. The presence of CSCs in BC is significantly associated with disease aggressiveness. These cells produce a plethora of molecular signals that stimulate tumor proliferation and invasion, and also modify the tumor microenvironment, thereby facilitating further disease progression. In particular, CSCs can initiate processes such as angiogenesis (the formation of new blood vessels), which provide the tumor with a source of nutrients and support its growth [760,761].

#### 3.15.2. Immune Cells in Bladder Cancer

In bladder cancer, T cell profiles include CD4+ T cells, cytotoxic CD8+ T cells, and Tregs. CD8+ T cell abundance positively correlates with long-term survival. Notably, T cell prognostic value depends on their localization: high numbers in the epithelial layer and peristomal regions indicate longer survival, whereas high stromal infiltration correlates with shorter survival. In advanced urothelial cancer, T cells are primarily restricted to the tumor stroma [753,762].

In BC, Tregs were attracted to the tumor mass by TAM M2-derived cytokines/chemokines. They cooperate with T cells and DCs—and their high number in urine correlates with rapid recurrence after the therapy with intravesical instillation of Bacillus–Calmette–Guerin (BCG), so they can be used as a marker of therapy efficacy [730].

B cell infiltration in bladder cancer holds prognostic value. High B cell numbers in the tumor mass correlate with longer survival and better clinical outcomes, particularly when they exhibit a memory B cell or plasma cell phenotype [763].

In bladder cancer, a high number of dendritic cells correlates with poor response to BCG-based therapy. As key regulators of T cell activation, DCs determine the effectiveness of immune responses against cancer antigens. In non-muscle invasive bladder cancer (NMIBC), high numbers of CD83+ DCs are linked to increased progression to muscle invasive bladder cancer (MIBC) [764].

Additionally, patients with elevated CD83+ DCs exhibit poorer recurrence-free survival after BCG therapy. CD83 is highly expressed on activated DCs and involved in upregulating T cell cytotoxic markers, and it may serve as a predictive marker for recurrence-free survival in elderly patients [764].

In BC, tumor cells recruit MDSCs and iDCs promoting T cell tolerance to cancer antigens to reduce excessive inflammation. In bladder cancer, CD33+ MDSCs are significantly elevated in the tumor mass, primarily as eMDSCs (CD33+DR-CD15-CD14-). High CD33+ MDSC levels correlate with rapid progression and shorter survival. Their elevated presence in urine and blood also associates with poor prognosis [765,766].

A study conducted in 2023 demonstrated a correlation between the presence of neutrophils and high-grade urothelial cancer. Furthermore, a notable elevation in neutrophil levels was observed in MIBC relative to NMIBC in high-grade malignancies. Increase in neutrophil counts in high-grade and deeply invasive tumors may indicate a correlation between TANs and poorer prognosis. A similar correlation was reported in localized bladder cancer, whereby higher TAN counts were associated with deeper tumor invasion and higher grade of malignancy. A significant elevation in lymphocyte levels was documented during the progression from MIBC to NMIBC in both low-grade and high-grade tumors. This finding is consistent with the results of previous studies that have identified an association between the adaptive immune response and tumor progression. Tissue-level studies have linked the neutrophil-to-lymphocyte ratio (NLR) to tumor stage, with significantly higher levels in high-grade tumors compared to low-grade ones. Moreover, NLR is notably elevated in MIBC versus NMIBC) in high-grade cases, reinforcing neutrophils role in tumor promotion. Tumor-associated neutrophils are proposed as a distinct population from circulating neutrophils, shaped by tumor microenvironment cytokines to acquire a pro-tumor phenotype. These TANs suppress CD8+ T cell activity, with their tumor-promoting effects intensifying as the disease progresses [131,767,768,769].

In NMIBC, TAM infiltration in the bladder wall is significantly reduced compared to normal bladder tissue. Studies across NMIBC and MIBC cohorts have shown a positive correlation between TAM numbers, identified by the pan-macrophage marker CD68, and both higher pathological T grades and advanced tumor stage. Wang et al. emphasized the importance of M2 macrophage localization, finding a positive correlation between their stromal density and tumor stage, except within the tumor core. Large-scale RNA sequencing from the Cancer Genome Atlas MIBC cohort further supports the link between TAMs, particularly M2 phenotype TAMs, and bladder cancer progression. Additionally, a genetically engineered mouse model of bladder cancer confirms that TAM numbers, including M2 macrophages, increase with tumor progression [770,771,772].

### 3.16. Skin Cancer

There are three main types of skin cancer: basal cell carcinoma (BCC), squamous cell carcinoma (SCC) and melanoma. BCC and SCC originate from epidermal keratinocytes, while melanoma originates from melanocytes, which are pigment-producing cells, primarily located in the basal layer of the epidermis, the choroidal layer of the eye, the inner ear, and the leptomeninges. Melanoma is the most aggressive and deadly skin cancer [773,774,775].

It is the fifth most common cancer in men and the sixth in women, and it is considered a multifactorial cancer, depending on both genetic susceptibility and environmental exposure. The most important environmental risk factor for melanoma development is exposure to UV rays. At the same time, the most significant host risk factors include the number of melanocytic nevi (benign accumulations of melanocytes or nevus cells) and a family history, as well as a personal history of other adult malignancies, such as brain, breast, or prostate cancer. Importantly, the total number of nevi positively correlates with the risk of melanoma development. A group of people particularly at risk for melanoma includes patients with red or blond hair and many freckles, as well as patients with darker hair and skin who have a high count of melanocytic nevi. Additionally, immunosuppressive therapy (e.g., TNF-α administered for longer than 120 days), obesity in men, low levels of vitamin D, and Parkinson’s disease are considered potential risk factors for melanoma development [776,777].

Melanoma cancer cells interact with the surrounding TME, leading to a less immunogenic phenotype and the ability to suppress anti-cancer immune cells.

#### 3.16.1. CAFs in Skin Cancer

In melanoma, there is a specific subset of CAFs known as melanoma-associated fibroblasts (MAFs) which undergo adaptations to meet the needs of melanoma cells (Figure 4). MAFs are characterized by increased expression of IGF1, VEGFA, FGF2, SDF1, IL-6, IL-8, CCL2, CXCL12, sFRP2, hyaluronan and proteoglycan link protein 1 (HAPLN1), and connective tissue growth factor (CTGF), which promote cancer cell survival, proliferation, and drug resistance. MAFs are activated by melanoma cells through the secretion of cytokines and chemokines such as IL-6, FGF, and IL-8. In melanoma, normal keratinocytes cooperate with cancer cells in transforming fibroblasts into MAFs. In turn, MAFs secrete substances that promote proliferation, invasion, metastasis, and angiogenesis. The interaction between melanoma cells and MAFs creates a specific microenvironment favoring tumor growth [778,779,780,781,782,783,784].

MAFs influence the creation of an immunosuppressive microenvironment by increasing TGF-β, CCL2, IL-6, GM-CSF, MMP, PGE2, COX2, CXCL5, PDL-1, and PDL-2. MAFs can modify the availability of important immunomodulatory metabolites in the TME, such as lactate, glucose, and arginine, which may contribute to immune cell suppression or transformation into pro-tumor phenotypes. MAFs increase the level of IL-10 secreted by M2 macrophages in a manner dependent on cyclooxygenase/indoleamine 2,3-dioxygenase. MAFs cause the release of chromatin-associated nuclear DNA and cytotoxic granules by neutrophils through a ROS-dependent mechanism driven by CD11b. This leads to the formation of extracellular traps, which in turn enhance MAFs activation and promote tumor growth. IL-6 secreted by MAFs induces the invasiveness of melanoma cells; however, the exact mechanism has not been elucidated. The level of IL-6 production is significantly influenced by the hypoxic microenvironment. CXCL5 secreted by MAFs activates the PI3K/AKT signaling pathway in melanoma cells, which affects PD-L1 expression and promotes immune escape. MAFs inhibit the activity of CD8+ T cells by activating Snail-1, which affects FAP expression and promotes the development of an immunosuppressive microenvironment shaped by Tregs. Additionally, chemokines such as CXCL1, CXCL2, CXCL5, CCL2, and CCL3, produced by MAFs, play a role in attracting pro-tumor MDSCs to inhibit CD8+ cells [781,783,785,786,787,788,789,790,791].

CAFs associated with BCC exhibit increased expression of cathepsin K (CTSK), matrix Gla protein (MGP), cartilage intermediate layer protein (CLIP), dermatopontin (DPT), secreted frizzled-related protein 2 (SRFP2) and platelet-derived growth factor receptor-like protein ligand (PDGFRL) [792].

#### 3.16.2. Other Non-Immune Cells in Melanoma

TECs promote cancer cell survival, proliferation and migration by activating NF-κB in melanoma cells in a galectin-9-independent manner. This occurs through the upregulation of the T cell inhibitory receptor, T cell immunoglobulin, and mucin domain-3 (TIM-3) on the surface of TECs by TGF-β [793].

Adipocytes from the subcutaneous tissue are recruited by melanoma cells to promote tumor growth by providing lipids to tumor cells and inducing metabolic reprogramming. At the same time, diet-induced obesity increases melanoma progression and impacts on the lymphangiogenesis and lymph node metastasis [794].

Furthermore, cancer-associated adipocytes undergo conversion to CAFs, modifying the TME and facilitating melanoma progression. In the co-culture of CAAs and melanoma cells, dedifferentiation of adipocytes towards fibroblast-like phenotype (higher expression of collagen, MMPs, and α-SMA) is observed and such modified adipocytes promote melanoma cell migration through the Wnt5a pathway. The cross-talk between CAAs and melanoma cancer cells may be increased in obesity, due to imbalance of adipokine secretion [795,796].

Adipocytes in obese patients with melanoma release high amounts of pro-inflammatory cytokines and growth factors (IL-6, IL-32 or TNF-α) and molecules involved in the angiogenesis enhancement (OPN—osteopontin, chemerin, apelin and PAI-1) that modulate melanoma proliferation [795].

Growing body of evidence supports the presence of cancer stem cells (CSCs) in melanoma, with a well-documented role in tumor recurrence. CSC resistance to chemotherapy contributes to the failure of traditional anti-cancer drugs to fully eradicate tumors, allowing for tumor inhibition but increasing the risk of metastasis and relapse. CSCs express various biomarkers, including CD34, aldehyde dehydrogenase 1 (ALDH1), CD271, CD44, and lysine demethylase 1B (JARID1B); however, none have been confirmed as exclusive CSC markers [797,798,799].

#### 3.16.3. T Cells in Melanoma

In melanoma patients, CD4+ T cells and dendritic cells (particularly CD123+ pDCs) contribute to spontaneous tumor regression. However, cytotoxic T lymphocytes play a key role, targeting melanoma-associated antigens (MAGE—melanoma-associated antigen, BAGE—B melanoma antigen, GAGE—cancer/testis antigen, PRAME—preferentially expressed antigen of melanoma, and NY-ESO-1—New York esophageal squamous cell carcinoma), melanocyte differentiation markers (tyrosinase, Melan-A/MART1, gp100, and TRP-1, TRP-2—tyrosinase-related protein 1 or 2), and mutated or aberrantly expressed antigens (MUM-1—melanoma-associated antigen 1, CDK4, β-catenin, gp100-in4, p15, and N-acetylglucosaminyl transferase V). Notably, Melan-A-specific CD8+ T cells in melanoma patients exhibit a naïve phenotype (CCR7+CD45RA+), a trait also observed in CD8+ T cells from cancer-free lymph nodes in this group [800,801].

#### 3.16.4. NK Cells in Melanoma

In melanoma, NK cells are recruited to the tumor mass by inflammatory cytokines such as CCL5 and CXCL9-11, expressed by other TME cells. However, NK cell infiltration is typically low, with localization restricted to the stroma, without direct contact with tumor cells. Their primary role is eliminating circulating cancer cells, thereby limiting metastasis. In melanoma patients, the predominant infiltrating NK cells exhibit a CD56^dim^CD16- phenotype. NK cell numbers and cytotoxic activity decline in metastatic melanoma, correlating with disease progression. High percentage of NK cells suppresses cancer development and improves the overall survival [802,803].

NK cells secrete high levels of IFN-γ and IL-4 upon recognizing αGalCer presented by the non-classical MHC molecule CD1d. Notably, CD1d also presents GD3, a melanoma-specific antigen, potentially contributing to the anti-cancer immune response. NK cells from melanoma patients are functionally impaired, and TIM-3 blockade acts in opposition to the T cell exhaustion phenotype. Additionally, the expression of TIM-3 correlates with the stage of melanoma and poor prognosis [801,804].

#### 3.16.5. B Cells in Melanoma

In melanoma, B cells are primarily localized in the tumor stroma, constituting 10% of tumor-infiltrating lymphocytes. CD20+ and CD22+ B cell levels are significantly higher in melanoma patients than in healthy individuals, with their primary role being antibody production against melanoma-associated antigens. Plasma cells in melanoma exhibit polyclonality, predominantly expressing IgG and IgA, which may enhance anti-cancer immune responses [805,806].

Conversely, Bregs contribute to immune evasion, with their high numbers negatively correlating with survival. They produce IL-10 and suppress T cell responses, promoting cancer progression [633,807].

#### 3.16.6. Monocyte-Related Cells in Melanoma

In melanoma patients, DC subsets regulating T cell cytotoxicity influence prognosis. Impaired cancer-antigen presentation by DCs correlates with worse outcomes. Common DC dysfunctions include upregulation of CTLA-4 and PD-1 receptors. DCs circulate in the bloodstream but also infiltrate tumor margins. Melanoma is predominantly infiltrated by cDC1s, characterized by BDCA-3 (CD141) expression. Additionally, Langerhans cells, a subset of epidermal antigen-presenting cells, exhibit both functional and numerical impairments, contributing to melanoma progression [808,809,810,811].

In melanoma, the accumulation of monocyte-derived MDSCs is linked to the blockade of DC maturation. In melanoma-bearing mice, B-Raf protooncogne (BRAF) inhibitors prevent their recruitment into the TME, while MAPK/ERK kinase (MEK) inhibitors suppress monocyte polarization into MDSCs and MDSC infiltration. Melanoma cells secrete various factors that promote the development of MDSCs, Tregs, CAFs, and TAMs, with MDSCs playing the predominant immunosuppressive role. MDSCs highly express ARG1 and PD-L1 and secrete large amounts of nitric oxide species (NOS) and ROS, effectively inhibiting T cells in both in vitro and in vivo conditions. In advanced melanoma, high concentrations of inflammatory mediators such as IL-1β and IFN-γ promote MDSCs accumulation and activation. Increased peripheral mo-MDSCs and PMN-MDSCs correlate with tumor burden in patients with malignant melanoma.

MDSCs also influence proliferation and vasculogenic mimicry formation of melanoma and participate in processes mediated by VEGF/VEGFR. Additionally, a subset of mo-MDSCs in melanoma patients overexpresses PD-L1, CD73, and pro-inflammatory cytokines, likely contributing to immune evasion [812,813,814].

#### 3.16.7. Macrophages in Melanoma

Macrophage polarization to the M1 type has been demonstrated to exert an inhibitory effect on the proliferation of melanoma cells. The elevated expression of connexin 43, a pivotal gap junction protein within the TME, induces M1 polarization, thereby impeding melanoma cell invasion and migration in vitro. Conversely, augmented numbers of M2 macrophages facilitate melanoma growth. Furthermore, macrophages deficient in β3 integrin have been observed to induce M2 macrophage polarization, which facilitates melanoma growth [780,815,816,817].

### 3.17. Osteosarcoma

Osteosarcoma is the most common primary bone cancer and the third most frequent pediatric malignancy, with a high lethality rate. Patients with advanced or metastatic disease have limited treatment options. Its low incidence and genetic heterogeneity hinder the identification of common genetic drivers, leaving its exact etiology unclear. Consequently, despite decades of research, patient survival rates have seen little improvement. Osteosarcoma primarily affects the long bones near the most active growth plates, including the distal femur, proximal tibia, and proximal humerus. Key symptoms, depending on tumor location and growth rate, include restricted joint movement, localized pain, and edema around the tumor mass [818,819,820,821].

#### 3.17.1. CSCs in Osteosarcoma

Recent data suggest that osteosarcoma tumor cells interact with bone marrow niche cells via direct contact or paracrine signaling (using soluble growth factors or extracellular vesicles), promoting apoptosis resistance through Notch activation. Notably, Notch activation via its ligand Jagged1 enhances osteosarcoma cell proliferation, drug resistance, and metastasis formation. Doxorubicin and cisplatin can activate the Notch pathway. At non-toxic doses, doxorubicin inhibits osteosarcoma cell proliferation by upregulating target genes such as Hes-related family BHLH transcription factor (*HEY1*), *NOTCH1*, *HES1*, and *HES5*. Meanwhile, sub-lethal cisplatin doses select for a resistant mesenchymal-like subset expressing osteosarcoma CSC markers (STRO-1/CD117), a phenotype reversible by γ-secretase inhibition [822,823,824].

#### 3.17.2. Neutrophils in Osteosarcoma

Extensive research indicates that neutrophil extracellular traps significantly impact the tumor immune microenvironment by promoting tumor migration, invasion, and metastasis through various mechanisms. NETs shield tumor cells from antitumor immune cells, including NK and CD8+ T cells, influencing the immune landscape and immunotherapy response. However, their role in osteosarcoma metastasis remains unstudied. Conversely, neutrophils can also enhance antitumor responses by directly eliminating osteosarcoma cells [825,826,827].

#### 3.17.3. Macrophages in Osteosarcoma

CD14+/CD68+ tumor-associated macrophages are the predominant immune cells infiltrating osteosarcoma. Gene expression analysis and CD209 staining confirm an elevated presence of M2 macrophages in osteosarcoma tissues. The disease’s progression may disrupt macrophage subtype balance. An independent study reported a higher prevalence of CD163+ macrophages in tumor-infiltrating cells from resected tumors compared to peripheral blood, promoting T lymphocyte proliferation and proinflammatory cytokine production. Macrophage infiltration varies in metastatic osteosarcoma. Han et al. found significantly higher CD68 expression in metastatic osteosarcoma than in non-metastatic cases, with increased CD68 levels in cancer metastases versus primary lesions. Dumars et al. identified a higher prevalence of INOS+ M1 macrophages in non-metastatic tumors. In a mouse model, macrophages were recruited to tumor tissue and polarized into the M2 subset. A significant number of F4/80+ macrophages infiltrated metastasis in lung tissue, with increased M2-type (CD206+MHC-II−) macrophages but no change in M1-type (CD206−MHC-II+) levels. These findings suggest distinct roles for M1 and M2 macrophages in osteosarcoma progression. RNA-seq analysis linked elevated M1 and M2 macrophage levels to improved overall survival in patients with favorable prognoses. Similarly, a bioinformatic study correlated M0 macrophages with better outcomes. Gomez-Brouchet et al. reported a significant association between increased CD163 TAMs and prolonged metastasis progression-free survival (MPFS) and overall survival, with a similar but non-significant trend for CD68-positive cells [828,829,830,831,832,833,834,835,836].

## 4. TME-Targeting Therapies

Recent studies clearly indicate that successful cancer treatment relies not only on eliminating cancer cells but also on inhibiting the pro-tumor effects of the surrounding microenvironment. Consequently, significant efforts are being directed towards developing TME-targeting therapies. These therapies aim to reshape the TME composition and eliminating signals that sustain cancer cells. The most effective TME-targeting strategies include immunotherapies, antiangiogenic medicines, CAFs and the ECM treatments, including cold atmospheric plasma, oncolytic viral therapy, bacterial therapy, nanovaccines, and repurposed pharmaceuticals with a combination therapy. However, the TME is dynamic, undergoing continuous changes throughout cancer progression and treatment, which affect treatment response [2]. Below, some of these therapies will be discussed, along with the impact of other TME cells on the effectiveness of these treatments.

Chemotherapy is one of the most widely used treatments for cancer, but it is not specifically designed to target the TME. Although, chemotherapy impacts the TME in multiple ways, extending beyond its direct cytotoxic effects on tumor cells. Certain chemotherapeutic agents can induce immunogenic cell death, leading to the release of danger-associated molecular patterns (DAMPs) that stimulate antitumor immune responses. Moreover, chemotherapy influences on TAMs, modulating their recruitment, depletion, and polarization within the TME. These changes can either enhance or suppress tumor growth, depending on the specific cellular and molecular context of the tumor. Additionally, some chemotherapeutic agents, such as doxorubicin and gemcitabine, have been shown to suppress MDSCs in preclinical models, potentially enhancing immune responses against tumors. However, due to the heterogeneity of MDSCs, their clinical targeting remains challenging [119,837,838].

Among TME-targeting therapies, immunotherapy plays a pivotal role in reshaping the microenvironment by reversing immune suppression. Immune checkpoint inhibitors (ICIs) targeting PD-1 and PD-L1 restore T cell activity, allowing for a more effective immune-mediated tumor elimination. Nivolumab and pembrolizumab were the first monoclonal antibodies targeting PD-1, approved for cancer treatment. CTLA-4 is another major target for ICIs. This co-inhibitory molecule binds B7-1/B7-2, suppressing T cell activation. CTLA-4 blockade, using antibodies such as ipilimumab, has shown efficacy in cancer treatment. However, limited efficacy has been observed in certain cancers due to the presence of immunosuppressive cells, including MDSCs and Tregs that induce CD8+ T cell anergy. Bavituximab, a monoclonal antibody targeting phosphatidylserine, is being evaluated in some cancers for its ability to inhibit MDSC differentiation. Other emerging targets of ICIs include LAG-3 or TIM-3, which modulate immune responses. However, the mentioned above immunosuppressive cells can interfere with the effectiveness of these therapies. Moreover, CAFs, by creating a physical barrier (through the production of ECM components) and releasing immunosuppressive cytokines, can counteract the effects of checkpoint blockade, making it necessary to combine checkpoint inhibitors with therapies that target other components of the TME [838,839,840,841,842,843,844,845].

Other immunotherapies, such as CAR-T cell therapy (chimeric antigen receptor), provide a more targeted approach by engineering T cells to recognize and destroy cancer cells expressing specific markers. While CAR-T therapy has demonstrated significant success in hematologic malignancies, its application in solid tumors remains limited due to TME-mediated resistance mechanisms, including the immunosuppressive effects of TAMs, MDSCs, and Tregs [846,847,848,849,850,851,852,853,854].

Therefore, depletion strategies are being explored, e.g., targeting IL-2, a key regulator of Treg survival. Alternative strategies involve Treg depletion using chemotherapeutics such as sunitinib and sorafenib, or anti-CD25 antibodies [855,856].

TAMs are being targeted through, e.g., CSF1R inhibitors and repolarization towards the M1 phenotype. Nanoparticles, immunosuppressive agents, and phosphoric acid compounds have shown potential in TAM-targeted therapies. More radical approaches include direct macrophage depletion using bisphosphonates or trabectedin [839,857,858].

Adoptive transfer of TCR-T cells, using next-generation sequencing (NGS) for identification of specific TCR sequences, offers another immunotherapeutic avenue. TCR-T cells recognize cancer-specific antigens via HLA-restricted interactions and may be particularly useful in solid tumors. Oncolytic viruses (OVs), including adenoviruses, herpesviruses, and Newcastle disease viruses, selectively infect and lyse cancer cells while stimulating antitumor immunity. Clinical trials are evaluating their efficacy in hematologic malignancies and solid tumors, including melanoma, prostate cancer, glioblastoma, and lung cancer [859,860,861,862,863,864].

The intricate interactions between cancer cells and the TME play a crucial role in determining therapeutic responses. While immunotherapies such as CAR-T and ICIs have revolutionized treatment, their efficacy in solid tumors remains limited due to the dynamic nature of the TME. A comprehensive understanding of the cellular composition of the TME in each cancer type is essential to optimize these therapeutic strategies, enabling the precise identification of targets and the development of more effective combination approaches.

### Tumor Microenvironment Composition Across Cancer Types—A Transcriptomic Perspective

In a seminal paper, Bagaev et al. used transcriptomic analysis to identify four TME subtypes that are conserved across multiple cancer types in over 10,000 patients. Their study provided insights into the therapeutic implications of the varying cellular compositions of the TME [865].

These subtypes are as follows:

1. Immune enriched, fibrotic (IE/F)—characterized by high immune infiltration but also enriched with fibroblasts and pro-fibrotic signaling;
2. Immune enriched, non-fibrotic (IE)—characterized by strong immune cell presence with minimal fibroblast activity, associated with high immunogenicity;
3. Fibrotic (F)—with low immune infiltration, predominantly fibroblast-rich with angiogenic signatures;
4. Immune depleted (D)—composed by scarce immune and stromal cells, with a high malignant cell fraction [865].

The proportions of key cell populations in each TME subtype are summarized in Table 2.

In the context of clinical implications, IE tumors respond well to immune checkpoint blockade (e.g., anti-PD-1 and anti-CTLA-4), while IE/F tumors may require combination therapies targeting fibroblast-mediated immune suppression (e.g., anti-TGF-β). F tumors benefit from stroma-targeting therapies (e.g., VEGF inhibitors), whereas D tumors exhibit poor immune infiltration and may require alternative strategies such as chemotherapy or metabolic interventions [865].

## 5. Conclusions

The tumor microenvironment is a highly structured and dynamic ecosystem, composed of cellular components, extracellular matrix proteins, and signaling molecules that create complex bidirectional interactions with cancer cells. Cancer cells actively modify the TME to optimize conditions for survival, growth and metastasis. In response to challenges such as oxygen deprivation, nutrient deficiency, waste accumulation, and chemotherapy, they recruit fibroblasts, adipocytes, immune cells, and other cells that work in concert to support tumor progression.

In recent decades, a wealth of scientific research has elucidated the diverse roles of cells within the TME, revealing their multifaceted interactions and contributions to both tumorigenesis and metastasis. For example, CAFs promote cancer cell proliferation and invasion by secreting ECM proteins and growth factors. Endothelial cells play a central role in angiogenesis within the TME. The aberrant vasculature in tumors, characterized by leaky and irregular blood vessels, can lead to cancer cell dissemination, further contributing to metastasis. Adipocytes, particularly in adipose-rich tumors such as breast cancer, can release fatty acids and adipokines that promote tumor cell proliferation, survival, and migration. Similarly, immune cells, such as macrophages, can be polarized to a pro-tumorigenic phenotype that facilitates immune suppression and enhances cancer cells survival. However, immune cells within the TME can have both pro-tumor and antitumor effects, depending on their phenotype and activation state. Understanding the complex cellular and molecular interactions within the TME is essential for designing more effective therapeutic interventions. Importantly, we must recognize that the composition and functional state of the TME vary depending on cancer type, tumor stage, patient characteristics, and prior treatments. This heterogeneity highlights the need for personalized therapeutic strategies that target specific TME components to improve outcomes of the treatment.

## Figures and Tables

**Table 1:** Specific markers and processes regulated by different subtypes of CAF in tumor microenvironment.

CAF Subtype	Specific Markers	Functions
matrix CAFs	MMP11, COL10A1, COL11A1, COL8A1, COL1A2, COL12A1, COL3A1, COMP, POSTN, LRRC15, LRRC17, ASPN, SULF1, INHBA, VCAN, TGF-β, KRAS	ECM remodeling, collagen deposition, tissue stiffness, tumor invasion
(mCAFs)		
myofibroblastic CAFs (myCAFs)	TAGLN, MYL9, TPM1, TPM2, MMP11, POSTN, αSMA, COL1A1	ECM contraction, mechanical force transmission, tumor progression
inflammatory CAFs (iCAFs)	IL-6, IL-8, CXCL1, CXCL2, CCL2, CXCL12, CFD, LMNA, DPT, HAS1, HAS2, AGTR1, PLA2G2A	Inflammatory cytokines secretion, immune cell recruitment, tumor-promoting inflammation
vascular CAFs (vCAFs)	NOTCH3, COL18A1	Angiogenesis
tumor-like CAFs (tCAFs)	PDPN, MME, TMEM158, NDRG1, ENO1, GAPDH, VEGFA	Tumor growth, metabolic support, immune evasion
heat shock protein tumor-like CAFs (hsptCAFs)	HSPH1, HSP90AA1, PDPN, MME, TMEM158, NDRG1, ENO1, GAPDH, VEGFA, TGF-β, KRAS, MTORC1	Stress response, therapy resistance, tumor survival
interferon-responsive CAFs (ifnCAFs)	IL-32, CXCL9, CXCL10, CXCL11, IDO1, TAT5, TNF-α, IL-6, KRAS	Immune signaling, T cell response, immune suppression
antigen-presenting CAFs (apCAFs)	HLA-DRA, HLA-DRB1, CD74	Interactions with immune cells, potential role in antigen presentation, immune modulation
reticular CAFs (rCAFs)	CCL21 and CCL19	Lymphoid-like structure formation, immune response, T cell recruitment
division CAFs (dCAFs)	TUBA1B, MKI67	Cell division, tumorigenesis, tumor growth and survival
metabolic CAFs (meCAFs)	PLA2G2A	Energy supply to tumor cells, adaptation to hypoxia

**Table 2:** Cellular composition of TME subtypes.

Cell Type	IE (%) *	IE/F (%) *	F (%) **	D (%) **
CD8+ T Cells	20–30	10–20	<5	<5
CD4+ T Cells	10–15	5–10	<5	<5
Tregs	5–10	5–10	<5	<5
Macrophages (M1)	10–15	5–10	<5	<5
Macrophages (M2)	<5	10–15	10–20	<5
Fibroblasts	<5	15–25	40–50	<5
Endothelial Cells	<5	5–10	20–30	<5
Cancer Cells	30–40	30–40	30–40	>60

* Dominant in melanoma, bladder cancer, and gastric cancer. ** More common in pancreatic cancer, glioblastoma, and some lung adenocarcinomas.

## Data Availability

Not applicable.

## Abbreviations

ABC—adenosine triphosphate (ATP)-binding cassette transporter, ACLY—ATP (adenosinotrifosforan) citrate lyase, ADAMs—disinteegrins and metalloproteinase, ADCC—antibody-dependent cellular cytotoxicity, ADH1B—alcohol dehydrogenase 1B, ADSC—adipose-derived stem cell, AGR2—anterior-gradient 2, AGTR1—angiotensin II type 1 receptor, Akt—serine-threonine protein kinase AKT1, ALDH1—aldehyde dehydrogenase isozymes, ALDH—aldehyde dehydrogenase family, AML—acute myeloid leukemia, ANXA3—annexin A3, APCs—antigen-presenting cells, apCAFs—antigen-presenting cancer-associated fibroblasts, ARCD—acquired renal cystic disease, ARG-1—arginase-1, α-SMA—alpha smooth muscle actin, ASPN—asporin, ATC—anaplastic thyroid cancer, AXL—tyrosine kinase receptor, BAGE—B melanoma antigen, BAT—brown adipose tissue, BC—breast cancer, BC—bladder cancer, BCC—basal cell carcinoma, BCG—Bacillus–Calmette–Guerin, Bcl-2—B cell lymphoma 2, BCR—B cell receptor, BDNF—brain-derived neurotrophic factor, BHP-1—immortalized benign prostatic hyperplasia cell line, BIMP-1—B lymphocyte-induced maturation protein-1, BL1—type basal-like 1, BMI—body mass index, BMI1—B cell-specific Moloney murine leukemia virus integration site 1 gene, BMSCs—bone marrow stromal cells, BRAF—B-Raf protooncogene, BRCA-1—breast cancer gene 1, Bregs—B regulatory cells, BTK—Bruton’s tyrosine kinase, CAAs—cancer-associated adipocytes, CAF—cancer-associated fibroblasts, cAMP—cyclic adenosine monophosphate, CAR—chimeric antigen receptor, CAV1—caveolin—1, ccRCC—clear cell renal cell carcinoma, CCRK—oncogenic cell cycle-related kinase, cDCs—classical dendritic cells, CD—cluster of differentiation, CDKN2A—cyclin-dependent kinase inhibitor 2A, C/EBPα—CCAAT/enhancer-binding proteins, CFD—complement factor D, CKD—chronic kidney disease, CLEVER-1—common lymphatic endothelial and vascular endothelial receptor 1, CLIP—cartilage intermediate layer protein, c-MET—receptor tyrosine kinase, CNS—central nervous system, CMP—common myeloid progenitor, CNS—central nervous system, COL—collagen, COL11A1—collagen type XI alpha 1, COMP—non-collagenous matrix protein, COX–2—cyclooxygenase 2, CRC—colorectal cancer, CREB3L1—cyclic adenosine monophosphate (cAMP) response element-binding protein 3 like, CRPC—castration-resistant prostate cancer, CSCs—cancer stem cells, CSF-1—colony-stimulating factor 1, CSF1R—colony-stimulating factor 1 receptor, CTAG2—cancer-testis antigen 2, CTGF—connective tissue growth factor, CTHRC1—triple helix repeat-containing 1, CTL—cytotoxic T cells, CTLs—chronic lymphocytic thyroiditis, CTLA-4—cytotoxic T lymphocyte associated antigen 4, CTSK—cathepsin K, CXCL—C-X-C motif ligand 1, D—immune-depleted tumor microenvironment subtype, DAMP—danger-associated molecular patterns, dCAF—division cancer-associated fibroblasts, DCs—dendritic cells, DCRegs—regulatory dendritic cells, DFS—disease-free survival, DNAM1—Dnax accessory molecule-1, DPC4—deleted in pancreatic cancer-4, DPP4—dipeptidyl peptidase-4, DPT—dermatopontin, DTC—differentiated thyroid cancer, E2F2—E2F transcription factor, EAC—esophageal adenocarcinoma, EBV—Epstein–Barr virus, EC—esophageal cancer, ECs—endothelial cells, ECM—extracellular matrix, EGFL7—epidermal growth factor-like domain-containing protein 7, EGFR—epidermal growth factor receptor, eMDSCs—early myeloid-derived suppressor cells, EMR1—epidermal growth factor (EGF)-like module-containing mucin-like hormone receptor-like 1, EMT—epithelial–mesenchymal transition, ENO1—alpha-enolase, EpCAM—epithelial cell adhesion molecule, EPH—ephrin, ER—estrogen receptor, ERK—extracellular signal-regulated kinase, ESCC—esophageal squamous cell carcinoma, ESRD—end-stage renal disease, ET-1—endothelin-1, F—fibrotic tumor microenvironment subtype, FABP4—fatty acid-binding protein 4, FAK—focal adhesion kinase, FAP—fibroblast activation protein, FAS—fatty acid synthase, FASL—CD95 (APO-1) ligand, FBP-1—fructose 1,6-bisphosphatase, FcγRIIIA—low-affinity receptor for Fc portion of immunoglobulin G, FFA—free fatty acid, FGF2—fibroblast growth factor 2, FGL2—fibrinogen-like protein 2, FLC—fibrolamellar hepatocellular carcinoma, FLNA—filamin A, FOSB—Finkel–Biskis–Jinkins (FBJ) murine osteosarcoma viral oncogene homolog G, FOXO3—forkhead box 3, FoxP3—forkhead box protein P3, FSP-1—ferroptosis suppressor protein 1, FTC—follicular thyroid cancer, GAG—glycosaminoglycan, GAGE—cancer/testis antigen, GA-MSCs—glioblastoma-associated mesenchymal stem/stromal cells, GAPDH—glyceraldehyde 3-phosphate dehydrogenase, GAS6—growth arrest-specific 6, GATA3—GATA-binding protein 3, GBM—glioblastoma, GC—gastric cancer, GCSC—gastric cancer stem cells, GFAP—glial fibrillary acidic protein, GIST—gastrointestinal stromal tumor, GM-SCF—granulocyte-macrophage colony-stimulating factor, GPER—G-protein-coupled estrogen receptor 1, GR1—a glycosylphosphatidylinositol-linked myeloid differentiation marker, GRP78—glucose-regulated protein 78, GSK3β—glycogen synthase kinase-3, HAPLN1—hyaluronan and proteoglican link protein 1, HAS—hyaluronan synthases, HBV—hepatitis B virus, HCARs—hydroxycarboxylic acid-binding receptors, HCC—hepatocellular carcinoma, HCC-CCA—mixed hepatocellular cholangiocarcinoma, HCV—hepatitis C virus, HER—human epidermal receptor, HEY1—Hes-related family BHLH transcription factor, HGF—hepatocyte growth factor, HGSOC—high-grade serous ovarian cancer, HIF1α—hypoxia-inducible factor 1α subunit, HLA—human leukocyte antigen, HMGB1—high mobility group box 1 protein, HNC—head and neck cancer, HNSCC—head and neck squamous cell carcinoma, HO-8910—poorly differentiated ovarian papillary serous cyst-adenocarcinoma cell line, HOTAIR—HOX transcript antisense RNA, HOX—homeobox gene family, HPV—human papillomavirus, HSC—hepatic stellate cell, HSPG2—heparan sulfate proteoglycan 2, HSPH1—heat shock protein family H, hsptCAFs—heat shock protein tumor-like cancer-associated fibroblasts, IBD—inflammatory bowel disease, iCAFs—inflammatory cancer-associated fibroblasts, ICAM-1—intercellular adhesion molecule 1, iCCA—intrahepatic cholangiocarcinoma, ICIs—immune checkpoint inhibitors, ICOSL—inducible costimulator ligand, IDC—invasive ductal carcinoma, IDCs—immature dendritic cells, IDO1—indoleamine 2,3-dioxygenas, IE—immune-enriched, non-fibrotic, tumor microenvironment subtype, IE/F—immune-enriched, fibrotic, tumor microenvironment subtype, IER3—immediate early response 3, IF1—insulin-like growth factor 1, IFN-γ—interferon gamma, ifnCAFs—interferon-responsive cancer-associated fibroblasts, infDCs—inflammatory dendritic cells, IGF1—insulin-like growth factor, IGFBP3—insulin-like growth factor-binding protein 3, IkB—I kappaB kinase, ILC—invasive lobular carcinoma, ILT2—immunoglobulin-like transcript 2, IM CAF—immunomodulatory cancer-associated fibroblast, INHBA—inhibin beta, ITGA5—integrin alpha 5, ITGAM—integrin alpha M, ITGB2—integrin beta 2, iTILs—intratumoral tumor-infiltrating lymphocytes, JAK—Janus kinases, JARID1B—lysine demethylase 1B, JNK—c-Jun N-terminal kinase, KDM6B—lysine demethylase 6B, KIR—killer cell immunoglobulin-like receptors, KLF6—Kruppel-like factor—6, KLRC1/2—killer cell lectin-like receptor ½, KRAS—Kirsten rat sarcoma virus, LAG-3—lymphocyte-activation gene 3, LAGE-1—lymphocyte-activation gene 3, LAMP-3—lysosomal associated membrane protein 3, LAR—luminal androgen receptor, LC—liver cancer, LCs—Langerhans cells, LCC—large cell lung cancer, LCN2—lipocalin 2, LDL—low-density lipoprotein, LIF—leukemia inhibitory factor, LMNA—lamin A, LNCaP—lymph node carcinoma of the prostate cell line, lncRNA—long non-coding RNA, LOX1—lectin-type oxidized LDL receptor 1, LPS—lipopolysacharide, LRRC—leucine-rich repeat containing protein family, LTB—lymphotoxin β, MAD2L1—mitotic arrest deficient 2 like 1, MAF—melanoma-associated fibroblasts, MAGE—melanoma-associated antigen, MAPK—mitogen-activated protein kinase, MART1—melanoma antigen recognized by T cells, mCAFs—matrix cancer-associated fibroblasts, MCP-1—monocyte chemoattractant protein-1, MCT4—monocarboxylate transporter 4, mDCs—mature dendritic cells, MDSC—myeloid-derived suppressor cells, meCAFs—metabolic cancer-associated fibroblasts, MEK—MAPK/ERK kinase, MGP—matrix Gla protein, MHC—major histocompatibility complex, MIBC—muscle-invasive bladder cancer, MIC-A—MHC class I polypeptide-related sequence A, MIF—migration inhibitory factor, miRNA—microRNA, MKI67—Ki-67 protein, MME—membrane metalloendopeptidase, MMP—matrix metalloproteinase, Mo-MDSCs—monocytic myeloid-derived suppressor cells, MPFS—metastasis progression-free survival, MR CAF—mechanoresponsive cancer-associated fibroblast, MSCs—mesenchymal stem cells, MSL—mesenchymal stem-cell like, MTC—medullary thyroid cancer, MU—metastatic unit, MUM-1—melanoma-associated antigen 1, MYC—protoonkogene, myCAFs—myofibroblastic cancer-associated fibroblasts, MYL—myosin light chain, MTORC1—mammalian target of rapamycin complex 1, mTOR—mammalian target of rapamycin, NAT—neck adipose tissue, NDRG1—N-myc downstream-regulated gene 1, NEAT1—nuclear paraspeckle assembly transcript 1, NET—neutrophil extracellular traps, NF1—neurofibromatosis type 1, NFkβ—nuclear factor kappa-light-chain enhancer of activated B cells, NGAL—neutrophil gelatinase-associated lipocalin, NGS—next-generation sequencing, NKG2A—natural killer group 2 member A protein, NKG2D—natural killer group 2 member D protein, NLR—neutrophil-to-lymphocyte ratio, NMIBC—non-muscle-invasive bladder cancer, NO—nitric oxide, NOD/SCID—severe combined immune deficiency, NOTCH3—neurogenic locus notch homolog protein, Nrf2—nuclear factor erythroid 2-related factor 2, NSCLC—non-small-cell lung cancer, NY-ESO-1—New York esophageal squamous cell carcinoma, CTAG1B—cancer/testis antigen 1, OC—ovarian cancer, OCT4—octamer-binding transcription factor 4, OIP5-AS1—OIP5-antisense RNA 1, Olig2—oligodendrocyte lineage-specific basic helix-loop-helix 2, OPN—osteopontin, OPSCC—oropoharyngeal squamous cell carcinoma, OS—overall survival, OSCC—oral squamous cell carcinoma, OSE—ovarian surface epithelium, OSM—oncostatin M, OXPHOS—oxidative phosphorylation, OV—oncolytic virus, PAD4—peptidyl arginine deiminase 4, PAI-1—plasminogen activator inhibitor –1, PALB2—partner and localizer of BRCA2, PanC—pancreatic cancer, PanNET—pancreatic neuroendocrine tumor, PAX6—paired box 6, PBDE—polybrominated diphenyl ether, PBMC—peroipheral blood mononuclear cell, PC—prostate cancer, PCSCs—prostate cancer stem cells, PDAC—pancreatic ductal adenocarcinoma, pDCs—plasmacytoid DCs, PDGF—platelet-derived growth factor, PDGFR-β—platelet-derived growth factor receptor β, PDGFRL—platelet-derived growth factor receptor-like protein ligand, PDK1—phosphoinositide-dependent protein kinase-1, PD-L1—programmed death ligand 1, PDPN—podoplanin, PDTC—poorly differentiated thyroid cancer, PFKFB2—6-phosphofructo-2-kinase/fructose-2,6-biphosphatase, PFS—progression free-survival, PGE2—prostaglandin E2, PGI2—prostacyclin 2, PI3K—phosphoinositide 3-kinase, PKA—protein kinase A, PLA2G2A—phospholipase A2 group IIA, PLAC9—placenta-specific 9, PMN-MDSCs—polymorphonuclear myeloid-derived suppressor cells, POSTN—periostin, PPARγ—peroxisome proliferator-activated receptor gamma, PR—progesteron receptor, PRAME—preferentially expressed antigen of melanoma, PSC—pancreatic stellate cell, PTC—papillary thyroid cancer, PTEN—phosphatase and tensin homolog deleted on chromosome 10, PTGS2 = COX2 (cyclooxygenase 2), RAF—seirine/threonine kinase proto-oncogene, Rb—retinoblastoma, rCAFs—reticular cancer-associated fibroblasts, RCC—renal cell carcinoma, RGD—arginylglycylaspartic acid, RFS—relapse-free survival, ROS—reactive oxygen species, S100A8/A9—heterodimeric calcium-binding protein, SAPK—stress-activated protein kinase, SAT—subcutaneous adipose tissue, SATB—special AT-rich binding protein 2, SCC—squamous cell carcinoma, SCF—stem cell factor, SCLC—small-cell lung cancer, SCOV-3—ovarian adenocarcinoma cancer cell line, SDF-1—stromal cell-derived factor-1, SERPIN E1—endothelial plasminogen activator inhibitor, sFRP1/2—secreted frizzled-related protein 1 or 2, SHH—Sonic hedgehog signaling, SMAD4—mothers against decapentaplegic homolog 4, SRFP2—secreted frizzled-related protein 2, SSEA4—stage specific embryo antigen 4, SSL CAF—steady-state-like cancer-associated fibroblast, STATs—signal transducer and activator of transcription proteins, STILs—stromal tumor-infiltrating lymphocytes, STK11—serine/threonine kinase 11, SULF1—sulfatase 1, TADCs—tumor associated dendritic cells, TAG—triacyloglycerol, TAGLN—transgelin, TAMs—tumor-associated macrophages, TANs—tumor-associated neutrophils, TAP—transporter associated with antigen processing, TC—thyroid cancer, tCAFs—tumor-like cancer-associated fibroblasts, Tcm—central memory T cells, Tconv—conventional T cells, TCR—T cell receptor, TCSC—thyroid cancer stem cell, TDLN—tumor-draining lymph nodes, TECs—tumor-associated endothelial cells, Tem—effector memory T cells, Texhausted—exhausted T cells, Tg—thyroglobulin, TGF-β—transforming growth factor β, TIM-3—mucin domain-containing protein 3, TIMP-1—tissue inhibitor of metaloproteinase 1, TILs—tumor-infiltrating lymphocytes, TKI—tyrosine kinase inhibitor, TLR—Toll-like receptor, TLS—tertiary lymphoid structures, TMB—tumor mutation burden, TME—tumor microenvironment, TMEM—transmembrane proteins, TNBC—triple-negative breast cancer, TNC—tenascin C, TNF-α—tumor necrosis factor alpha, TNFR2—tumor necrosis factor receptor 2, TPM1—tropomyosin alpha-1, TPO—thyroperoxidase, TRAIL—tumor necrosis factor (TNF)-related apoptosis-inducing ligand, TRAMP—transgenic adenocarcinoma of mouse prostate, Tregs—T regulatory cells, TrkB—tropomyosin receptor kinase B, Trm—tissue-resident memory T cells, TRP1/2—tyrosinase-related protein 1 or 2, TSC—thyroid stem cell, TSH—thyroid-stimulating hormone, TSH-R—thyroid-stimulating hormone receptor, TUBA1B—tubulin alpha 1b, TWIST1—twist-related protein 1, UA—ursolic acid, UC—ulcerative colitis, ULBP2—UL16-binding protein 2, VAT—visceral adipose tissue, vCAFs—vascular cancer-associated fibroblasts, VCAM-1—vascular cell adhesion molecule-1, VCAN—versican, VEGFA—vascular endothelial growth factor A, V-GCSC—gastric cancer stem cell, VHL—von Hippel–Lindau, VIM—vimentin, vWF—von Willebrand factor, WAT—white adipose tissue, WNT—Wingless-type signaling protein pathway, YAB—Yes-associated protein, and YKL-40—chitinase-3-like protein 1 (CHI3L1).
